# Physics‐Constrained Constitutive Learning of Rate‐Limiting Timescales for Efficient Hydrogen‐Based Direct Reduction for Green Steel Making

**DOI:** 10.1002/advs.75498

**Published:** 2026-05-04

**Authors:** Anurag Bajpai, Barak Ratzker, Pasquale Cavaliere, Dierk Raabe

**Affiliations:** ^1^ Max Planck Institute For Sustainable Materials Düsseldorf Germany; ^2^ Department of Innovation Engineering University of Salento Via per Arnesano Lecce Italy; ^3^ Process Metallurgy Research Unit Faculty of Technology University of Oulu Oulu Finland

**Keywords:** constrained additive model, direct reduced iron, hydrogen‐based direct reduction, shrinking‐core model, sustainable ironmaking, symbolic regression

## Abstract

Hydrogen‐based direct‐reduction enables carbon‐neutral primary ironmaking, yet widespread industrial adoption is constrained by sluggish late‐stage kinetics, which lower production efficiency and increase energy and hydrogen consumption. Here, we develop a conversion‐resolved constitutive framework that infers effective reaction and transport timescales directly from measured reduction trajectories and maps their constitutive dependence on operating conditions, pellet architecture, and composition. The scientifically constrained additive model (SCAM) framework is then used to convert these trajectory‐inferred timescales into interpretable constitutive maps, symbolic laws, and regime boundaries across variations in processing conditions and pellet microstructure/composition. We find that internal diffusion accounts for most of the incremental reduction time at intermediate to high conversion percentages, and the reaction‐to‐diffusion control boundary shifts systematically with conversion progression and evolving porous microstructure. Temperature and hydrogen partial pressure mainly accelerate early‐stage conversion rates, whereas the late‐stage conversion rates are governed by the pellet‐to‐pore length scale, average porosity, and tortuosity. Pellet composition primarily affects the late‐stage diffusion‐controlled regime through its influence on pore‐morphology descriptors, while a residual effect persists in the reaction‐controlled regime. The resulting regime maps and symbolic laws yield experimentally anchored pellet‐scale constitutive relations to identify reduction‐stage‐specific rate‐limitations and guide industrial pellet design, thereby providing actionable guidelines for more efficient green steelmaking.

## Graphical abstract

A conversion‐resolved constitutive framework is developed for the hydrogen‐based direct reduction of iron oxide pellets. Effective reaction and transport timescales are inferred directly from measured trajectories and mapped against operating conditions, pellet architecture, and composition. The analysis reveals how late‐stage transport control emerges and identifies actionable routes to shorten reduction time and improve hydrogen utilization.

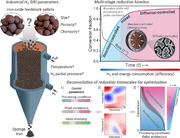

## 1 Introduction

Steel production and primary ironmaking remain among the most significant industrial contributors to anthropogenic CO_2_ emissions [1, 2]. Hydrogen‐based direct reduction has therefore emerged as a leading decarbonization route, replacing carbonaceous reductants with H_2_, yielding H_2_O rather than CO_2_ as the byproduct [3, 4, 5]. The key constraints for its sustainable deployment are not only the thermodynamic feasibility of reducing iron oxides, but hydrogen supply and utilization efficiency in direct‐reduced iron (DRI) production, which together determine the energy demand per ton of iron and the required hydrogen throughput [6, 7]. Stoichiometrically, complete reduction of hematite to metallic iron requires 54 kg H_2_ per ton of Fe, corresponding to an electricity input of ∼2.7–3.0 MWh per ton of Fe at 50–55 kWh kg^−1^ H_2_ [8]. At the scale of global crude steel production (e.g., 1892 Mt in 2023), the implied hydrogen requirement is on the order of approximately 100 Mt H_2_ per year, comparable to the total global hydrogen demand of about 97 Mt in 2023 [9]. These processing constraints make both hydrogen availability and process‐level hydrogen utilization efficiency critical bottlenecks for the widespread implementation of green steel production on a global scale [10].

**FIGURE 1:**
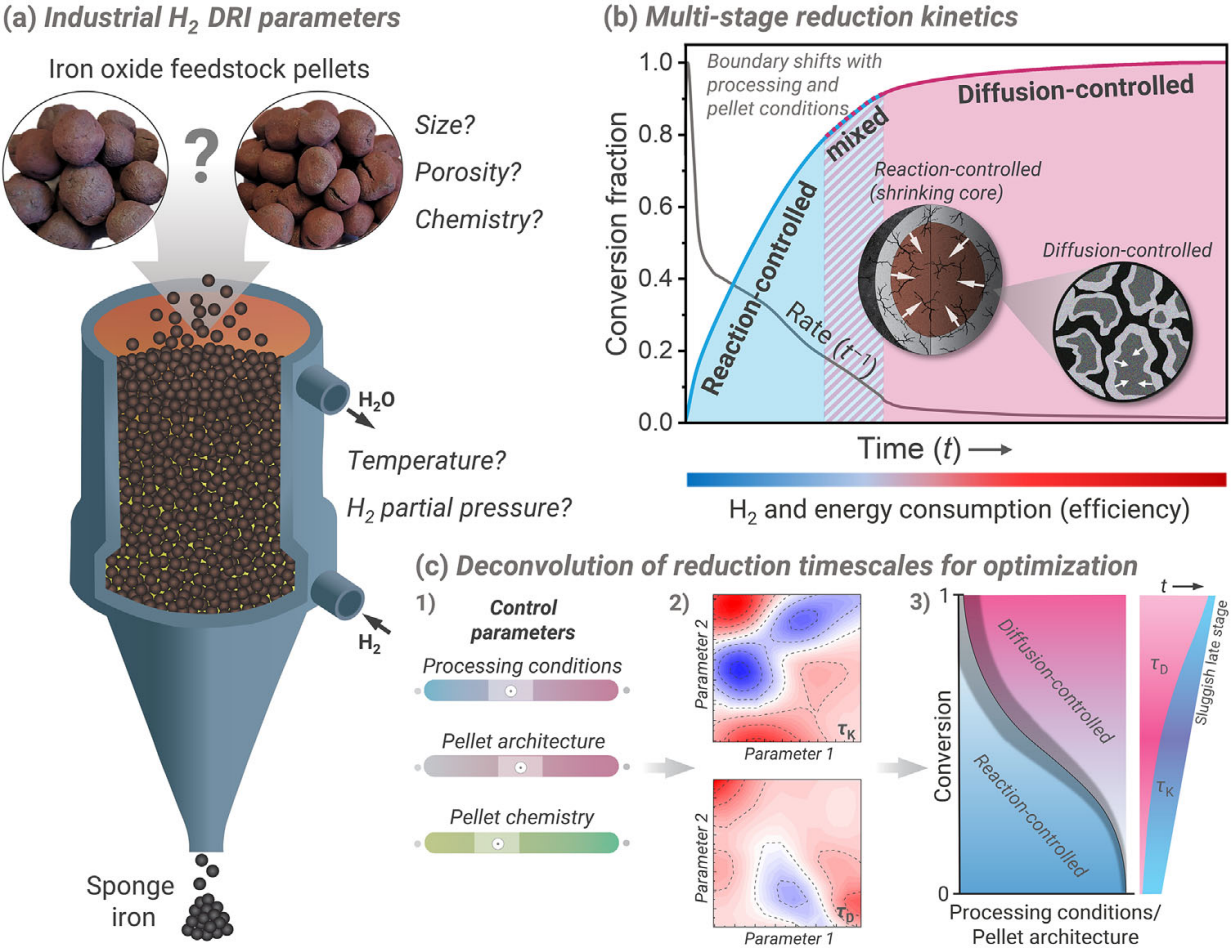
Conversion resolved kinetics summary and design outputs from the SCAM framework for efficient hydrogen‐based direct reduction of iron oxide pellets. (a) Schematic of a shaft furnace direct‐reduction process highlighting the coupled industrial operating and pellet design space. Operating variables, such as temperature and hydrogen partial pressure, act together with pellet characteristics, including size, porosity, and composition, to determine reduction behavior. (b) Multi‐stage reduction kinetics illustrated as conversion fraction versus time, showing a transition from reaction‐controlled at low to intermediate conversions to diffusion‐controlled at high conversions. The contributions of different rate‐controlling regimes shift with processing conditions and pellet architecture/composition, and late‐stage conversion (primarily from wüstite to iron) dominates the total time budget and, consequently, dictates overall process efficiency (which depends on hydrogen and energy consumption). (c) Central concept of our work: Measured reduction trajectories are deconvoluted into three effective characteristic timescales, namely *τ*_M_ for external mass transfer, *τ*_K_ for interfacial reaction, and *τ*_D_ for internal diffusion, and are then mapped back to the operating and pellet design space as compact, interpretable landscapes by the scientifically constrained additive model (SCAM) framework. These parameter maps quantify how operating conditions, pellet architecture, and pellet composition shift the relative magnitudes of external transfer, reaction, and internal transport limitations, thereby identifying where control changes along the reduction trajectory and which variables govern the late‐stage reduction tail to improve process efficiency.

In practice, the sustainability of H_2_‐based direct reduction is governed by hydrogen utilization and the total energy per ton of DRI [11]. However, the kinetic limitations for direct reduction on an industrial scale are often mitigated by increasing operating temperatures, increasing hydrogen chemical potential, and extending holding times. Yet, these practices increase energy consumption, hydrogen demand, and costs, thereby directly eroding the net sustainability benefit of large‐scale adoption of this technology [10, 12, 13]. Moreover, the near‐complete metallization of iron oxide (hematite) in H_2_ shaft furnaces requires the hydrogen supply well above the stoichiometric demand (i.e., Fe_2_O_3_ + 3H_2_ → 2Fe + 3H_2_O), with the total H_2_ supply typically ∼90‐130 kg per ton of DRI at 700–1000°C [11], while the chemically consumed hydrogen remains ∼54 kg per ton of DRI. The surplus hydrogen exists as unused hydrogen with the product water vapor and must be recovered via recycling and gas conditioning. This necessitates supplementary reactor electrical energy demands of ∼0.56–0.59 MWh per ton of Fe feed in the same operating window [11], incurring further costs and lowering overall process efficiency. These process efficiency limitations are amplified by the late‐stage conversion kinetic tail, where reduction rates can drop from ∼2%–5% min^−1^ between 20–80% conversion to ∼0.3%–1% min^−1^ above 90% conversion, consistent with the FeO (wüstite) → Fe step becoming extremely rate‐limiting [14, 15]. Therefore, there is a need for a kinetic framework that resolves pellet‐scale rate limitations across the reduction trajectory and serves as a constitutive basis for pellet design studies and reactor‐scale modeling of hydrogen‐based direct reduction. Figure 1a summarizes the degrees of freedom that furnace operators and pellet producers need to optimize: temperature and hydrogen partial pressure on the process side, and pellet size, porosity volume fraction, pore network connectivity, and composition on the feedstock side. Figure 1b highlights the central kinetic bottleneck: the rate‐controlling limitation is not fixed during reduction, and the late‐stage conversion tail can dominate the total time budget and therefore the energy and hydrogen requirements. Accordingly, a useful kinetic framework must quantify how operating variables and pellet composition, together with pellet architecture (pellet‐scale structural descriptors relevant to transport, including porosity, tortuosity, pore size, and pellet diameter), shape the entire reduction trajectory [11, 16, 17, 18], including the late‐stage that governs effective reduction time and hydrogen utilization.

Hydrogen reduction of iron oxides is a mixed‐control gas‐solid transformation in which the rate‐limiting contributions evolve with conversion degree, driven by phase transformations and microstructural evolution [19, 20, 21, 22, 23]. During the early stages of reduction, an abundant reactive surface is available for interfacial reaction, and microstructural changes such as pore formation, coalescence, and cracking can increase the connected porosity and expose reactive oxide surfaces, thereby governing the reduction trajectory primarily through interfacial reaction rates. In contrast, at higher conversion percentages, the remaining FeO‐rich regions become progressively encapsulated by the growing iron product and a less connected pore network (due to sintering and pore closure), so oxygen removal becomes increasingly constrained by internal transport through the product layer and the tortuous pore pathway [22, 24, 25, 26, 27]. This late‐stage, commonly referred to as the diffusion‐controlled regime, often dominates the total reduction time and directly affects hydrogen utilization [20, 28]. High‐resolution, multiscale studies also reveal strong spatial heterogeneity in reducibility within DRI pellets, including unreduced oxide islands, variable pore connectivity, and an evolving product‐layer topology [15, 29, 30, 31, 32, 33]. Pellet‐scale kinetics are, therefore, intrinsically conversion‐resolved, and a single global rate constant or a single controlling regime is generally insufficient for comparing pellet designs and operating conditions [34, 35].

Classical forward models for gas‐solid reduction, including shrinking‐core models (SCM) and related mixed‐control formulations grounded in Sohn's additive reaction time theory, provide physically interpretable rate expressions [36, 37, 38, 39]. Related additive time formulations have also been used in hydrogen‐based direct reduction and shaft furnace modeling [40]. These approaches are valuable because they retain an explicit physical partition of transport and reaction resistances. However, fully predictive treatment of pellet scale reduction under industrially relevant conditions remains difficult because chemical kinetics, internal transport, pore evolution, and sintering are strongly coupled and continuously modify the relevant length scales during reduction [26]. As a result, it remains challenging to translate these mechanistic insights into constitutive relations that remain actionable across multivariable operating conditions and realistic variation in pellet composition and architecture with a single parameter set [41].

Recent advances in interpretable machine learning provide constrained function classes that can represent such multivariable dependencies while enforcing physically motivated shape constraints, which is essential when outcomes are intended to support hydrogen allocation and operating policy [42, 43]. However, most data‐driven frameworks still prioritize predicting time or conversion, without retaining an explicit conversion‐resolved rate‐limiting partition that can be probed mechanistically and tested under held‐out pellet composition families and across structurally varying conditions. Consequently, for both basic research and industry, the central need is an operationally interpretable framework that identifies when control shifts from interfacial reaction to internal transport along the same reduction trajectory, and which processing conditions and pellet architecture parameters govern the late‐stage tail that dominates energy throughput and hydrogen utilization.

In this work, we develop a conversion‐resolved kinetic framework for hydrogen‐based direct reduction, formulated based on the measured reduction trajectory *t*(X). The framework builds on the established additive reaction‐time interpretation of mixed control, while shifting the inference problem from prescribed characteristic‐time expressions to experimentally inferred pellet‐scale effective timescales. Thermogravimetric mass‐loss data are transformed to a time‐conversion representation, and each trajectory is deconvoluted into non‐negative contributions associated with gas‐film interaction, interfacial chemical reaction, and internal diffusion through the evolving oxides and metal product layer. This formulation addresses a practical limitation of classical mixed‐control fitting that typically yields a single best‐fit regime or a fixed parameter set, without specifying at which point during reduction, rate‐control shifts from reaction (fast) to diffusion (slow), or which process conditions and pellet characteristics drive the late‐stage tail. The resulting rate‐controlling timescales are then expressed as intrinsically interpretable constitutive maps, i.e., experimentally anchored relations linking effective reaction and transport timescales to operating conditions, pellet architecture, and composition, using a scientifically constrained additive modeling (SCAM) framework. Physically motivated smoothness and monotonicity constraints are imposed to enforce metallurgically admissible dependencies on temperature, reducing gas atmosphere ($p_{H_{2}}$), pellet diameter, pore‐network descriptors, and composition, while retaining a minimal interaction structure. A stage‐resolved analysis further separates direct pellet composition effects from microstructure‐mediated pathways and supports transfer across held‐out pellet composition families within the sampled pellet‐scale descriptor space. Figure 1c summarizes our approach: conversion‐resolved time contributions are mapped back to processing conditions and pellet architecture descriptors using the SCAM framework, thereby revealing what primarily accelerates early conversion and what primarily shortens the sluggish late‐stage tail that determines the practical reduction time. The constitutive maps were further reduced to compact symbolic expressions, and the conversion resolved regime maps, whose validity is restricted to the experimentally sampled pellet‐scale envelope. Within the bounds of the experimental dataset used, the framework identifies which processing and pellet‐architecture variables accelerate early conversion and which shorten the late‐stage tail, which governs the practical pellet‐scale reduction time and resource utilization in hydrogen‐based ironmaking.

## 2 Results

### 2.1 Experimental Data and Trajectory Construction for Conversion‐Resolved Analysis

The experimental foundation for this study is a pellet‐scale hydrogen reduction dataset, compiled from several previously published studies [34, 35, 41, 44, 45, 46, 47], comprising 227 unique experiments and 17690 conversion‐time combination datapoints. The experiments spanned operating temperatures of 700–1050°C and gas pressures of 1–6.8 bar, including 194 runs in pure H_2_. Pellet diameter ranged from 5 to 18 mm. Porosity, tortuosity, pore size, and density were treated as measured inputs rather than fitted parameters. Here, porosity (ε) is the volume fraction of voids in the pellet, ε  = *V_pore_*/*V_total_*  (in %). Tortuosity (*τ*_tort_) is the geometric path‐length ratio for transport through the connected pore volume, τ_tort_ = *L*/*L*_Eu_ , computed from binarized X‐ray micro‐computed tomography volumes. *L* is the actual shortest path length through the connected pore network, and *L*_Eu_ is the straight‐line distance across the same start‐end points. The reported pore size *d*_pore_ denotes the mean equivalent pore diameter extracted from the segmented pore‐size distribution (equivalent‐sphere measure) [47]. Across the compiled dataset, these pellet‐architecture descriptors are available for each unique experiment at interrupted‐reduction states. The distribution of all operating processing conditions, as well as pellet composition and architectural variables, is reported in Figures S1–S4 and Table S1.

These experimentally sampled ranges define the validated domain of the present framework. All constitutive maps, inferred characteristic time‐scales, symbolic expressions, and regime assignments reported throughout the study are, therefore, restricted to the pellet scale descriptor space sampled by this dataset. The present analysis does not establish quantitative validity outside this bounded space, including for larger pellets (>18 mm), substantially different pellet architectures, or reactor‐scale environments.

Within this experimentally bounded domain, the analysis is directed toward the practical bottleneck in hydrogen‐based direct reduction: the late‐stage sluggish behavior, which often dominates total reduction time and forces high gas throughputs and operating costs. This bottleneck arises because reaction kinetics, internal transport, and gas access do not act independently. They co‐evolve with conversion progression through phase and microstructural evolution, so that changing parameters such as temperature or hydrogen pressure can improve interfacial kinetics while simultaneously penalizing internal transport by altering the pore network and product‐layer morphology. Two key questions arise in that context that need to be disentangled. First, given only the measured conversion trajectory X(*t*), can the pellet‐scale reduction time be expressed on the physically meaningful trajectory object *t*(X) in a manner that separates the contributions associated with gas‐film interaction, interfacial reaction, and internal diffusion, without introducing unmeasured internal state variables? Second, within the experimental envelope, which reducing conditions and pellet‐architecture descriptors shift these contributions as conversion progresses, and do the inferred trends remain transferable under pellet composition family holdouts?

The outcome is a conversion‐resolved set of effective timescales that account for pellet‐scale behavior under the adopted mixed‐control trajectory formulation. These quantities are not intended as phase‐resolved rate constants, spatially resolved transport fields, or pellet‐architecture‐independent material constants. They should therefore be interpreted as effective pellet‐scale descriptors within the sampled domain, rather than as universal kinetic parameters for direct extrapolation to larger pellets or shaft‐furnace conditions.

The primary kinetic observable used for trajectory inference is the mass‐loss‐derived conversion of the initial iron oxide into the iron product, X(*t*). In addition to this trajectory, the dataset contains pellet‐architecture measurements at interrupted reduction states across the experimental set, including porosity (fraction) and, where available, related descriptors such as tortuosity and pore size. However, these measurements are not introduced here as explicit time‐dependent internal state variables governed by a fitted structural evolution law. Instead, the analysis is carried out on the resolved time‐conversion object *t*(X), which enables comparisons of conversion windows and places mixed control on a common conversion coordinate. Microstructural evolution is therefore represented through interrupted‐state pellet architecture measurements (i.e., porosity fraction, tortuosity, pore size, density), together with the conversion dependence of the inferred effective behavior on *t*(X), without claiming a fully resolved microstructure history.

To make *t*(X) comparable across experiments, each X(*t*) curve is forced to be physically monotone, then inverted under explicit guardrails that prevent non‐physical experimental artifacts or noise. The smoothing and inversion checks are reported separately (Figures S8 and S9) to document that the subsequent mechanistic partition is built on the reduction trajectory object that is numerically stable and physically meaningful.

### 2.2 Conversion‐Resolved Mixed‐Control Deconvolution on Reduction Trajectories

The measured reduction curves, X(*t*), were transformed into the inverse trajectory object *t*(X), so that mechanistic changes with conversion can be analyzed directly on a common conversion coordinate rather than being compressed into a single fitted rate constant or a single dominant regime assumption (Figure S10). We then express each experiment by a small set of deconvoluted effective timescales that preserve the shape of *t*(X) while remaining comparable across conditions. The deconvolution targets three physically distinct rate‐controlling mechanisms: external gas‐film interaction, interfacial chemical reaction, and internal diffusion through the evolving porous/product‐layer region. The timescales quantify how the process time budget is divided between these over the same conversion interval, without conflating early and late reduction behavior into a single global parameter. This reformulation is motivated by a practical bottleneck. In industrial hydrogen‐based direct reduction, the late conversion tail often accounts for most of the total reduction time, unnecessarily inflating energy and hydrogen costs. The conversion‐resolved timescales therefore provide reduced‐order quantities that can be mapped against processing conditions as well as pellet architecture and composition to identify which rate‐limiting contribution is responsible for the prolonged late‐stage reduction tail.

**FIGURE 2:**
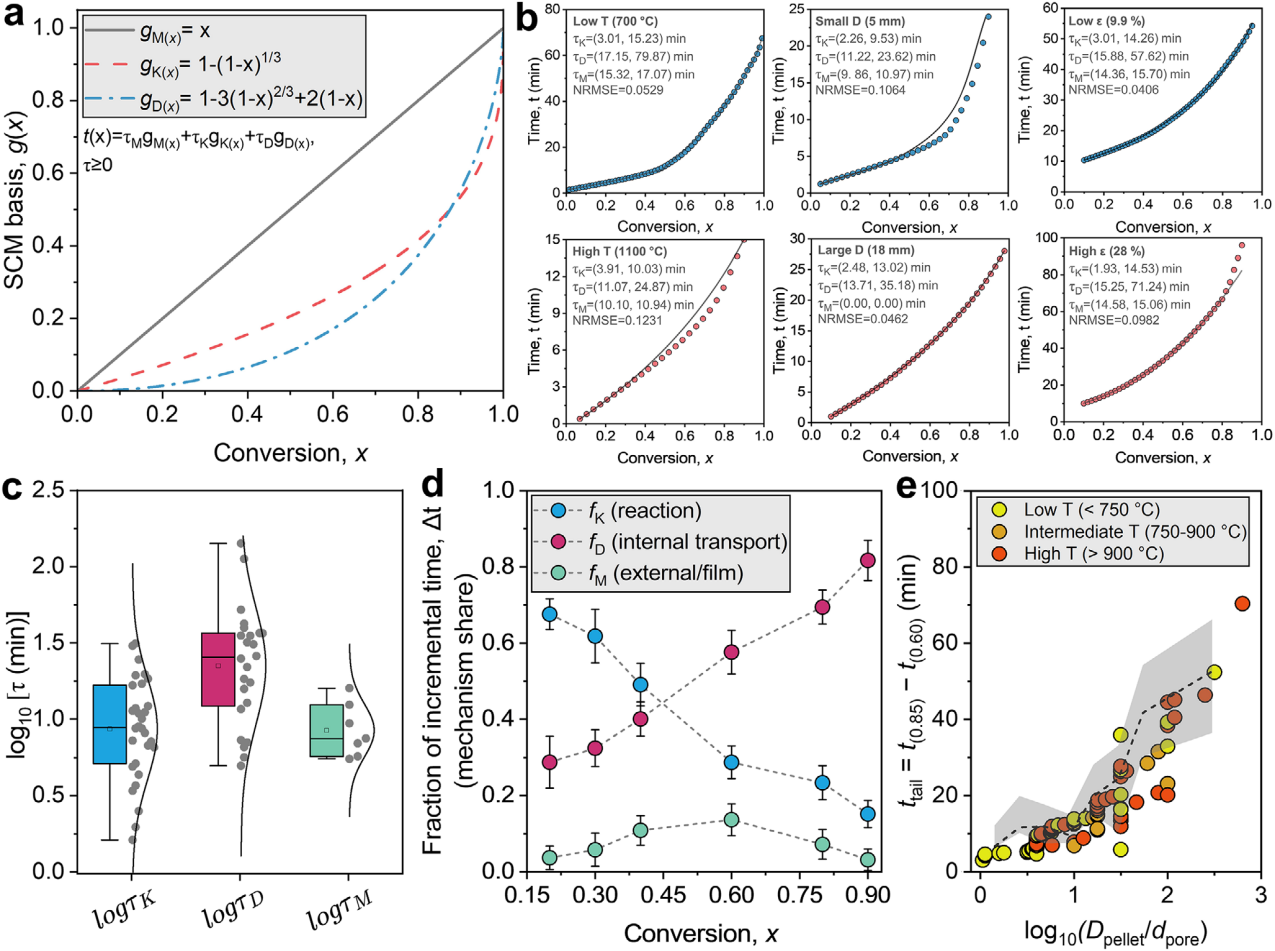
Trajectory‐inferred additive‐time deconvolution of hydrogen reduction and the physical interpretation of the inferred timescales. (a) Fixed SCM conversion bases *g*_M_(*x*), *g*_K_(*x*), and *g*_D_(*x*) used to represent the measured conversion‐coordinate *t*(*x*) as an additive sum of external access, gas–solid interfacial reaction, and effective pellet‐scale internal transport contributions: *t*(*x*) = *τ*_M∙_ *g*_M_(*x*)+*τ*_K∙_ *g*_K_(*x*)+*τ*_D∙_ *g*_D_(*x*) with *τ_i_* ≥ 0. The *τ* parameters are trajectory‐inferred timescales (units of time) obtained from *t*(*x*), not fitted rate constants from a prescribed kinetic law. (b) Representative reconstructions of *t*(*x*) across distinct operating and DRI pellet architectural conditions, showing that the single‐stage SCM deconvolution captures the full trajectory shape while returning one experiment‐specific triplet *τ*_M_, *τ*_K_, and *τ*_D_ under an identical extraction protocol. The explicit stage‐resolved analysis is introduced only in the subsequent two‐stage decomposition, where the trajectory is partitioned at X = 0.60 into an early stage (X ≤ 0.60) and a late stage (X > 0.60). (c) Distribution of inferred timescales across the dataset, illustrating the separation between reaction and diffusion rate‐control limitations. (d) Conversion‐resolved partitioning of incremental time Δ*t* ($f_{i}\;\left(x\right)=\frac{dt_{i}/dx}{dt/dx}$, i∈{M,K,D}, and *dt_i_*  =  τ_*i*_ *dg_i_*) into reaction, internal diffusion, and external/film fractions, demonstrating that the dominant rate‐controlling mechanisms shift with conversion progression as the effective interfacial and internal transport state evolves during reduction. Time fractions sum to 1 for all conversion fractions. (e) Late‐stage time penalty *t*_tail_ = *t*(0.85)−*t*(0.60) versus the pellet architecture ratio *D*_pellet_/*d*_pore_, highlighting the role of the effective pellet‐scale internal transport length relative to pore scale in governing late‐conversion rates.

We represent the conversion‐resolved time *t*(X) through an additive‐time mixed‐control basis,

$$t\;\left(X\right)=\tau_{M}g_{M}\left(X\right)+\tau_{K}g_{K}\left(X\right)+\tau_{D}g_{D}\left(X\right),\to \tau_{M},\tau_{K},\tau_{D}\geq 0,$$

where *τ*_M_, τ_K_, and *τ*_D_ are non‐negative rate‐limiting timescales (min) and *g_i_* (*X*) are fixed conversion‐basis functions (Figure 2a). The three terms correspond to physically distinct rate‐controlling contributions at the pellet scale. *τ*_M_ captures gas‐phase mass transfer and near‐surface access limitations, *τ*_K_ captures interfacial chemical reaction limitations, and *τ*_D_ captures the effective pellet‐scale internal mass‐transport limitation associated with diffusion through the evolving porous product layer. In the present formulation, this term represents the net consequence of internal transport resistances on the measured reduction trajectory and does not resolve inter‐grain, inter‐crystal, and intra‐crystal diffusion as separate characteristic times [40].

Non‐negativity is imposed to prevent non‐physical cancellation between terms. A fitted value at the boundary *τ* = 0 indicates that the corresponding contribution is negligible within the analyzed conversion window at the resolution supported by the data, and not that the underlying physical process is absent (Table S5). The basis functions are deliberately distinct in shape so that gas‐film‐, reaction‐, and diffusion‐governed curvature in *t*(X) can be separated using only three amplitudes per trajectory (Figure 2a; Figure S11a).

The adequacy of this representation is established by reconstructing *t*(X) across representative extremes of the dataset, including low versus high temperature, small versus large pellets, and low versus high porosity (Figure 2b). A single mechanistic basis reproduces the observed curvature, including the marked increase in incremental time at high conversion percentages, characteristic of emerging internal transport rate‐control limitations at the pellet scale. Per‐experiment timescales and reconstruction error metrics are reported in Table S5.

It is important to note that the representative reconstructions shown in Figure 2b are based on a single‐stage SCM decomposition, in which the full reduction trajectory is represented by one experiment‐specific triplet of non‐negative timescales τ_M_, τ_K_, and τ_D_. In this form, the additive‐time basis serves as a whole‐trajectory description that captures the dominant curvature of *t*(X) across the experimental envelope. The stage‐resolved treatment is introduced subsequently, when the trajectory is partitioned to reflect the systematic reorganization of reaction and internal transport limitations as reduction progresses.

Residuals plotted as a function of conversion percentage show no systematic drift, and reconstruction errors remain comparable across temperature and pellet‐architecture bins (Figure S12). This behavior indicates that the three‐term accounting captures the dominant curvature across the conversion range without requiring additional ad hoc terms. Importantly, this reconstruction is achieved with a fixed basis and only three non‐negative amplitudes per experiment, which bounds model flexibility while allowing mixed control to emerge from the relative magnitudes of the *τ*_M_, *τ*_K_, and *τ*_D_ timescales.

The inferred timescales indicate that internal diffusion is not a minor correction within the present experimental dataset. Specifically, the distribution of log_10_ *τ*_D_ is shifted to larger values relative to log_10_ *τ*_K_ and log_10_ *τ*_M_, and exhibits substantial dispersion (Figure 2c). Conversion‐resolved fractions of incremental time further show that internal diffusion contributes most strongly during the late conversion interval, whereas reaction control is more prominent in earlier stages, and this balance shifts with conversion progression, while the external gas‐film transfer contribution is comparatively negligible throughout the reduction timescale (Figure 2d). Consistent with this mechanistic interpretation, a late‐stage tail metric defined as *t*_tail_ = *t*(0.85)–*t*(0.60) increases with the pellet architecture metric log_10_ (*D*_pellet_/*d*_pore_) in Figure 2e. Here, *X* = 0.60 was used as the lower bound because it defines the dataset‐wide onset of the latestage adopted throughout the two‐stage SCM analysis, on physical grounds related to the conversion‐dependent reorganization of interfacial reaction and internal diffusion contributions. Similarly, *X* = 0.85 was chosen as a high conversion level that captures the late‐stage slowdown while avoiding over reliance on the most terminal portion of the trajectory. This scaling is consistent with the expectation that, as reduction proceeds, the evolving pore network and growing product layer increasingly hinder internal oxygen transport through longer diffusion paths, resulting in pronounced sluggish kinetics in the late stages of reduction.

These results motivate an explicit early‐ and late‐stage split on physical grounds. For all experiments, this stage partition was imposed using a fixed conversion threshold of X = 0.60, with the early stage corresponding to X ≤ 0.60 and the late stage to X > 0.60. The accessible interfacial area and the effective internal transport length scale evolve with conversion percentage due to the formation of metallic iron, pore‐network coarsening or constriction, and sintering‐driven densification, all of which alter the relative importance of reaction vs. diffusion as reduction progresses [20, 48, 49]. Accordingly, a single‐stage triplet *τ*_M_, *τ*_K_, and *τ*_D_ is not expected to represent the earlier reaction‐dominated and later transport‐dominated portions of the reduction path with equal fidelity across the full conversion range. This expectation is confirmed by the comparison between two‐stage and single‐stage SCM representations, where two‐stage SCM decomposition yields systematically lower reconstruction error than the single‐stage fit, with ΔNRMSE = NRMSE_single_–NRMSE_two‐stage_ remaining predominantly positive, concentrated largely below 0.01, and extending to approximately 0.05 (Figure S13a). The held‐out RMSE distribution is likewise shifted downward for the two‐stage representation relative to the single‐stage fit (Figure S13b). In addition, the fitted early‐ and late‐stage timescales also differ in a structured manner rather than being dominated by random scatter, as shown by the systematic deviation in Figure S13c. The single‐stage decomposition is therefore retained as the most compact whole‐trajectory representation, whereas the two‐stage formulation is used subsequently for stage‐resolved constitutive analysis. In this form, the two‐stage SCM decomposition captures the kinetic consequences of structural evolution along the pellet reduction path through the reorganization of effective reaction and internal‐transport contributions with conversion, although the underlying microstructural evolution itself, including pore‐network rearrangement, sintering progression, cracking, and product‐layer development, is not represented through explicit structural evolution equations.

Benchmarking against another classical pellet scale trajectory model further clarifies the role of the SCAM framework. Relative to the random pore model (RPM), our two‐stage SCM decomposition reproduces the measured reduction trajectories more accurately across the dataset, with median reductions in NRMSE of 0.0045 and in held‐out MAE of 0.1098 min across 179 experiments (Figures S14 and S15). These results support two‐stage SCM as the more suitable mechanistic basis for the present *t*(*X*) dataset. Further, the role of the SCAM framework extends beyond trajectory decomposition, by treating the SCM‐inferred characteristic timescales as physically interpretable targets and mapping them as constitutive functions of operating conditions, pellet architecture, and composition. This reveals how the hierarchy of reaction and transport limitations reorganizes along the reduction path within the experimental envelope, enabling uncertainty‐calibrated timescale prediction, transfer across held‐out pellet‐composition families, and conversion‐resolved regime identification beyond what can be obtained from trajectory fitting alone.

### 2.3 Physically Constrained Constitutive Maps for Reaction and Transport Mechanisms

In a hydrogen‐based reduction, the practical kinetic objectives are a short, predictable reduction time together with the suppression of the late‐conversion tail associated with the wüstite‐to‐iron stage. These features strongly influence hydrogen utilization and residence time requirements in shaft furnace operation. The reduction trajectory deconvolution yields two dominant timescales that directly reflect these operational constraints. The reaction‐controlled timescale *τ*_K_ reflects interfacial chemical reduction, while the transport‐controlled timescale *τ*_D_ reflects the effective pellet‐scale internal transport limitation that dominates the late tail. In the present formulation, *τ*_D_ is a net pellet‐scale transport descriptor and does not partition the internal transport burden into separately resolved inter‐grain, inter‐crystal, and intra‐crystal contributions. Accordingly, variation in *τ*_D_ should be interpreted as the net pellet‐scale consequence of internal transport resistance on the measured reduction trajectory, rather than as evidence for a uniquely identified internal diffusion sub‐mechanism. The third component *τ*_M_, associated with external gas‐phase mass transfer, is not carried forward into constitutive mapping because it is consistently negligible within the present experimental envelope and contributes minimally to the conversion‐resolved time budget relative to *τ*_K_ and *τ*_D_ (Figure 2d and Table S5). The subsequent constitutive analysis, therefore, focuses on *τ*_K_ and *τ*_D_, which govern the early and late‐stage reduction kinetics, respectively, and define the total reduction time.

The purpose of constitutive mapping is to express the SCM‐inferred timescales, *τ*_K_ and *τ*_D_, as functions of processing conditions and pellet architecture using a function form that is interpretable, physically constrained, and transferable within the sampled envelope. This is achieved with a scientifically constrained additive model, termed SCAM, in which log_10_ *τ* is represented as a sum of smooth one‐variable functions with selected two‐variable interaction terms. Monotonicity is imposed where the physics requires a fixed directional response over the experimental envelope, and non‐monotonicity is permitted only where competing metallurgical effects can plausibly generate a maximum or minimum. Smoothness regularization is used to prevent the emergence of spurious relations. Implementation details of basis construction, constraint encoding, and regularization selection are provided in the Methods and Sections S1 and S2.

A brief guide is practical before discussing the constitutive maps. A main‐effect curve shows how *τ*_K_ or *τ*_D_ change as a single control variable (e.g., temperature, porosity, etc.) is varied across the measured envelope, while the remaining variables are held constant at a representative operating point (median values here). It is therefore an experimentally anchored sensitivity of the effective rate‐controlling timescale to that variable. An interaction surface is used when the data indicate non‐separable behavior, meaning that the effect of one variable depends on another variable. In metallurgical terms, it highlights coupled control, for example, when pellet size sensitivity depends on pore‐network state.

**FIGURE 3:**
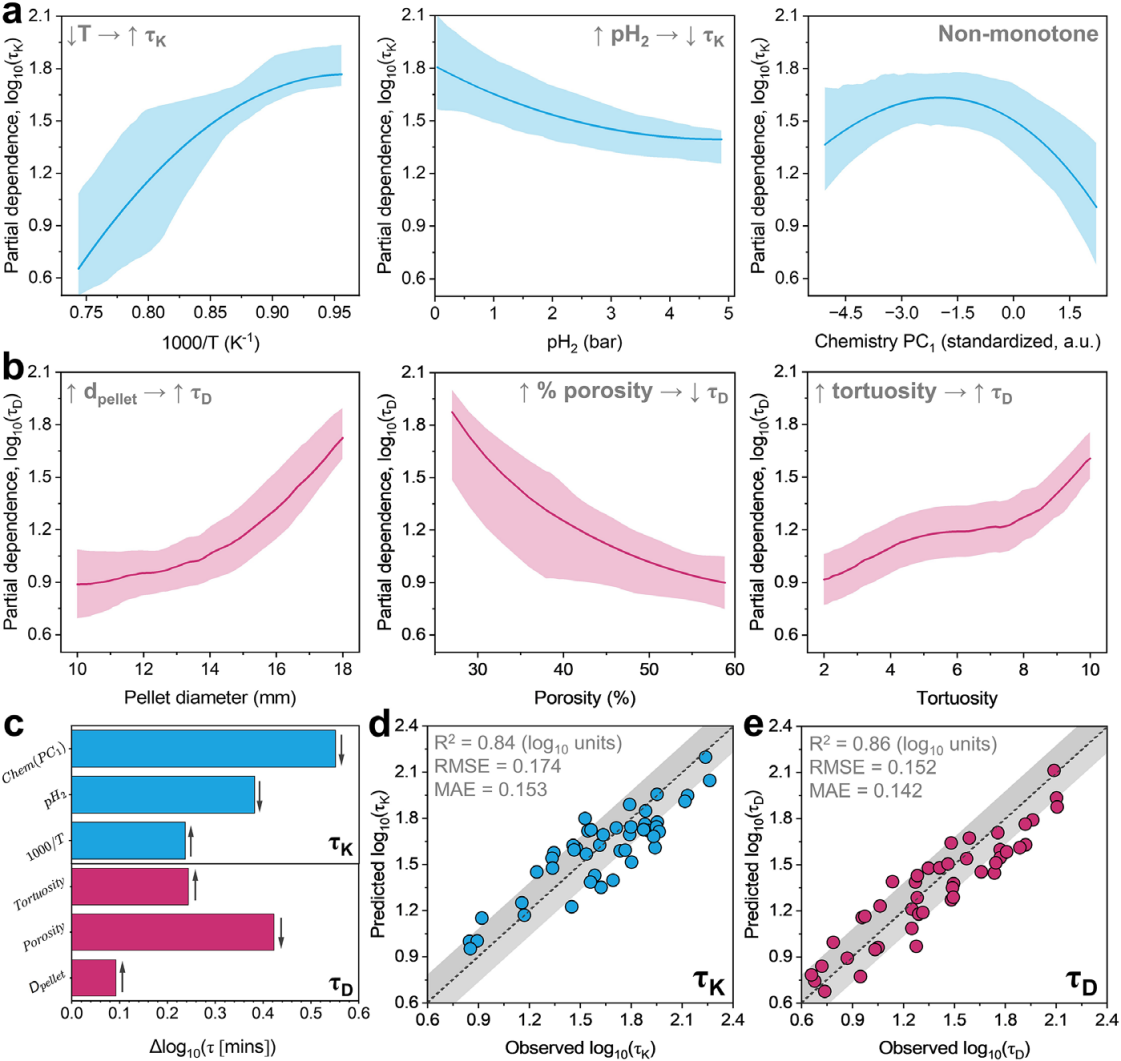
Interpretable, physics‐consistent mappings from processing, gas composition, and pellet architecture to the trajectory‐inferred rate‐controlling timescales. (a) Reaction‐controlled timescale *τ*_K_​ is expressed in the conversion coordinate as partial‐dependence responses to the primary thermochemical drivers. Increasing 1/T (lowering the temperature) extends *τ*_K_, consistent with slower interfacial reduction kinetics at lower temperatures. Increasing hydrogen fraction ($p_{H_{2}}$)​ shortens *τ*_K_ by enhancing the reducing potential. The low hydrogen end shown here corresponds to a CO‐rich reducing atmosphere within the compiled dataset, not to the absence of reductant, and should therefore not be interpreted as the asymptotic pH_2_→0 limit of a pure hydrogen system. The pellet composition coordinate (Chemistry PC_1_) summarizes compositional variation in the pellet and captures systematic, nonlinear shifts in the effective reaction rate control across compositions at otherwise comparable conditions. (b) Internal transport timescale *τ*_D_​ mapped against the dominant architectural controls. Larger pellet diameter increases *τ*_D_​; higher porosity fraction reduces *τ*_D_; higher tortuosity increases *τ*_D_​. (c) Relative driver importance, summarized as the change in inferred log_10_(*τ*) across each variable's physically observed range, separating reaction‐controlled parameters (i.e., temperature, $p_{H_{2}}$​​, pellet composition) from diffusion‐controlled parameters (i.e., porosity, tortuosity, pellet diameter). (d–e) Agreement between observed and predicted timescales for *τ*_K_ and *τ*_D_​, demonstrating that the constrained, interpretable model reproduces the experimentally inferred rate‐controlling landscape while maintaining physically meaningful directions of effect. In both panels a and b, the shaded regions denote bootstrap uncertainty envelopes of the learned partial dependence trends, obtained from experiment‐level resampling of the full inference pipeline.

The fitted timescales represent effective pellet‐scale rate limitations inferred from the full reduction trajectories and therefore incorporate the net influence of microstructural evolution on the measured conversion response. The compiled dataset contains interrupted‐state pellet‐architecture measurements, including porosity and related transport descriptors, but sintering, pore closure, cracking, evolving interfacial area, and pore network topology are not resolved here through explicit time‐dependent state variables or fitted structural evolution equations. The constitutive relations in Figure 3 should therefore be interpreted as experimentally anchored pellet scale trends valid only within the sampled descriptor envelope, not as intrinsic rate constants or transport coefficients, transferable without qualification across pellet sizes, pore architectures, feed compositions, or reactor environments.

Figure 3a summarizes the constitutive trends for reaction‐controlled timescale *τ*_K_. The dominant dependence is on temperature, consistent with thermally activated interfacial reduction kinetics. When expressed as a function of 1/T, the recovered trend is approximately linear over the envelope, consistent with an Arrhenius‐like response for an effective reaction limitation at the pellet scale. The dependence on the gas atmosphere is likewise physically consistent within the sampled reducing gas envelope. Increasing hydrogen fraction (represented here through $p_{H_{2}}$ = $f_{H_{2}}\times P_{gas}$) shortens *τ*_K_, consistent with stronger hydrogen‐driven reducing potential and reduced susceptibility to local product gas inhibition at the interface. This response should be interpreted within the experimentally sampled H_2_‐to‐CO‐reducing gas space. Quantitatively, the gas atmosphere effect is smaller than the temperature effect, confirming that temperature is the primary determinant of intrinsic kinetics while gas composition provides a secondary modulation.

The composition coordinate exhibits a non‐monotone dependence in Figure 3a. This behavior is expected as pellet composition influences reduction through multiple coupled pathways rather than a single scalar mechanism. In industrial pellets, gangue components can influence reducibility and microstructural evolution by potentially affecting evolving microstructural characteristics such as local oxide phase assembly (including the formation of more stable mixed oxides, such as iron silicates), fine‐pore network formation, the extent of sintering, and the morphology of metal product regions [50, 51, 52, 53]. A non‐monotone response is therefore interpreted as a net outcome of competing effects within the envelope, and it is not forced into a monotone form unless the mechanistic direction is unambiguous.

Figure 3b summarizes the constitutive trends for diffusion rate‐controlled timescale *τ*_D_. The dominant controls are geometric and architectural. *τ*_D_ increases with pellet diameter (*D*_pellet_), consistent with an increased characteristic transport length. *τ*_D_ decreases strongly with porosity (*ε*), consistent with increased effective diffusivity and improved pathway connectivity. *τ*_D_ also increases with tortuosity, consistent with more convoluted transport pathways leading to sluggish diffusion and, therefore, a longer diffusion timescale. The relative ordering of these effects is crucial because it identifies design parameters that can be targeted directly. Within this envelope, porosity provides the most substantial single control over the diffusion‐controlled timescale, while tortuosity and pellet size provide additional, mechanistically distinct leverage.

The effect of temperature on the diffusion rate‐controlled timescale *τ*_D_ map requires careful interpretation. Generally, diffusivity increases with temperature; however, in iron oxide direct reduction, the dominant temperature effect on internal oxygen transport is often indirect, mediated by reduction‐driven microstructural evolution. At relatively high temperatures (≥ ∼850°C), sintering can reduce porosity, alter tortuosity, and degrade connectivity, thereby increasing the effective hindrance to diffusion despite the increase in intrinsic diffusivity. In the present formulation, this pathway is captured through the measured pellet architecture descriptors rather than by assigning a direct Arrhenius meaning to *τ*_D_. Consistent with this, once porosity and tortuosity are accounted for, the remaining direct temperature dependence in the *τ*_D_ map is secondary within the studied window.

The summary panel in Figure 3c consolidates the hierarchy of processing and pellet architectural parameters. For *τ*_K_, temperature is dominant, hydrogen partial pressure is secondary, and pellet composition provides a smaller but systematic contribution. For *τ*_D_, pellet architecture controls diffusion rate‐governing regime, with porosity and tortuosity as dominant parameters, and pellet diameter introduces a secondary constraint. These constitutive maps provide the mechanistic basis for the interaction analysis that follows, where coupling terms are introduced to represent physically plausible non‐separable behavior across the measured envelope.

Figure 3d,e quantify the accuracy with which the constrained constitutive maps reproduce the deconvoluted timescales across the whole dataset. Each point compares the observed log_10_ (*τ*) obtained from trajectory deconvolution with the corresponding log_10_ (*τ*) predicted by the SCAM map under the same experimental conditions. The clustering of points around the 1:1 line indicates that the constitutive timescale descriptions capture most of the experiment‐to‐experiment variation while remaining physically constrained. These parity plots should be interpreted as evidence that the constrained maps provide a faithful and interpretable constitutive summary of the extracted reduction mechanism timescales.

**FIGURE 4:**
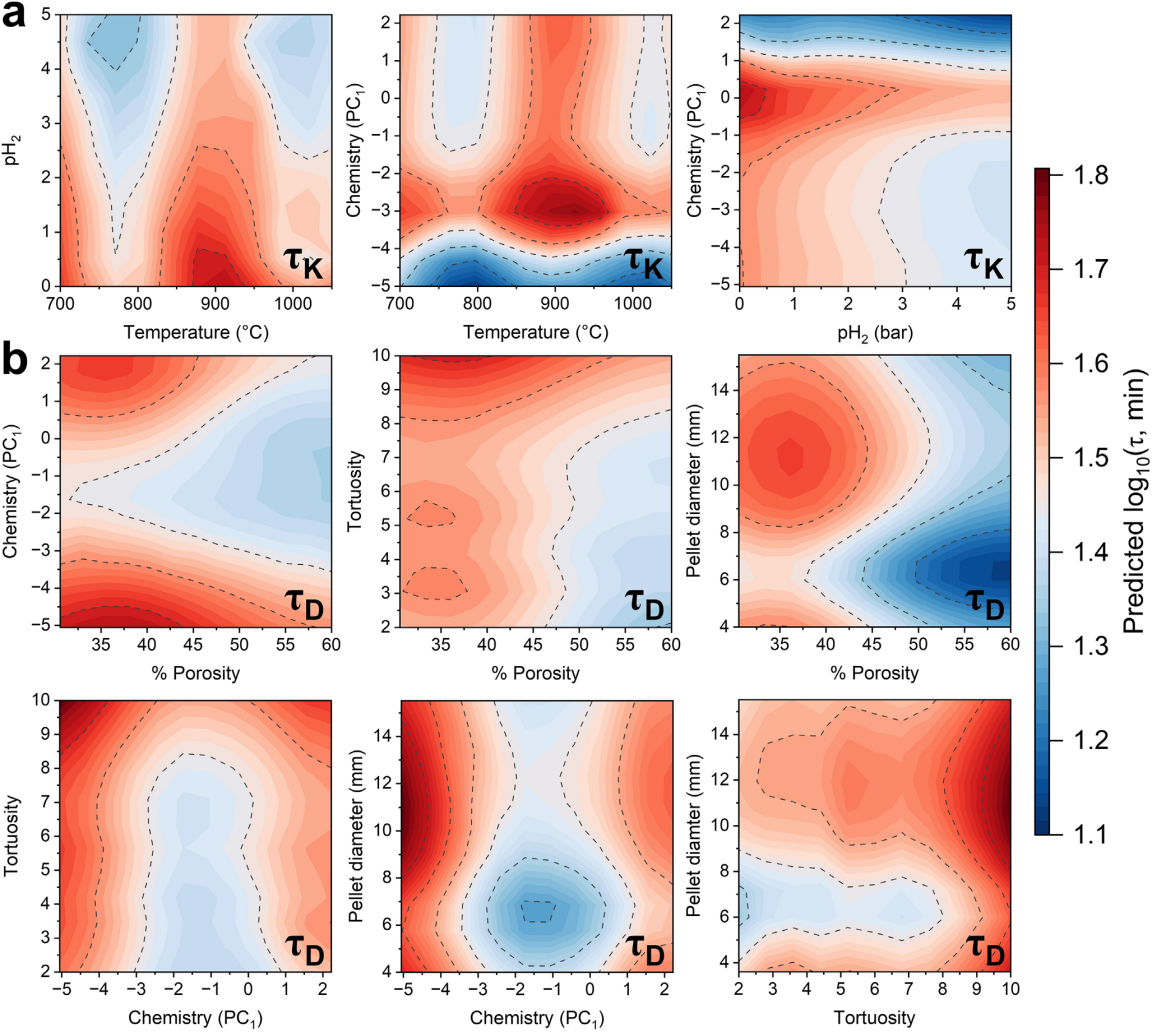
Coupled thermochemical and architectural controls revealed by interaction surfaces for reaction and transport timescales. 2D response surface maps summarize how the trajectory‐inferred additive‐time timescales vary jointly with key processing and structural descriptors, expressed as predicted log_10_(*τ*) in minutes (color scale; dashed contours denote iso‐timescale lines). (a) Reaction‐controlled timescale *τ*_K_ as a function of temperature and $p_{H_{2}}$​ (left), temperature and pellet composition PC_1_ (middle), and $p_{H_{2}}$​ and Chemistry PC_1_ (right), highlighting coupled thermochemical‐compositional sensitivity of the interfacial oxygen‐removal step. (b) Internal oxygen diffusion timescale *τ*_D_ as a function of porosity coupled with Chemistry PC_1_ (left), tortuosity (middle), and pellet diameter (right), together with additional coupled dependencies between tortuosity, pellet diameter, and composition (bottom row), revealing how pore‐network architecture and pellet diameter jointly set the effective diffusion resistance through the evolving product layer and connected porosity. Surfaces are evaluated within the experimentally sampled envelope, with all non‐displayed variables held at representative values (distribution medians) to isolate the coupled dependence shown in each panel.

The main‐effect maps (Figure 3) answer one specific question: how a timescale changes when one variable is varied while the others are held constant at their typical (median) values. This view is incomplete when two variables act on the same physical bottleneck. In hydrogen‐based direct reduction, both the interfacial composition and the evolving pore network are driven by coupled factors. For instance, varying the temperature increases intrinsic thermally driven kinetics but eventually also accelerates sintering, which hinders reduction kinetics. The interaction maps in Figure 4 are therefore used to answer the pertinent question: when one parameter is changed, does the sensitivity to a second parameter strengthen, weaken, or reverse? In other words, the interaction surfaces identify where the process response depends jointly on two coupled variables, a property that cannot be inferred from 1D trends alone. The interaction terms are retained when they remain stable under resampling and in the pellet composition family holdouts, ensuring the surfaces represent reproducible mechanistic coupling within the experimental envelope (Table S8).

For *τ*_K_, the dominant coupling is between temperature and $p_{H_{2}}$ (Figure 4a). This surface indicates that the effect of increasing hydrogen potential is not uniform across different temperatures. The response is strongest in the lower‐temperature region, where the interfacial reaction step remains rate‐limiting over a wider conversion span. Therefore, increasing $p_{H_{2}}$ produces a clearer reduction in *τ*_K_. At higher temperatures, the incremental benefit of higher $p_{H_{2}}$ diminishes because the effective bottleneck shifts away from the pure interfacial driving‐force term toward conversion‐evolving constraints associated with product‐layer development and sintering. The blue trough regions therefore identify joint operating‐condition combinations at which the inferred reaction timescale *τ*_K_ is minimized within the sampled domain, reflecting genuine interaction rather than the simple superposition of two independent main effects.

The pellet composition‐coupled *τ*_K_ interaction surfaces can be interpreted as follows. PC_1_ is a compact pellet composition coordinate that increases with Fe content and decreases with total oxide additions, so moving to lower PC_1_ corresponds to a higher gangue fraction. The surface maps (Figure 4a) show how the benefit of changing temperature and $p_{H_{2}}$ depends on the pellet composition state. This provides information about the composition‐dependent evolution of phases, gangue distribution, and product‐layer topology. However, interpreting the dependence of *τ*_K_ on pellet composition is not necessarily straightforward, as gangue elements or inclusions can also vary in size, morphology, and distribution. The flattening of the surface at very low PC_1_ indicates that once gangue content is high, changes in temperature and $p_{H_{2}}$ have a reduced ability to prolong the more effective interfacial reaction regime due to eventual microstructural changes.

In contrast, for *τ*_D_, the strongest couplings are architectural (Figure 4b). The porosity‐pellet diameter surface shows that increasing pellet size incurs a significantly larger diffusion limitation when porosity is low, which is consistent with longer internal diffusion lengths through denser product layers, thereby increasing the dependence on diffusion rates. Conversely, at higher porosity, the limitation is partially mitigated because easily accessible pore networks promote shorter characteristic diffusion pathways, mitigating the extent to which the product layer isolates the remaining oxide. The porosity‐tortuosity and pellet diameter‐tortuosity surfaces show that tortuosity cannot be treated as a constant correction; its impact varies as the system approaches a connectivity‐limited regime, where small changes in pathway convolution translate into significant changes in effective transport length.

The robustness analyses support that the inferred couplings are not artifacts of the flexible function class. Interaction screening and effect magnitudes recover the same physically expected pairings and remain secondary to the dominant main effects, indicating that the interaction terms refine the mechanistic insights obtained from main effects (Figures S16 and S17). Sensitivity checks on model parameterization and pooling structure demonstrate that the recovered surfaces are stable to reasonable choices of these parameters. In contrast, control tests in which constraints were removed or key descriptors were permuted degrade the structure and predictive behavior (Figures S18–S21; Tables S9 and S10).

Operationally, these interaction maps translate into control variables for operation and pellet design. Temperature and hydrogen partial pressure act primarily as coupled early‐stage controls for *τ*_K_, whereas pellet architecture variables act as coupled late‐stage controls for *τ*_D_, with the most significant rate‐control arising from the combination of large pellet diameter and low connected porosity.

Table 1 summarizes quantitative evidence that the SCM basis is adequate for trajectory‐level decomposition and that SCAM‐derived constitutive maps remain reliable under transfer across held‐out pellet composition families and model classes. The SCM representation reconstructs the measured trajectories *t*(X) with low error across the dataset. For 227 experiments, the median reconstruction RMSE is 0.229 min with an interquartile range of 0.134–0.345 min, indicating that a single mixed‐control additive‐time basis effectively captures the dominant curvature of the reduction trajectories. Complementary benchmark comparisons against alternative trajectory‐level formulations further show that the adopted two‐stage SCM representation improves upon a single‐stage SCM fit and outperforms the random pore model in reconstructing *t*(*X*), as summarized in Figures S13–S15. On this basis, the constitutive maps for log_10_ (*τ*_K_) and log_10_ (*τ*_D_) generalize across pellet composition family hold‐out, with log‐scale errors that are small relative to the envelope‐wide spread in the targets. The family‐out median RMSE and MAE are 0.315 and 0.282 for log_10_ (*τ*_K_), and 0.410 and 0.363 for log_10_ (*τ*_D_). Predicted uncertainty is calibrated on held‐out compositions, with median empirical coverage close to nominal for both the 90% and 95% intervals. These diagnostics establish reliability for interpolation and transfer across held‐out pellet composition families within the sampled pellet‐scale envelope. However, they should not be interpreted as quantitative uncertainty calibration for extrapolation beyond the observed support, including larger pellets or shaft furnace scale flow and heat transfer conditions. Further, a lattice surrogate produces similar out‐of‐fold error, and the resulting regime assignments are consistent. Across 198 experiments, log_10_ (*τ*_K_) out‐of‐fold (OOF) RMSE is 0.296 for SCAM versus 0.317 for the lattice model, and log_10_ (*τ*_D_) RMSE is 0.430 vs. 0.443, while regime agreement is 0.864 with κ = 0.835.

**TABLE 1:** Quantitative hierarchy of mechanistic trajectory reconstruction and SCAM timescale generalization performance. Summary of (*i*) SCM trajectory reconstruction fidelity for the conversion‐time representation *t*(X) (number of experiments fit and median RMSE with interquartile range), (*ii*) SCAM generalization of the inferred conversion resolved rate controlling timescales under pellet composition family holdout, reported as median RMSE/MAE and empirical coverage of nominal 90% and 95% prediction intervals, and (*iii*) robustness to discretization/lattice sensitivity, quantified by out‐of‐fold RMSE for SCAM vs. a lattice‐based surrogate, along with agreement statistics for the inferred mechanistic regime assignment (agreement rate, Cohen's κ, and early/late disagreement fractions).

Metric	Value
SCM trajectory reconstruction quality	
Number of experiments with SCM fits	227
RMSE of SCM fit to t(X) (min), median [IQR]	0.229 [0.134, 0.345]
Family‐out generalization of inferred timescales (SCAM)	
log_10_(*τ*_K_) RMSE, median [IQR]	0.315 [0.259, 0.434]
log_10_(*τ*_K_) MAE, median [IQR]	0.282 [0.243, 0.402]
log_10_(*τ*_K_) empirical coverage of nominal 90% interval, median [IQR]	0.918 [0.886, 0.925]
log_10_(*τ*_K_) empirical coverage of nominal 95% interval, median [IQR]	0.952 [0.941, 0.970]
log_10_(*τ*_D_) RMSE, median [IQR]	0.410 [0.346, 0.529]
log_10_(*τ*_D_) MAE, median [IQR]	0.363 [0.294, 0.452]
log_10_(*τ*_D_) empirical coverage of nominal 90% interval, median [IQR]	0.898 [0.889, 0.910]
log_10_(*τ*_D_) empirical coverage of nominal 95% interval, median [IQR]	0.953 [0.937, 0.972]
Lattice sensitivity analysis	
Experiments included in lattice sensitivity analysis	198
OOF RMSE for log10(*τ*_K_): SCAM vs lattice	0.296 vs 0.317
OOF RMSE for log10(*τ*_D_): SCAM vs lattice	0.430 vs 0.443
Regime agreement rate (OOF)	0.864
Cohen's κ for regime agreement (OOF)	0.835
Regime disagreement fraction on evaluation grid: early / late	0.124 / 0.141

*Note*: SCM RMSE is computed on reconstructed t(X) in minutes. SCAM timescale errors (RMSE/MAE) are calculated in log10(tau/min). Coverage values are empirical fractions of held‐out points falling within nominal predictive intervals under the family‐out protocol.

### 2.4 Composition‐To‐Microstructure Pathways Governing Reduction Rate‐Controlling Timescales

The main‐effect (Figure 3) and interaction maps (Figure 4) show how *τ*_K_ and *τ*_D_ vary with processing conditions (temperature and $p_{H_{2}}$), pellet diameter, and measured pore‐network descriptors (porosity fraction, pore size, and tortuosity). We next ask a more specific mechanistic question about what composition dependence means at the pellet scale. Changes in pellet composition can influence inferred timescales in two distinct ways. First, composition can act directly, i.e., even if two pellets have similar measured pellet architecture descriptors {*D*_pellet_, 𝜀, 𝑑_pore_, τ_tort_}, their timescales can still differ due to differences in reducibility and interface/product‐layer evolution not captured by these descriptors. This residual component is quantified as the natural direct effect (NDE), i.e., the composition effect on log_10_ *τ* at fixed measured architecture. Here, ‘natural’ indicates that the effect is defined using the measured pellet architecture descriptors held at the values they would naturally take under the reference composition, rather than at an externally imposed mediator state. Second, pellet composition can act indirectly by changing the pellet pore network during reduction, so that *τ*_K_ and especially *τ*_D_ shift because the measured transport‐relevant descriptors shift. In our analysis, this indirect pathway is quantified as the natural indirect effect (NIE), defined here as the portion of the composition effect transmitted through {*D*_pellet_, 𝜀, 𝑑_pore_, τ_tort_}, as these mediator values shift from their natural reference composition state to their natural altered composition state. Separating NDE from NIE clarifies whether pellet composition introduces a remaining, architecture‐independent constraint that must be treated as a distinct limitation in reduction behavior, or whether pellet composition mainly affects late‐stage transport by reshaping the pore network (a pathway that can, in principle, be influenced through pellet processing and architecture control).

The pellet composition is represented in two complementary ways to support both conservative domain checks and continuous trend analysis. A six‐domain categorical partition based on basicity and total Fe content is used for composition family holdout and for reporting results by pellet composition family (Table S1). In parallel, a continuous composition coordinate is obtained by principal component analysis (PCA) of the standardized vector [Fe, CaO, MgO, Al_2_O_3_, SiO_2_, TiO_2_], using a fixed sign convention (Figure S6). PC_1_ increases with Fe and decreases with oxide additions, while PC_2_ is dominated by TiO_2_. PC_1_ and PC_2_ together explain 98.9% of the composition variance (Tables S2 and S3). Joint distributions and coupling among variables are documented to delimit the operating and composition envelope (Figures S1–S5).

**FIGURE 5:**
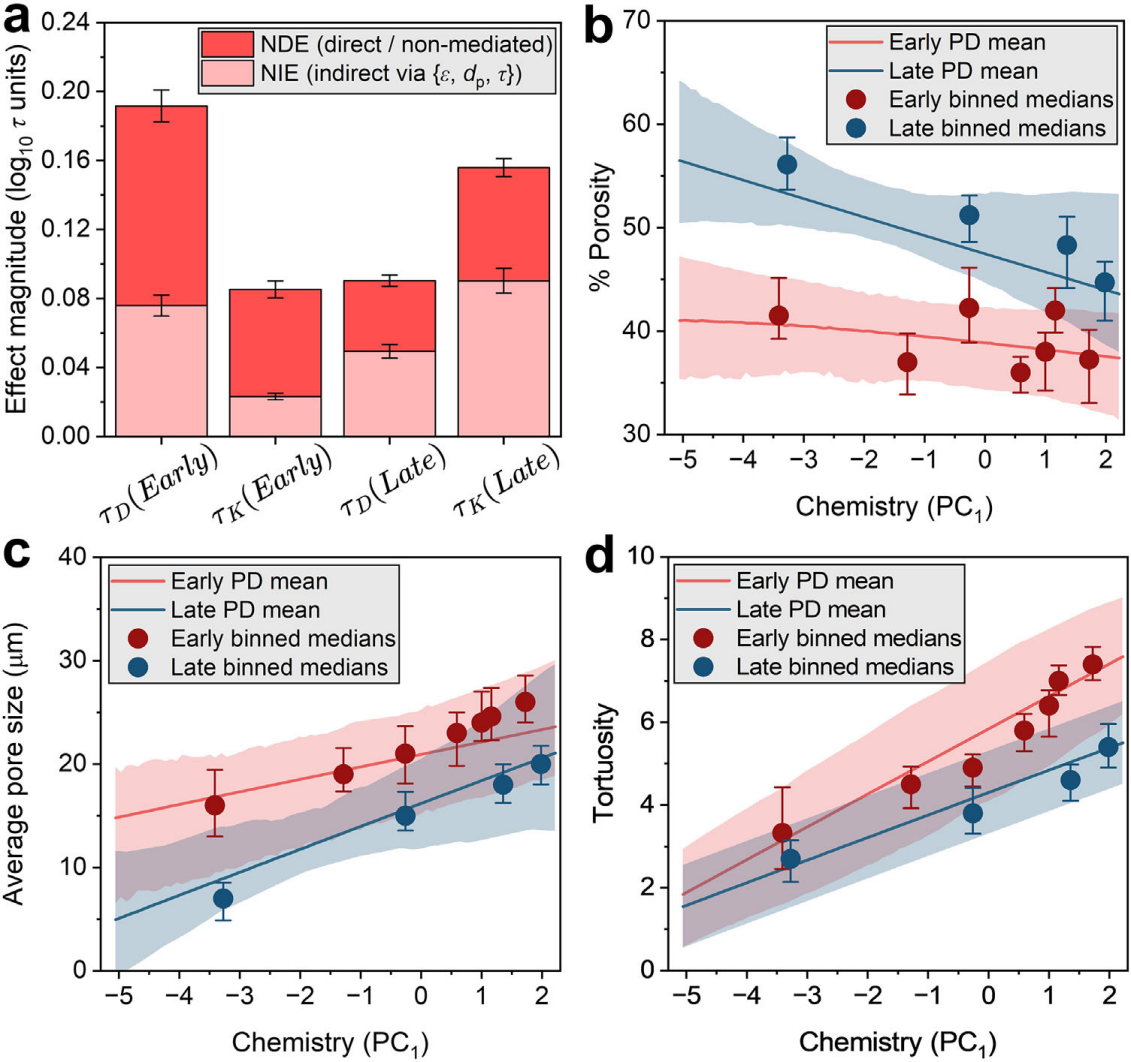
Stage‐dependent pathways depicting the influence of pellet composition on reduction timescales through pellet architecture and intrinsic kinetics. (a) Deconvolution of pellet composition‐driven effects into a direct component (NDE) and an architecture‐mediated component (NIE) evaluated through the architectural descriptors {*ε*/*τ*, *D*_pellet_/*d*_pore_}. Here, ‘natural’ indicates that the decomposition is defined relative to the mediator values that naturally arise under the reference and altered composition states. The partitioning is conversion‐stage specific, indicating that the way pellet composition expresses itself in the kinetics changes as the pore network and product layer evolve. (b–d) Early‐stage and late‐stage architecture dependence on pellet composition is illustrated by conversion‐stage partial dependence (PD) trends for porosity, average pore size, and tortuosity *vs*. the composition coordinate (PC_1_). The separation between early and late trends indicates that pellet composition influences not only interfacial reaction control but also the evolving internal diffusion pathways that govern late‐stage reduction behavior.

To make the composition effect actionable for both researchers and industry, Figure 5 partitions the pellet composition dependence of each timescale into the two components (NDE and NIE) using the transport‐relevant descriptors 𝜀/τ_tort_ and 𝐷_pellet_/𝑑_pore_. We then evaluate how the underlying pellet architecture descriptors 𝜀, 𝑑_pore_, τ_tort_ themselves vary with Chemistry PC_1_, separately for the early and late conversion windows, which anchors the pathway deconvolution to physically interpretable trends in microstructural pore‐network state.

Figure 5a shows that the composition dependence of *τ*_D_ is dominated by the pellet architecture‐mediated pathway, particularly in the late conversion window. This suggests that, within the current dataset, pellet composition primarily influences late‐stage internal diffusion by altering the microstructural state that governs effective diffusion length and connectivity, rather than by introducing a separate, composition‐specific pellet‐scale transport mechanism. The larger mediated contribution at late conversion is consistent with the growing importance of internal diffusion as metallization progresses and the accessible pore network becomes the primary constraint.

In contrast, *τ*_K_ retains a larger residual composition component, most clearly at higher conversion percentages. This indicates that pellet composition influences the effective reaction limitation beyond what is captured by porosity, tortuosity, and mean pore size alone. Plausible sources for this behavior include differences in reducible phase assembly, impurity‐controlled interfacial reactivity, local oxygen potential, and the conversion‐dependent evolution of reactive interfacial area at comparable bulk architecture metrics.

Figure 5b–d illustrate the connection between the early‐/late‐stage partition and measurable structural trends. As Chemistry PC_1_ increases, porosity decreases and tortuosity increases, while the average pore size increases; these trends differ between the early‐ and late‐stage windows. The combination of a lower porosity fraction and larger pores is consistent with pore coarsening and connectivity loss rather than a uniform distribution of percolating porosity, supporting the interpretation that connectivity metrics (number of pores and their spatial distribution), rather than pore size alone, govern late‐stage diffusion rate control. The divergence between the early‐stage and late‐stage windows further shows that a single static architecture descriptor is insufficient to represent the evolving pathway. Taken together, these trends indicate that the framework resolves the stage‐dependent kinetic consequences of structural evolution using available interrupted‐state pellet‐architecture measurements and inferred trajectory‐level timescales, without attempting to reconstruct the full time‐resolved evolution of pore topology, interfacial area, or crack population within the pellet.

Overall, these results provide a mechanistic bridge between pellet composition and late‐stage kinetics. Composition primarily shifts *τ*_D_ by altering the pore network (which dictates the characteristic diffusion length), which can, in principle, be targeted through pellet preparation and thermal history. In contrast, the residual dependence of τ_K_ on pellet composition indicates that interfacial reducibility effects remain important and cannot be eliminated by matching bulk architecture descriptors alone within the stated validity region.

After establishing the overall pellet composition–architectural controls and their coupled behavior, the next question is whether the transport timescale remains governed by a single pellet architecture law across different pellet compositions, or whether changes in pellet composition alter the way pellet architecture variables relate to late‐stage diffusion limitations. We address this by stratifying the transport relations by conservative‐composition domains defined by basicity (CaO/SiO_2_) [52, 53, 54] and total Fe content in the DRI pellets, thereby isolating pellet composition family shifts without introducing additional fitted internal variables.

**FIGURE 6:**
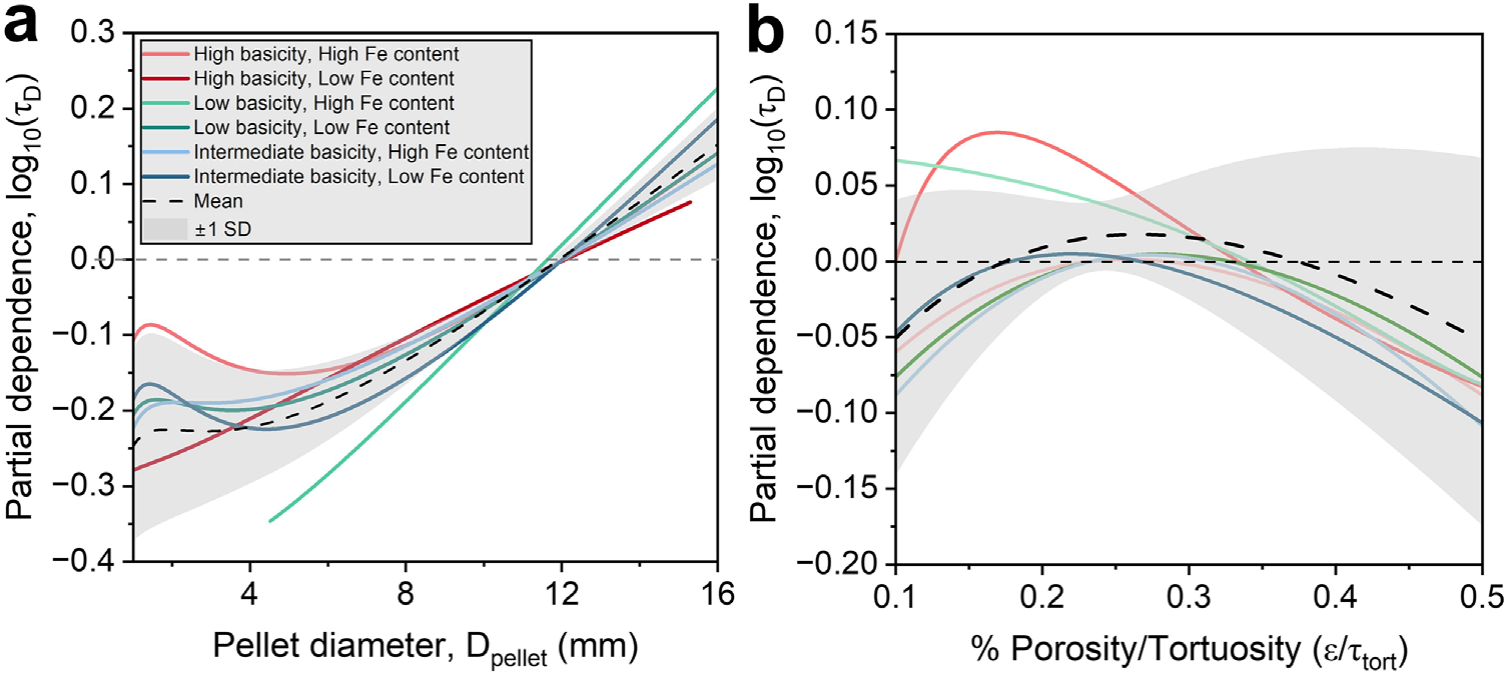
Pellet architecture‐composition coupling in the diffusion‐controlled timescale. (a) Partial dependence of the internal oxygen transport diffusion rate‐controlling timescale log_10_ (*τ*_D_) on pellet diameter *D*_pellet_, shown for composition classes defined by basicity and total Fe content. The diameter scaling is broadly increasing, consistent with a growing average diffusion length scale across the product layer, but the magnitude and curvature depend on pellet composition, indicating that composition‐dependent microstructure evolution modifies the effective rate‐controlling mechanism. (b) Partial dependence of log_10_ (*τ*_D_) on the pellet architecture compactness ratio *ε*/*τ* (porosity divided by tortuosity), again stratified by basicity‐Fe content family classification. Different classes indicate that pellet composition alters how pore connectivity and path tortuosity translate into diffusion rate control, consistent with a conversion‐dependent product‐layer/pore‐network development that is not captured by a single, composition‐invariant transport law. The dashed curve denotes the mean response, and the shaded band indicates the spread across the experimental envelope. Both figures share the same legend given in (a).

Figure 6a shows that the expected increase of *τ*_D_ with pellet diameter is preserved in all pellet composition domains, consistent with diffusion‐length scaling, but the slope and curvature vary across domains. This indicates that the effective internal characteristic transport lengths at high conversion percentages are not invariant with pellet composition, even when porosity, tortuosity, and pore size are included as measured descriptors. A physically plausible reason is the composition dependence of late‐stage phase composition and pore‐network disruption. In high‐basicity and gangue‐rich pellets, the presence of non‐ferrous elements can conceivably impede effective pore network development [53, 54] or form more stable iron‐containing mixed oxide constituents that persist to late conversion stages, resulting in slower kinetics. These accentuate the effective diffusion rate‐limiting effects that extend *τ*_D_ beyond what is predicted by pellet diameter alone.

Figure 6b shows that the decrease of *τ*_D_ with the transport metric *ε*/*τ*_tort_ is monotone in sign but not universal in shape across pellet composition domains. If *ε*/*τ*_tort_ completely parameterized the internal transport state, the relation would be close to a single smooth trend. Instead, the observed curvature implies that similar values of *ε*/*τ*_tort_ can correspond to different pore‐network realizations at the pellet scale. For example, an increase in *ε*/*τ*_tort_ may arise from a better‐connected, homogeneously dispersed pore network that shortens characteristic internal diffusion paths and reduces *τ*_D_. Conversely, local densification, pore coarsening, and heterogeneous redistribution would increase the mean pore size, deteriorate the percolating network, and create slow‐to‐reduce assemblies of unreduced oxide surrounded by extensive metal product, exacerbating transport limitations. Pellet composition controls which pathway is favored by influencing various evolving factors such as sintering kinetics, the stability of gangue‐derived phases, and the mechanical response to reduction‐induced strain, shrinkage, and cracking. The stratified curves, therefore, locate where the transport mapping is composition‐sensitive and where a composition‐invariant relation based only on *ε*, *τ*_tort_, and pore descriptors is insufficient.

To test whether these composition‐dependent couplings prevent transfer across held‐out pellet composition families, Table 1 reports composition‐domain hold‐out performance for the full *τ*‐law model. Here, “full” denotes the reducing conditions and pellet architecture variables augmented by a bounded residual composition correction, while the “physics‐only” form excludes this pellet composition correction. Despite the composition‐dependent relationships shown in Figures 5, 6, the full model maintains low log‐scale errors and calibrated predictive intervals under pellet composition family holdout, indicating that the extracted τ‐laws remain usable for mechanistic mapping across the studied pellet composition envelope. The remaining loss of accuracy is larger for *τ*_D_ than for *τ*_K_, which is consistent with *τ*_D_ being more sensitive to microstructural attributes that are not fully captured by the available pellet‐scale descriptors. The negative‐control results in Figure S23 further show that performance degrades when chemically meaningful coupling information is deliberately perturbed, supporting the idea that the model is using a physically coherent pellet composition–architecture structure rather than exploiting an arbitrary domain label.

Taken together with the main‐effect and interaction results, these results show that temperature and hydrogen potential primarily shift the early‐stage reaction‐limited part of the trajectory, whereas pellet architecture governs the late‐stage transport‐limited tail. Pellet composition primarily influences transport by controlling how pore‐network descriptors translate into effective internal diffusion‐rate limitations at high conversion percentages.

### 2.5 Conversion‐Resolved Reaction–Diffusion Regime Maps

We next convert the SCM timescale deconvolution into rational‐design regime maps that allow us to discern, for a given pellet scale and pore‐network state, at which conversion percentage diffusion becomes the rate‐controlling factor. This cannot be expressed as a single, conversion‐agnostic boundary because the pore network and product morphology evolve continuously with reduction, so the dominant limitation can shift along the same trajectory even when external conditions are fixed. Accordingly, the regime indicator is defined over a conversion window and evaluated as the balance of incremental time accrued by reaction‐controlled and diffusion‐controlled regimes within that window. In physical terms, the map compares the fraction of time spent in a given conversion interval on interfacial reaction versus internal transport. The boundary is therefore a metallurgical statement about “where the time is spent” during the relevant stage of reduction, not a fitted parameter or an assumed global regime. With this definition, the regime maps provide a design‐ and operating‐space view of governing late‐stage kinetics.

We define diffusion‐control dominance from the additive‐time SCM partitioning over a specified conversion window [X_1_, X_2_]. For each rate‐controlling channel *j* ∈ {K, D, M}, the window contribution is Δ*t_j_*  =  τ_*j*_[*g_j_* (*X*_2_) − *g_j_* (*X*_1_)], where 𝑔_𝐾_ and 𝑔_𝐷_ are the standard shrinking‐core basis functions for surface‐reaction and product‐layer transport, and 𝑔_𝑀_ represents the external/access contribution. We then form a dominance ratio Λ = Δ*t_D_*/Δ*t_K_* (or Λ = (Δ*t_D_* + Δ*t_M_*)/Δ*t_K_* when the access term is included), and classify a condition as diffusion‐dominant when Λ>1 for the selected window. This definition is physically meaningful because it compares the actual time increment accrued by each rate‐controlling mechanism over the relevant stage of conversion, rather than comparing cumulative times at an arbitrary endpoint.

**FIGURE 7:**
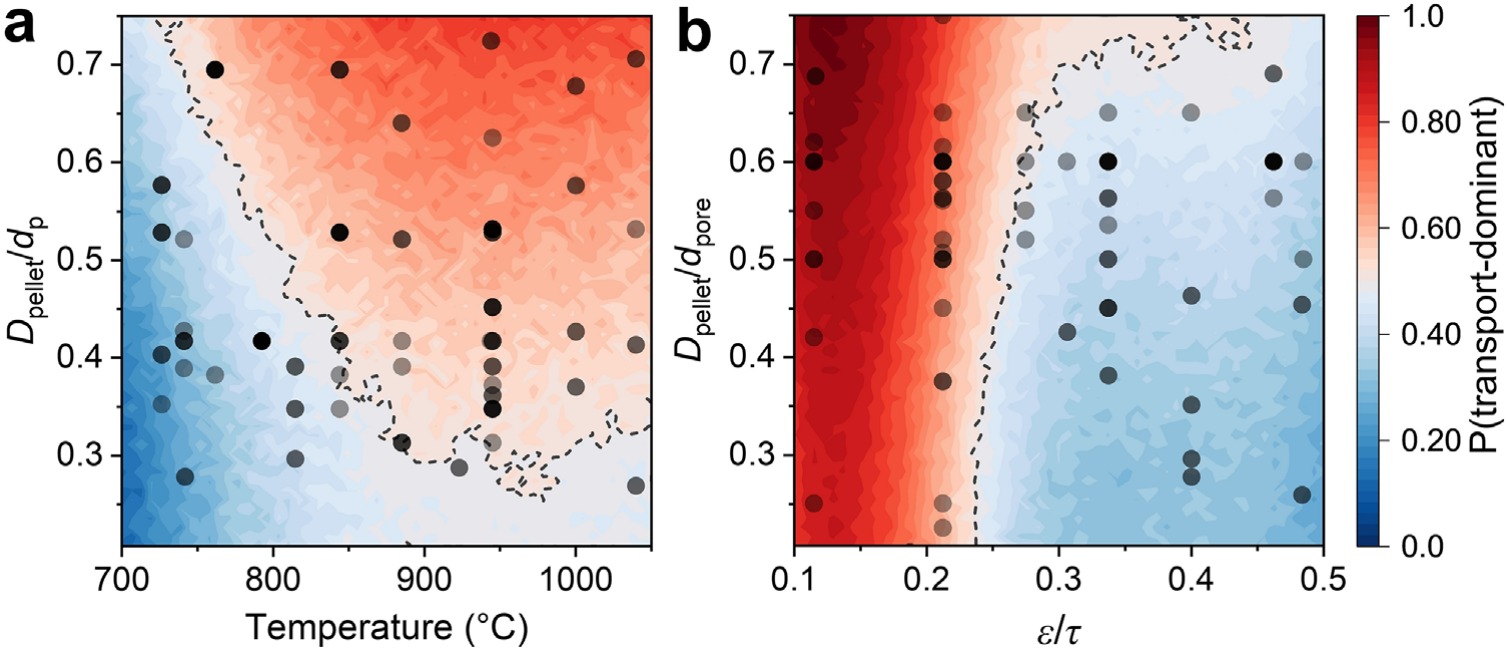
Late‐stage conversion regime maps linking process temperature and pellet architecture to the dominant resistance in hydrogen reduction. Color contours show the transport‐dominant likelihood, expressed as P(*τ*_D_ > *τ*_K_), evaluated at the late conversion stage (X = 0.8), where *τ*_K_ is the trajectory‐inferred interfacial reaction timescale, and *τ*_D_ is the trajectory‐inferred internal transport timescale through the evolving porous/product layer. (a) Map in temperature‐pellet architecture space, sweeping T and *D*_pellet_/*d*_pore_ at fixed *ε*/*τ* = 0.333 (median). (b) Map in effective diffusivity‐architecture space, sweeping *ε*/*τ* and *D*_pellet_/*d*_pore_ at fixed T = 900°C. In both panels, gas conditions are held constant at $p_{H_{2}}$ = 0.8 bar; the architecture ratio is generated by varying *D*_pellet_ = 4–15 mm at fixed *d*_pore_ = 20 µm. The dashed curve marks the equal‐resistance boundary *P*(*τ*_D_ > *τ*_K_) = 0.5, delineating conditions where late‐stage kinetics are more likely to be transport‐influenced (high T, large *D*_pellet_/*d*_pore_, low *ε*/*τ*) from those where reaction resistance remains comparatively more important. Overlaid semi‐transparent circular markers indicate the experimental points from the dataset within the mapped envelope; the shading indicates multiple overlapping experiments.

The color scale in Figure 7 shows the probability *P*(diffusion‐dominant), computed across bootstrap replicates of the full SCM→SCAM inference pipeline, accounting for uncertainty in the inferred *τ* timescales and the activity of each term. The dashed curve is the isoprobability boundary at *P*(diffusion‐dominant) = 0.5, which is best interpreted as an isodominance contour separating regions where diffusion dominance is more likely than not. Regions near the contour are intrinsically less decisive because the underlying uncertainty places non‐trivial mass on both sides of Λ = 1.

With these definitions fixed, the physical interpretation of Figure 7 as a design map is direct. As shown in Figure 7a (temperature versus *D*_pellet_/*d*_pore_), increasing *D*_pellet_/*d*_pore_ shifts the system toward diffusion dominance because it simultaneously increases the characteristic diffusion length scales. Notably, the temperature trend is not interpreted as “high temperature implies reaction control.” Higher temperatures accelerate intrinsic interfacial kinetics, but they also accelerate microstructural processes that can inhibit oxygen removal. The persistence of diffusion‐dominant regions at high temperature for sufficiently large *D*_pellet_/*d*_pore_ is therefore plausible.

Figure 7b collapses the pellet architecture dependence into an effective diffusion metric, *ε*/*τ*_tort_, that serves as a first‐order measure for the effectiveness of the pore network in promoting reduction. The map shows a strong transition from diffusion‐dominant behavior at low *ε*/*τ*_tort_ to reaction‐dominant behavior at higher *ε*/*τ*_tort_, with the boundary shifting systematically with *D*_pellet_/*d*_pore_. Thus, *ε*/*τ*_tort_ governs how efficiently pore characteristics translate into diffusion length scales, while *D*_pellet_/*d*_pore_ sets the geometric limitations for intrinsic size gradients by coupling pellet scale to pore scale. The practical implication is that *ε*/*τ*_tort_ can serve as a “master lever” for adjusting transport sensitivity, but it should be described as an effective descriptor of pore connectivity and characteristic diffusion pathway length rather than a universal constant, because the pore network and product layer thickness itself evolves with temperature and conversion percentage.

The conversion‐dependence of the regime maps is depicted in Figure S24. At X ≈ 0.3, the maps are predominantly reaction‐controlled across most of the envelope, indicating that early conversion is less constrained by oxygen diffusion and predominantly governed by intrinsic reaction kinetics. By X ≈ 0.5, the diffusion‐controlled region expands substantially, particularly at large *D*_pellet_/*d*_pore_ and low *ε*/*τ*_tort_. This shift is mechanistically consistent with reduction‐driven microstructure evolution, even though relatively high temperatures are assumed to favor reaction‐controlled dominance. The regime boundary that moves with the conversion percentage is the signature of a system in which mass transport parameters are not fixed inputs but emergent properties of the evolving mixed‐phase porous solid.

From a pellet design and operating strategy standpoint, the regime maps translate into two robust levers. First, reducing the *D*_pellet_/*d*_pore_ ratio pushes conditions toward reaction control, which can be achieved by reducing pellet diameter, promoting larger, more homogenous pores, or both. Second, increasing *ε*/*τ*_tort_ shifts the system away from diffusion dominance by improving connectivity and shortening effective pathways, which is consistent with designing pore networks that better resist tortuosity inflation and densification as reduction proceeds. The maps also clarify why increasing the temperature alone to achieve a higher reduction rate is an inadequate strategy. Higher temperature can shift *τ*_K_ favorably while simultaneously shifting *τ*_D_ unfavorably through densification, such that the net regime outcome depends on pellet size and architecture. Concisely, these maps provide a mechanistically interpretable guide to where diffusion rate is likely to govern late‐stage reduction efficiency in hydrogen‐based direct reduction of iron oxides.

### 2.6 Reduced‐Order Kinetic Laws for Reaction‐and Diffusion‐Rate Controlled Reduction

The symbolic laws (Table 2) express the inferred conversion regime timescales into compact, mechanism‐consistent constitutive forms. Two aspects are central to the formulations. First, each law is written in terms of dimensionless groups, using fixed reference scales (Table 2), so the expressions are interpretable as kinetic limitations rather than regression artifacts. Second, the physics term is deliberately separated from a small composition correction, introduced only to account for residual pellet composition effects that are not represented fully by temperature, hydrogen partial pressure, and pellet‐architecture descriptors alone. In this sense, the resulting relations can be viewed as effective pellet‐scale constitutive relations for the inferred stage‐resolved timescales. This interpretation differs from forward multiscale additive‐time formulations, in which characteristic timescales are explicitly written for separately resolved reaction and transport contributions within a pellet‐and‐shaft‐furnace description, as shown by Hamadeh, Mirgaux, and Patisson [40]. Here, by contrast, the constitutive relations are written for the inferred *τ*_K_ and *τ*_D_ timescales extracted directly from measured *t*(X) trajectories. A concise correspondence between these inferred timescales and the related mechanistic characteristic‐time relations from Hamadeh et al. is summarized in Table S15.

**TABLE 2:** Final symbolic reduction kinetics laws for the reaction‐ and diffusion‐controlled timescales, log_10_(*τ*_K_) and log_10_(*τ*_D_), respectively. Each law is a two‐stage model comprising a physics‐only symbolic law in dimensionless variables (*T*_ratio_ and gas/geometry groups) plus a pellet composition‐only residual correction (ridge) with quantile‐clipped inputs and an explicit variance cap to limit leverage from rare compositions while preserving the physics relationships. Note that the predictions are valid only within the experimental training envelope/convex hull and must not be extrapolated beyond the measured range.

Item	Subitem	Value
Closure form		log_10_(*τ*) = *f*_phys_(*z*) + Δ_comp_(*c*)
log_10_(*τ*_K_)	*f*_phys_(*z*)	$\frac{2.683{\left(\frac{T_{ref}}{T_{op}+273.15}\right)}^{3}+0.878{\left(\frac{p_{H_{2}}}{P_{ref}}\right)}^{2}}{1.588{\left(\frac{T_{ref}}{T_{op}+273.15}\right)}^{2}+{\left(\frac{p_{H_{2}}}{P_{ref}}\right)}^{3}}$
	Δ_comp_(*c*)	0.95(0.216*B* − 0.008*Fe* + 0.045*G_a_* − 0.047*G_b_*)
	Complete	$\frac{2.683{\left(\frac{T_{ref}}{T_{op}+273.15}\right)}^{3}+0.878{\left(\frac{p_{H_{2}}}{P_{ref}}\right)}^{2}}{1.588{\left(\frac{T_{ref}}{T_{op}+273.15}\right)}^{2}+{\left(\frac{p_{H_{2}}}{P_{ref}}\right)}^{2}}$ + 0.95(0.216*B* − 0.008*Fe* + 0.045*G_a_* − 0.047*G_b_*)
log_10_(*τ*_D_)	*f*_phys_(*z*)	$1.159{\left(\frac{T_{ref}}{T_{op}+273.15}\right)}^{2}+\frac{1.688\frac{D_{pellet}/D_{pellet,ref}}{d_{pore}/d_{pore,ref}}}{\sqrt{\frac{\varepsilon }{\tau_{tort}}}}$
	Δ_comp_(*c*)	0.715(0.932 − 0.215*B* − 0.012*Fe* − 0.02*G_a_* + 0.034*G_b_*)
	Complete	$1.159{\left(\frac{T_{ref}}{T_{gas}+273.15}\right)}^{2}+\frac{1.688\frac{D_{pellet}/D_{pellet,ref}}{d_{pore}/d_{pore,ref}}}{\sqrt{\frac{\varepsilon }{\tau_{tort}}}}$ + 0.715(0.932 − 0.215*B* − 0.012*Fe* − 0.02*G_a_* + 0.034*G_b_*)
Reference scales	*T*_ref_ (K)	1148.15
	*P*_ref_ (bar)	1
	*D*_pellet,ref_ (mm)	12
	*d*_pore,ref_ (µm)	20

The Pareto selection presented in Figure S24 and Table S11 shows that the compact representations for *τ*_K_ and *τ*_D_ are achieved at modest complexity, with the knee solution at complexity 11/14 and loss 0.1076/0.1259, indicating that additional algebraic complexity provides little incremental explanatory gain in the supported domain.

The pellet composition correction Δ_comp_(*c*) in Table 2 should be read as a constrained residual term, not as an alternate rate‐controlling mechanism. It is applied after fixing the physics term, and it is linear in the composition descriptors, including the basicity index B, the Fe content in the pellet, and the pellet composition coordinates for acidic and basic gangue, G_a_ and G_b_. Metallurgically, this term represents the portion of variance that remains after accounting for temperature, hydrogen partial pressure, and pellet architecture, which is associated with composition‐driven differences in phase and microstructure evolution during reduction.

**FIGURE 8:**
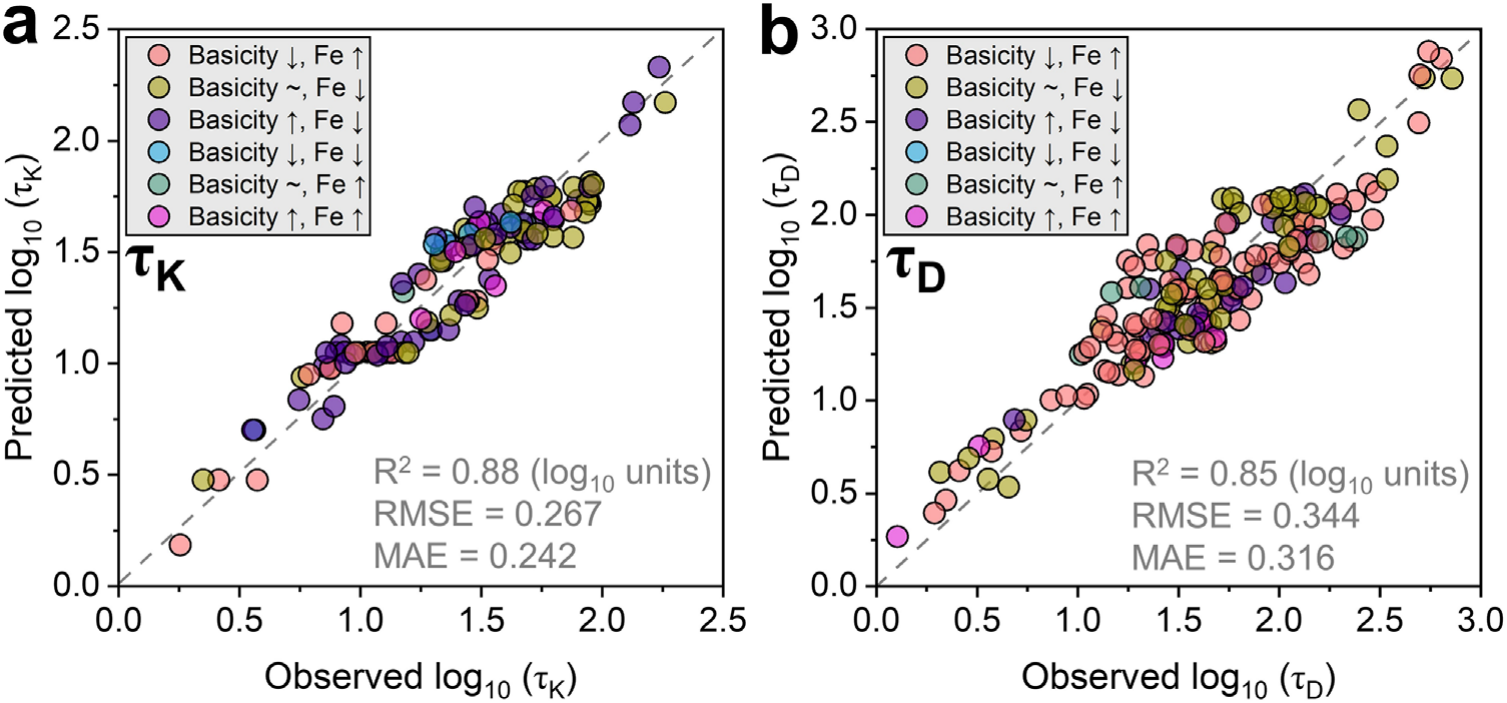
Symbolic‐regression predicted vs. experimentally observed timescales. Parity plots comparing the predicted symbolic regression laws derived log_10_ timescales (y‐axis) against the reference (“observed”) log_10_ timescales (x‐axis) for (a) reaction‐controlled timescale log_10_ (*τ*_K_) and (b) diffusion‐controlled timescale log_10_ (*τ*_D_). Each point corresponds to a unique experiment (colored by pellet composition domain defined by basicity (↓, ∼, ↑) and Fe level (↓/↑)); the dashed line denotes the 1:1 relation. The tight clustering around the parity line across all domains indicates that the symbolic laws capture the dominant systematic variation in *τ*_K_ and *τ*_D_ without strong domain‐specific bias.

The family‐out validation results for the symbolic laws are summarized in Figure 8 and Table S12, which quantify the generalization capability of this separation. When a composition domain is held out, the physics‐only term preserves a reasonable baseline performance, but the residual composition correction consistently reduces error on unseen pellet compositions. For log_10_ (*τ*_K_), the overall family‐out RMSE improves from 0.267 (physics‐only) to 0.217 (total), with similar improvements in MAE (0.242 to 0.206). For log_10_ (*τ*_D_), the improvement is more pronounced within each held‐out composition family; for example, RMSE decreases from 0.413 to 0.311 in the “basicity low, Fe high” hold‐out and from 0.506 to 0.398 in the “basicity intermediate, Fe low” hold‐out, while the overall RMSE improves from 0.344 to 0.291. This pattern is consistent with the role of pellet composition: once the system approaches transport limitation, pellet composition influences *τ*_D_ primarily by controlling how the pore network and product layer evolve, so a physics‐only law based on static pellet architecture descriptors will underpredict cross‐family variation unless a bounded composition correction is included.

## 3 Discussion

Hydrogen‐based direct reduction on the industrial scale is limited in practice by sluggish late‐stage kinetics, which govern furnace operating time, resource expenditure, and recycle‐gas requirements, thereby incurring higher energy and hydrogen costs [6, 7, 10, 12, 13]. The core scientific bottleneck is that the rate‐controlling limitation is not fixed during reduction. The same DRI pellet can initially operate in an interfacial reaction‐controlled regime and later transition to an internal diffusion‐controlled regime as the remaining oxide becomes encapsulated by the growing iron product and the associated pore network degrades. Treating the process with a single regime label or a single fitted parameter set, therefore, obscures the controlling step that matters most for efficient metal production.

This work resolves that ambiguity by making rate control a conversion‐resolved statement anchored to the measured reduction trajectory. The main outcome is not the observation of mixed control, but the ability to place the reaction–transport transition at a defined conversion window (through *τ*_K_ and *τ*_D_ timescales) and to show how that boundary shifts across processing conditions and pellet architecture/composition. This makes specific and testable hypotheses accessible within the compiled experimental reduction data space. For example, at fixed pellet and gas compositions, increasing the pellet‐to‐pore length scale shifts the late stage toward transport limitation, whereas improving pore‐network accessibility delays transport dominance and preferentially shortens the sluggish late‐stage timescale. The regime boundary is therefore not fixed in temperature‐gas space, but evolves along the reduction path.

The classical shrinking‐core and additive‐time descriptions provide the physical basis for the trajectory‐level mixed‐control decomposition adopted in this study. Accordingly, the qualitative dependence of the effective reaction and transport timescales on temperature, gas atmosphere, pellet size, porosity, and tortuosity is consistent with established gas‐solid reduction theory. The present contribution involves resolving these dependencies directly from measured pellet‐reduction trajectories on a conversion‐resolved basis and expressing them as bounded constitutive maps over the sampled multivariable descriptor space.

These constitutive dependence maps, obtained from the SCAM framework, constitute the second practical result of the study. The fact that temperature and gas atmosphere primarily influence the reaction‐controlled timescale, whereas pellet‐scale transport limitations depend more strongly on porosity, tortuosity, and the pellet‐to‐pore length scale, is physically consistent with established mixed‐control theory for porous iron oxide reduction. The main finding of our study in this domain lies in the formulation of these dependencies on a common inferred pellet‐scale timescale basis that resolves the reorganization of controlling sensitivities along the same reduction path. Interaction maps further identify where these sensitivities are coupled, so that changes in one variable modify the sensitivity to another. This resolves multivariable dependencies that are not directly accessible through classical one‐factor‐at‐a‐time kinetic interpretation. These insights can guide pellet design by accounting for variables such as initial porosity and pore‐network morphology, and can also serve as physically meaningful constitutive inputs for reactor‐scale analyses.

The process relevance of these pellet‐scale kinetic outputs is illustrated further in the pellet‐to‐process translation reported in the Section S3. Using the trajectory‐derived quantities *t*(0.90) and [*t*(0.90)–*t*(0.60)]/*t*(0.90), we show that shorter time to high conversion corresponds to higher relative effective hydrogen utilization under a fixed hydrogen feed scenario, while a larger late‐stage time fraction corresponds to a larger auxiliary recycle and conditioning burden under a fixed circulation scenario (Figure S26, Tables S13,S14). These trends make explicit the practical consequences of the late‐stage kinetic tail identified by the SCAM framework, namely that pellet design and operating conditions that suppress the late diffusion‐controlled interval improve hydrogen efficiency and reduce energy demand.

Pellet composition effects are clarified by separating residual direct reducibility effects (NDE) from microstructure‐mediated pathways (NIE). Pellet composition primarily affects the reduction behavior through both intrinsic reducibility and systematic changes in the pore network. Our analyses proceed from this understanding by explicitly decomposing these two pathways on the inferred pellet‐scale timescales, showing that pellet composition predominantly acts through architecture‐mediated transport effects on the transport‐controlled timescale, while a smaller residual direct dependence persists on the reaction‐controlled timescale. This is consistent with composition‐dependent phase assembly and interface evolution, which are not fully captured by bulk pellet architecture metrics. Composition‐family holdout tests further distinguish trends that remain stable across pellet composition families from those that require bounded composition corrections within the sampled envelope.

It is emphasized that the SCAM framework treats reduction‐driven microstructural evolution at the level of its kinetic consequences on the pellet‐scale trajectory. Because τ_K_ and τ_D_ are inferred directly from the measured *t*(*X*) response, these quantities reflect the integrated effect of pore formation, pore coarsening, pore closure, sintering, cracking, and product layer development on the evolving balance between interfacial reaction and internal oxygen transport. The measured pellet architecture descriptors define the experimentally observed structural state at the pellet level (including interrupted‐state measurements across the reduction path), while the conversion‐resolved timescales record how the resulting unresolved internal structural changes alter the rate‐limiting hierarchy along the reduction path. The SCAM framework, however, does not provide explicit time‐dependent evolution laws for pore topology, interfacial area, crack density, or local phase connectivity. Such coupling would require dedicated *in‐situ* or conversion‐resolved structural measurements linked directly to the reduction trajectory.

In addition, the extracted timescales (*τ*_K_ and *τ*_D_) are effective pellet‐scale quantities inferred from conversion trajectories under the adopted mixed‐control formulation. They are not phase‐resolved rate constants, absolute diffusivities, or microstructure‐independent material constants. Accordingly, the present contribution is a conversion‐resolved pellet‐scale kinetic representation in which the effect of structural evolution is carried by the inferred characteristic times and the measured pellet architecture descriptors.

Further, the SCAM framework is validated at the pellet scale within the experimentally sampled descriptor space of the compiled thermogravimetric dataset. Its principal outcome is the conversion‐resolved identification of effective rate‐limiting timescales and their constitutive dependence on operating variables, pellet architecture, and composition within this dataset domain. The composition family holdout tests and uncertainty calibration support transfer across held‐out pellet composition families inside that envelope, but they do not establish quantitative validity outside the observed support. In addition, quantitative extrapolation to pellet diameters larger than 18 mm is not established here, since pellet diameter changes the characteristic internal transport length and can shift the balance between interfacial kinetics and internal diffusion, particularly in the late stages of reduction. Direct transfer to shaft furnace operation is likewise beyond the validated scope of the present analysis, because bed hydrodynamics, inter‐pellet heat and mass transfer, gas recycling, axial gradients, and residence time distributions are not resolved here. The constitutive maps and symbolic laws should therefore be interpreted as experimentally anchored pellet‐scale relations that can guide pellet design and provide physically meaningful constitutive inputs for multiscale reactor models. Their significance lies in revealing how the hierarchy of rate‐limiting resistances reorganizes along the pellet scale reduction trajectory within the experimentally sampled envelope.

In conclusion, we propose practical implications for process and DRI pellet design based on inferences from the SCAM framework. Within the experimentally sampled dataset, the framework identifies variables that accelerate early conversion and those that shorten the late‐stage diffusion‐controlled tail that governs the practical pellet‐scale reduction time. Temperature and reductant gas composition are predicted to exert the strongest influence on the reaction‐controlled timescale at early conversion, whereas pellet architecture variables that govern effective mass transport, including the pellet‐to‐pore length scale and the porosity‐to‐tortuosity ratio, become increasingly decisive as conversion advances to late stages. The regime maps formalize this competition and provide a basis for prioritizing stage‐appropriate interventions rather than uniform conditions throughout the hydrogen‐based direct reduction process. Accordingly, these multivariate insights can guide pellet design and provide experimentally anchored constitutive inputs for multiscale reactor models of hydrogen‐based direct reduction, effectively enabling more sustainable, energy‐efficient green steel production practices.

## 4 Methods

### 4.1 Data Sources, Experimental Variables, and Identifiers

Thermogravimetric mass‐loss trajectories obtained during hydrogen‐based direct reduction of industrial iron oxide pellets were used as the primary input. These were collected from several previously published studies [34, 35, 41, 44, 45, 46, 47], and are summarized in [55]. Each experiment is defined by a unique combination of operating temperature (*T*_op_), total pressure (*P*_gas_), reducing‐gas composition (reported as H_2_ or partial pressures), and pellet descriptors that characterize the geometry and pore architecture. Pellet‐scale descriptors include pellet diameter (*D*_pellet_), porosity (*ε*), tortuosity (*τ*_tort_), and a characteristic pore size *d*_pore_. Pellet composition descriptors are reported at the experiment level and are used only within the validated envelope described in Table S1. In the dataset, these pellet‐architecture descriptors are available as measured values at interrupted reduction states across the experimental set. A reproducible experiment identifier was constructed from the condition set after applying an explicit rounding policy to prevent duplicate or fragmented IDs arising from floating‐point representation (details in Section S1). All downstream fits, resampling, and domain hold‐out tests are grouped by this experiment identifier to respect trajectory‐level dependence.

### 4.2 Conversion Definition and Trajectory Conditioning

Raw mass loss signals *m*(*t*) were converted to a normalized reduction conversion X(*t*) ∈ [0, 1] by referencing the initial mass and the terminal mass plateau of each run after standard baseline handling. The resulting conversion traces were conditioned to enforce physical monotonicity. Specifically, a monotone representation of X(*t*) was obtained by smoothing under an explicit non‐decreasing constraint, which suppresses instrumental noise without introducing non‐physical back‐reaction artifacts. The mechanistic objective used throughout this study is the time‐conversion representation *t*(X), obtained by inverting the X(*t*) mapping and evaluating time at fixed conversion points on a common grid. This transformation preserves the full trajectory information and avoids compressing kinetics into a single scalar, such as an apparent rate constant.

### 4.3 Additive‐time Mixed‐control Representation and Basis Functions

Each experiment was represented using an additive‐time mixed‐control form that partitions the trajectory time into contributions consistent with gas‐film interaction, interfacial chemical reaction, and internal solid‐state or gas transport. This follows the additive reaction‐time perspective for gas‐solid systems [39], expressed here through a shrinking‐core mixed‐control model. The representation is written as:

$$t\;\left(X\right)=\tau_{M}g_{M}\left(X\right)+\tau_{K}g_{K}\left(X\right)+\tau_{D}g_{D}\left(X\right)$$

where *τ*_M_, *τ*_K_, and *τ*_D_ are non‐negative characteristic timescales associated with gas‐film interaction, interfacial reaction, and internal diffusion within the porous pellet, respectively. The conversion‐dependent basis functions 𝑔_𝑖_(𝑋) are fixed, dimensionless functions derived from the standard shrinking‐core mixed‐control model and are evaluated on the same conversion grid as *t*(X). The basis set used in the analysis is:

$$\begin{array}{cc} & g_{M}\;\left(X\right)=X,\;\;\;g_{K}\;\left(X\right)=1-{\left(1-X\right)}^{1/3},\\ & g_{D}\;\left(X\right)=1-3{\left(1-X\right)}^{\frac{2}{3}}+2\left(1-X\right) \end{array}$$

This construction forces mechanistic interpretability at the trajectory level because each term contributes additively to time at a given conversion, while the fitted *τ* values quantify the experiment‐specific magnitude of each contribution.

### 4.4 Non‐negative Timescale Extraction and Stage Resolution

Timescales were extracted by fitting the additive‐time model to the experimental *t*(X) using non‐negative least squares, enforcing *τ*_M_, *τ*_K_, *τ*_D_ ≥ 0 to preserve physical admissibility. A single parameter triplet is insufficient across the full conversion range because the pellet microstructure evolves continuously with conversion progression. To capture this non‐stationary behavior in a controlled manner, the trajectory was partitioned using a fixed split 𝑋_split_ = 0.60. The early stage was defined as *X* ≤ 0.60, and the late stage as *X* > 0.60; this threshold was applied uniformly across all experiments. A continuous two‐stage fit was performed, in which separate early‐stage and late‐stage timescales, $\left\{\tau_{M}^{early},\tau_{K}^{early},\tau_{D}^{early}\right\}$ and $\left\{\tau_{M}^{late},\tau_{K}^{late},\tau_{D}^{late}\right\}$, were extracted while preserving continuity of *t*(X) across the split. The stage‐resolved rate‐control timescales constitute the mechanistic targets for the SCAM‐assisted constitutive mapping step.

### 4.5 Pellet Composition Descriptor Compression by PCA

Pellet composition is used in the framework in two ways. First, it defines domain groupings used for conservative pellet composition family holdout tests. Second, it provides a compact composition coordinate for residual corrections and mediation analysis. To avoid over‐parameterizing pellet composition with many correlated descriptors, a principal component analysis (PCA) was performed on the composition descriptor set after standardization. The first principal component (PC_1_) was retained as the single composition coordinate because it captures the dominant variance in pellet composition within the studied envelope. Figure S6 displays PC_1_ trends in relation to the original pellet composition descriptors. Table S2 presents the PCA loadings, and Table S3 provides the explained variance ratios. Unless explicitly stated, pellet composition is not used to define the primary physics maps for *τ*_K_ and *τ*_D_; it is introduced only where the analysis requires separating direct composition effects from microstructure‐mediated pathways.

### 4.6 Constrained Additive Constitutive Maps for Timescales

The dependence of stage‐resolved timescales on operating conditions and pellet architecture was learned using a constrained additive modeling framework [56], designed to preserve metallurgical admissibility. For a given target *y* ∈ {log_10_ *τ*_K_, log_10_ *τ*_D_}, the model is:

$$y=\beta_{0}+\sum_{j}f_{j}\left(z_{j}\right)+\sum_{\left(j,k\right)\in I}f_{jk}\left(z_{j},z_{k}\right)+\gamma_{stage}+\theta$$

where *z_j_* are physically scaled predictors, *f_j_* are smooth univariate functions, *f_jk_* are selected smooth bivariate interaction surfaces, γ_*stage*_ is a stage indicator term, and θ is the residual. The predictor set is restricted to variables with direct mechanistic meaning at the pellet scale, including inverse temperature *T_ref_*/(*T_gas_* + 273.15), reducing gas potential expressed via $p_{H_{2}}/p_{H_{2}O}$, the geometric group 𝐷_pellet_/*d*_pore_, and the diffusion metric 𝜀/τ_tort_. This choice ensures that learned dependencies correspond to physically interpretable parameters rather than dataset‐specific metrics.

Each *f_j_* is represented using spline bases with a smoothness penalty, and monotonicity constraints are enforced where required by the mechanism. For example, internal diffusion timescales are constrained to increase with increasing 𝐷_pellet_/*d*_pore_ and to decrease with increasing 𝜀/τ_tort_ within the supported domain. Constraints are applied directly at the coefficient level so that the fitted map cannot violate the imposed mechanistic directionality. Implementation details, including basis construction, constraint encoding, and regularization selection, are provided in Section S2.

Because some stage rows correspond to negligible contributions, the SCAM framework uses a two‐part specification. A first model estimates the probability that a given contribution is active under the specified conditions, and a second model estimates log_10_ (*τ*) conditional on activity. This prevents biased fits driven by inactive segments and is essential for regime mapping, where the presence or absence of a contribution is itself conversion dependent. Model fitting and evaluation always respect experiment‐level grouping. Hyperparameters and implementation‐level settings for SCAM are reported in Table S6.

### 4.7 Interaction Surfaces and Their Physical Meaning

Interaction surfaces are introduced to represent non‐separable dependencies that arise when the effect of one variable depends on changes in another variable. An interaction map *f_jk_* (*z_j_*,*z_k_*) indicates that the change in timescale produced by varying 𝑧_𝑗_ is not constant across 𝑧_𝑘_, which is expected when pellet microstructure co‐evolves with conversion, gas composition, and geometry. In hydrogen reduction, representative examples include temperature‐dependent microstructural evolution that modifies the effective transport pathway and conversion‐dependent changes that alter the apparent sensitivity to reducing gas partial pressure. Interaction candidates were screened conservatively and retained when they improved out‐of‐fold performance and remained stable under resampling.

### 4.8 Uncertainty Quantification and Stability Analysis

Uncertainty bands for main effects, interaction surfaces, and derived regime boundaries are obtained through bootstrap resampling at the experiment level, propagating uncertainty throughout the full pipeline from trajectory extraction to constitutive map fitting. Bootstrap replicates refit the additive‐time decomposition and the constrained additive maps, and predictive intervals are computed from the resulting ensemble. Stability tests include permutation‐based negative controls and wrong‐direction monotonicity tests to verify that mechanistic constraints improve, rather than artificially inflate, explanatory power (Figure S24, Tables S9 and S10).

### 4.9 Conversion‐Resolved Regime Mapping

Regime maps summarize the regions where intra‐pellet transport influence becomes dominant relative to interfacial reaction, as a function of operating conditions and pellet architecture, evaluated at a specified conversion window. The regime indicator is constructed from stage‐resolved contributions integrated over the relevant conversion window using the basis increments Δ*g_i_* for that window. For a given window, the integrated contributions are:

$$\;\Delta t_{K}=I_{K}\;\tau_{K}\Delta g_{K},\;\Delta t_{D}=I_{D}\;\tau_{D}\Delta g_{D},\;\Delta t_{M}=I_{M}\;\tau_{M}\Delta g_{M},$$

where $I_{i}$ ∈ {0, 1} are activity indicators sampled from the fitted activity models. A dimensionless dominance ratio is then defined as:

$$\Lambda \;=\frac{\Delta t_{D}+\Delta t_{M}}{\Delta t_{K}},$$

and the transport‐dominant condition is Λ > 1 evaluated over the selected conversion window. The reported map quantity *P*(transport dominant) is the bootstrap‐estimated probability of Λ > 1 conditional on reaction activity, computed over resamples of the full SCM to the SCAM pipeline.

### 4.10 Symbolic Regression for Reduced‐Order Kinetic Laws

Compact reduced‐order laws were derived for log_10_ (*τ*_K_) and log_10_ (*τ*_D_) using symbolic regression restricted to physically scaled variables. The physics‐only candidates set uses $\left\{T_{ref}/\left(T_{gas}+273.15\right),p_{H_{2}}/p_{ref},D_{pellet}/d_{pore},\;\varepsilon /\tau_{tort}\right\}$, with operator sets and admissibility gates chosen to prevent dimensionally inconsistent or non‐physical forms. Candidate equations are evaluated using a multi‐objective criterion that balances predictive loss and symbolic complexity, yielding a Pareto set. The selected equation is the knee point on the gated Pareto front, defined as the candidate with minimum normalized Euclidean distance to the ideal point of minimum loss and minimum complexity.

Family‐out validation is then performed per held‐out pellet composition domain. Two variants are reported. The physics‐only term evaluates the portability of the reduced‐order mechanistic form. The total term includes a residual‐composition correction, applied in the final pipeline to account for chemical‐domain‐specific offsets not captured by the physics variables at the available descriptor resolution. Table S12 reports the RMSE and MAE for both variants on held‐out pellet composition domains, along with the corresponding sample sizes.

## Author Contributions

**A.B**. conceived the project, developed the code, conducted data acquisition, performed model training and analysis, visualized the results, and wrote the initial manuscript draft. **B.R**. assisted in writing the initial manuscript draft. **P.C**. supervised the work and assisted in the review of the manuscript. **D.R**. supervised the work, contributed to conceptualization, and provided critical feedback throughout manuscript development. All authors discussed the results and contributed to the preparation of the final manuscript.

## Conflicts of Interest

The authors declare no conflict of interest.

## Supporting information




**Supporting file**: advs75498‐sup‐0001‐SuppMat.pdf

## Data Availability

The data that support the findings of this study are openly available in Github at https://github.com/bajpaianurag/Hydrogen_Reduction_Reaction_Transition_ML.
