# Supplementary material for: Physics‐Constrained Constitutive Learning of Rate‐Limiting Timescales for Efficient Hydrogen‐Based Direct Reduction for Green Steel Making
Source: Adv Sci (Weinh). 2026 May 4:e75498. Online ahead of print. doi: 10.1002/advs.75498 (PMC13335568; doi:10.1002/advs.75498)
Supplement: Supplementary file 1 — Supporting file: advs75498‐sup‐0001‐SuppMat.pdf [file ADVS-9999-e75498-s001.pdf]

Supplementary Information

Physics-constrained Constitutive Learning of rate-limiting timescales for efficient Hydrogen-based Direct Reduction for Green Steel Making

Anurag Bajpai[1*], Barak Ratzker[1*], Pasquale Cavaliere[2,3], Dierk Raabe[1*]

[1]Max Planck Institute for Sustainable Materials, 40237 Düsseldorf, Germany

[2]Department of Innovation Engineering, University of Salento, Via per Arnesano, 73100 Lecce, Italy

[3]Process Metallurgy Research Unit, Faculty of Technology, University of Oulu, 90570 Oulu, Finland

This PDF file contains:

1. Supplementary Methods Sections: S1 to S3
2. Supplementary Figures: S1 to S26
3. Supplementary Tables: S1 to S15

S1. Experiment identifiers and rounding policy (trajectory-level grouping)

Each thermogravimetric experiment produces a full trajectory X(t) and must be treated as the statistical unit for all resampling, cross-validation, and domain holdout protocols. A deterministic experiment identifier was therefore constructed from the experimental condition set using an explicit rounding policy. The objective is to (*i*) prevent spurious splitting of a single experiment into multiple IDs due to floating-point representation or file-format conversions, and (*ii*) avoid accidental merging of genuinely distinct experiments by using a precision consistent with the effective measurement resolution.

S1.1 Canonical feature set used to define an experiment

The identifier is derived from the subset of variables that uniquely define the imposed experimental conditions and the as-prepared pellet state for a run. The canonical fields are:

- Pellet and bulk state: D_{pellet} (pellet diameter), ρ (pellet density)
- Thermal conditions and reductant gas: T_{gas}, P_{gas}, reducing-gas partial pressure (pH_2)
- Composition (wt.% basis): Fe, CaO, MgO, Al_2O_3, SiO_2, TiO_2 (and other fixed composition descriptors used in the dataset manifest)

Variables that vary along the trajectory (conversion, time, or derived stage fields) are never used in the experiment key.

S1.2 Rounding policy

All numeric fields are rounded to a fixed number of decimals before string encoding. The rounding precisions are selected to be conservative relative to (a) the experimental control/recording resolution and (b) typical reporting precision in lab logs and spreadsheets. The policy used in this work is summarized below.

Field	Unit	Rounding precision	Rationale
D_{pellet}	mm	0 decimals	Pellet diameter recorded at integer mm resolution in the dataset
ρ	g·cm^{-3}	0 decimals	Density level treated as an integer-like bin in the dataset
T_{gas}	°C	0 decimals	Temperature controlled/reported at ~1 °C resolution
P_{gas}	bar	3 decimals	Pressure commonly logged with 10^{-3} resolution in the dataset
pH_2	fraction (or vol%)	3 decimals	Composition precision limited by MFC setpoints/reporting
Fe	wt.%	4 decimals	Composition stored at 10^{-4} wt.% resolution in the processed table
CaO	wt.%	4 decimals	Same as above
MgO	wt.%	4 decimals	Same as above

This rounding is applied only for identifier construction. All modeling uses unrounded numeric values (after standard preprocessing), so that the rounding policy does not alter the physics maps or regression fits.

S1.3 Canonicalization and deterministic encoding

Before rounding and string formation, each field is coerced to a numeric and standardized to a consistent unit convention (e.g., H_2 to a fraction; pressures to a consistent unit if multiple units exist in raw files). Missing fields are handled explicitly. If a canonical field is missing for an experiment, it is encoded as the literal token NA (not as an empty string), so that missingness does not silently collapse distinct experiments. The rounded values are then serialized into a fixed-order key–value string to guarantee determinism across platforms and file reads.

A representative human-readable signature (versioned) is:

$$\text{sig\_v1} = \text{D} = \langle \text{D} \rangle|\text{rho} = \langle \rho \rangle|\text{T} = \langle \text{T} \rangle|\text{P} = \langle \text{P} \rangle|\text{yH2} = \langle \text{y} \rangle|\text{TFe} = \langle \text{TFe} \rangle|\text{CaO} = \langle \text{CaO} \rangle|\text{MgO} = \langle \text{MgO} \rangle.$$

The final experiment identifier used in all tables is stored as:

- a readable signature (for audit), and
- a compact hash (for robust uniqueness in filenames and merges).

S1.4 Hashing and versioning

To make identifiers robust to long strings and reduce merge errors, the signature is hashed using a stable cryptographic hash (SHA-1) and truncated to a fixed length (12 hex characters). The experiment identifier is then:

$$\text{experiment\_id} = \text{exp\_v1\_}\langle \text{hash(sig\_v1)} \rangle.$$

The explicit v1 tag encodes the rounding and field list version. If the canonical fields or rounding precisions change in future revisions, the version tag is incremented to prevent silent incompatibility between tables created under different policies.

S1.1.5 Collision and consistency checks

After ID creation, two integrity checks are executed:

1. Collision check: ensure that experiment_id is unique across distinct sig_v1. If a collision occurs, the hash length is increased.
2. Within-ID invariance check: for each experiment_id, verify that the underlying canonical fields are constant across all rows belonging to that experiment (e.g., across time points). Any deviation flags a data-ingestion error or an unaccounted condition change.

S2. Shape-constrained additive spline models for constitutive timescale maps

This section defines the spline basis, the coefficient-level constraint encoding, and the regularized fitting procedure used to obtain auditable constitutive maps for the extracted timescales. The goal is to learn smooth, physically consistent dependencies without allowing the fitted map to violate known mechanistic directionality.

S2.1 Model form and targets

For each extracted timescale target y ($\log_{10}(\tau_K)$ or $\log_{10}(\tau_D)$), we represent the constitutive law as an additive sum of one-dimensional smooth components,

$$y = \beta_0 + \sum_j f_j(z_j) + \theta$$

where z_j are standardized, physically scaled predictors and β_0 is an intercept. Each component f_j is a spline function whose shape is controlled by an explicit constraint type. This makes the learned dependence directly interpretable as a set of main-effect curves (and, when used, interaction surfaces are handled analogously through tensor-product spline bases).

S2.2 Predictor scaling and support control

All continuous predictors are first expressed in physically disciplined forms (examples used in this work include $T_{ref}/(T_{gas} + 273.15)$, $\log p_{H_2}$, $\log D$, $\log(\varepsilon/\tau_{\mathrm{tort}})$, $\log d_{\mathrm{pore}}$, and a stage conversion coordinate when stage-resolved fits are used). Each predictor is then standardized using training statistics,

$$z_j = \frac{x_j - \mu_j}{\sigma_j},$$

with μ_j, σ_j computed on the training set used for fitting. To prevent extrapolative spline behavior outside the observed envelope, standardized values are clipped during model evaluation to the training support,

$$z_j \leftarrow \min\{\max\{z_j, z_{j,min}\}, z_{j,max}\}.$$

The support limits $(z_{j,min}, z_{j,max})$ are stored for reproducible inference. This support control prevents uncontrolled spline excursions outside the training envelope, but it does not constitute quantitative validation for extrapolation beyond the observed pellet scale descriptor space.

S2.3 B-spline basis construction

Each component $f_j(z)$ is expanded in a cubic B-spline basis. Let the spline degree be $k = 3$. A knot vector t is constructed from the empirical distribution of the training z values using a quantile rule. Specifically, for a requested basis size controlled by df, the internal knots are selected from evenly spaced quantiles of the training data. End knots are repeated k+1 times to

form an open knot vector. With this construction, the basis functions $B_m(z)$ are evaluated using the standard B-spline recursion, and the component is written as

$$f_j(z) = \sum_{m=1}^{M_j} B_{jm}(z)\,\vartheta_{jm}$$

For numerical identifiability and interpretability, basis columns are mean-centered on the training data so that each smooth term contributes as a deviation around zero,

$$B_{jm}(z) \leftarrow B_{jm}(z) - \frac{1}{n}\sum_{i=1}^{n} B_{jm}(z_i).$$

S2.4 Coefficient-level monotonicity constraints via integrated bases

Monotonicity is enforced through bound constraints on coefficients, not through *post hoc* filtering. For monotone components, we do not use the raw spline basis directly. Instead, we construct an integrated basis $B_{jm}^{\mathrm{int}}(z)$ defined as the cumulative integral of the corresponding nonnegative B-spline basis over the supported domain,

$$B_{jm}^{\mathrm{int}}(z) = \int_{z_{min}}^{z} B_{jm}(u)du,$$

computed numerically on a fixed grid and interpolated to the query z. A monotone increasing component is parameterized as

$$f_j(z) = \sum_{m=1}^{M_j} B_{jm}^{\mathrm{int}}(z)c_{jm}, \qquad c_{jm} \geq 0$$

Since $\frac{d}{dz}f_j(z) = \sum_m B_{jm}(z)c_{jm}$ and $B_{jm}(z) \geq 0$, the nonnegativity constraint $c_{jm} \geq 0$ guarantees $f_j'(z) \geq 0$ over the full support. Monotone decreasing components use the sign-reversed parameterization,

$$f_j(z) = -\sum_{m=1}^{M_j} B_{jm}^{\mathrm{int}}(z)c_{jm}, \quad c_{jm} \geq 0$$

which guarantees $f_j'(z) \leq 0$. These correspond to the constraint kinds implemented in the workflow as

monotonic increasing and monotonic decreasing. Unconstrained smooth terms use the raw B-spline basis.

Mechanistic directions are specified a priori per predictor. For example, transport timescales are constrained to increase with pellet size and tortuosity and to decrease with $\varepsilon/\tau_{\mathrm{tort}}$ and pore size, while reaction timescales are constrained to increase with $T_{ref}/(T_{gas} + 273.15)$ and decrease with hydrogen partial pressure when those directions are physically warranted for the chosen variable definitions.

S2.5 Penalized constrained least squares and smoothness control

All spline coefficients are estimated by minimizing a penalized least-squares objective subject to bound constraints,

$$min_{\beta}\|y - A\beta\|_2^2 + \lambda\beta^{\top}P\beta \qquad \text{such that} \qquad \ell \leq \beta \leq u.$$

Here A is the full design matrix assembled by concatenating each feature block, β collects the intercept and all spline coefficients, and (ℓ, u) encodes coefficient bounds. For monotone terms,

$\ell = 0$ and $u = +\infty$ are applied to the integrated-basis coefficients c_{jm}. Intercept terms are left unbounded.

Smoothness is enforced through a discrete difference penalty applied within each spline block. Let D be the order-r finite-difference operator on coefficients (with $r = 2$ used here). The penalty is

$$P = D^{\top} D,$$

assembled in block-diagonal form across all spline components so that smoothness is controlled within each f_j independently. The constrained optimization is solved as a bound-constrained, Tikhonov-regularized least-squares problem using an augmented system,

$$min_{\beta} \left\| \begin{bmatrix} A \\ \sqrt{\lambda} L \end{bmatrix} \beta - \begin{bmatrix} y \\ 0 \end{bmatrix} \right\|_2^2 \qquad \text{such that} \qquad \ell \leq \beta \leq u,$$

where L is a square-root factor of P. In the implementation, L is taken directly from the differencing operator to produce the correct $D^{\top}D$ penalty. The solver used is a bound-constrained least-squares routine, which guarantees that monotonicity constraints are satisfied exactly.

S2.6 Regularization selection with grouped cross-validation

The smoothness weight λ is selected separately for each target using grouped cross-validation to avoid leakage across conversion windows from the same experiment. A deterministic experiment-level fold assignment is constructed from experiment_id with a fixed random seed, ensuring that all rows associated with a given experiment are assigned to the same fold. For each candidate λ on a log-spaced grid, the model is refit on $K-1$ folds and evaluated on the held-out fold using RMSE on y. The selected λ^* minimizes the mean RMSE across folds. Cross-validation curves are saved as tables for auditability and reproducibility.

S3. Illustrative pellet scale process translation under fixed operating assumptions

To make the relevance of the pellet-scale kinetic outputs more explicit, an illustrative process translation was performed using the conversion-resolved trajectory representation established in this study. This analysis is introduced solely as a secondary interpretive layer and is not a reactor-scale model. Its purpose is to quantify how the pellet scale time required to reach a practically high reduction level, and specifically the late stage kinetic penalty, would influence hydrogen exposure and auxiliary circulation burden under clearly stated fixed operating assumptions.

The translation was anchored at a target conversion of X=0.90. This choice was adopted because it lies within the validated fitting range of the present trajectory analysis and remains sufficiently close to the late stage region where the practical time penalty becomes most relevant. For each experiment, the processed t(X) representation was used to extract t(0.60) and t(0.90). From these quantities, two pellet scale descriptors were then computed.

The first was the late stage time penalty,

$$\Delta t_{late} = t(0.90) - t(0.60).$$

The second was the late-stage fraction,

$$f_{late} = \frac{[t(0.90) - t(0.60)]}{t(0.90)}.$$

These two quantities characterize, respectively, the absolute and relative burdens of the sluggish high-conversion interval within the total time required to reach X=0.90.

To provide an explicit, yet controlled, process-level interpretation, the analysis was formulated under a fixed hydrogen feed and auxiliary recycle scaling scenario. The median t(0.90) across the experimental set was taken as the reference pellet-scale condition, denoted t_r. The chemically required hydrogen for the chosen target conversion was evaluated based on the stoichiometric requirement for complete hematite-to-iron reduction, using 54 kg H_2 per ton Fe for full conversion. At X = 0.90, this yields 48.6 kg H_2 per ton Fe on a chemically required basis.

For the illustrative translation, the total hydrogen supply was anchored in the industrial range, namely 90 to 130 kg H_2 per ton DRI, and the midpoint, 110 kg H_2 per ton DRI, was adopted as the reference value. The auxiliary recycle and gas conditioning electricity was anchored to the range cited in the main manuscript, namely 0.56 to 0.59 MWh per ton Fe feed, and the midpoint value, 0.575 MWh per ton Fe feed, was adopted as the reference value. Under these fixed operating assumptions, the supplied hydrogen was scaled in proportion to $t(0.90)/t_r$. The corresponding relative effective hydrogen utilization metric was then defined with respect to the median reference case. In the same manner, the auxiliary recycle and conditioning burden was also assumed to scale with $t(0.90)/t_r$, so that slower pellet scale reduction under otherwise fixed conditions translated to a higher auxiliary energy penalty.

These quantities are presented only as illustrative process proxies. They are not direct predictions of the shaft furnace hydrogen utilization factor or total plant energy consumption, because the current pellet-scale dataset does not resolve bed hydrodynamics, gas recycle, purge policy, residence time distribution, or reactor-scale thermal fields. The aim of the analysis is therefore not to replace reactor modelling, but to translate the pellet-scale kinetic outputs resolved by the SCAM framework into explicit, process-relevant indicators under fixed operating assumptions.

The resulting trends are shown in Figure S26 and summarized numerically in Tables S13 and S14. Figure S26a visualizes the imposed process translation under the fixed hydrogen feed scenario, using the pellet scale time to X=0.90 as the governing kinetic variable. It shows that a shorter time to reach X=0.90 corresponds to higher relative effective hydrogen utilization under the fixed-hydrogen-feed assumption. This relation follows directly from the scenario definition and makes explicit the process consequence of faster pellet scale reduction. Figure S26b shows that a larger late-stage time fraction is associated with a larger auxiliary recycle and conditioning burden under the fixed circulation assumption. This second relation is particularly important because it links the process penalty directly to the late-stage kinetic tail, central to the mechanistic interpretation developed in the study.

The quantile summary in Table S13 demonstrates that this process translation is systematic across the compiled pellet scale dataset. From the 10th to the 90th percentile, t(0.90) increases from 10.551 to 60.013 min, while the late stage penalty increases from 5.295 to 32.866 min, and the late stage fraction increases from 0.406 to 0.655. Over the same interval, the relative effective hydrogen utilization decreases from 1.967 to 0.359, whereas the illustrative auxiliary recycle and conditioning energy increases from 0.282 to 1.602 MWh t^{-1} Fe, corresponding to a relative auxiliary burden increase from 0.490 to 2.786. These values make explicit that the pellet-scale kinetic tail, once translated under fixed operating assumptions, imposes a direct process penalty on both hydrogen efficiency and auxiliary energy demand.

The representative cases collected in Table S14 provide a concrete illustration of the same effect. The lower-reduction-time case reaches X=0.90 in 5.001 min and shows a late-stage fraction of 0.393, a relative effective hydrogen utilization of 2.156, and a relative auxiliary burden of 0.232. By contrast, the higher reduction time case requires 200.004 min to reach the same target conversion, with a late stage fraction of 0.654, a relative effective hydrogen utilization of 0.109, and a relative auxiliary burden of 2.676. The median reference case lies between these two limits. These case-level contrasts are presented only to illustrate magnitude. The quantile summary remains the primary numerical description because it is less sensitive to isolated trajectories and better represents the overall dataset behavior.

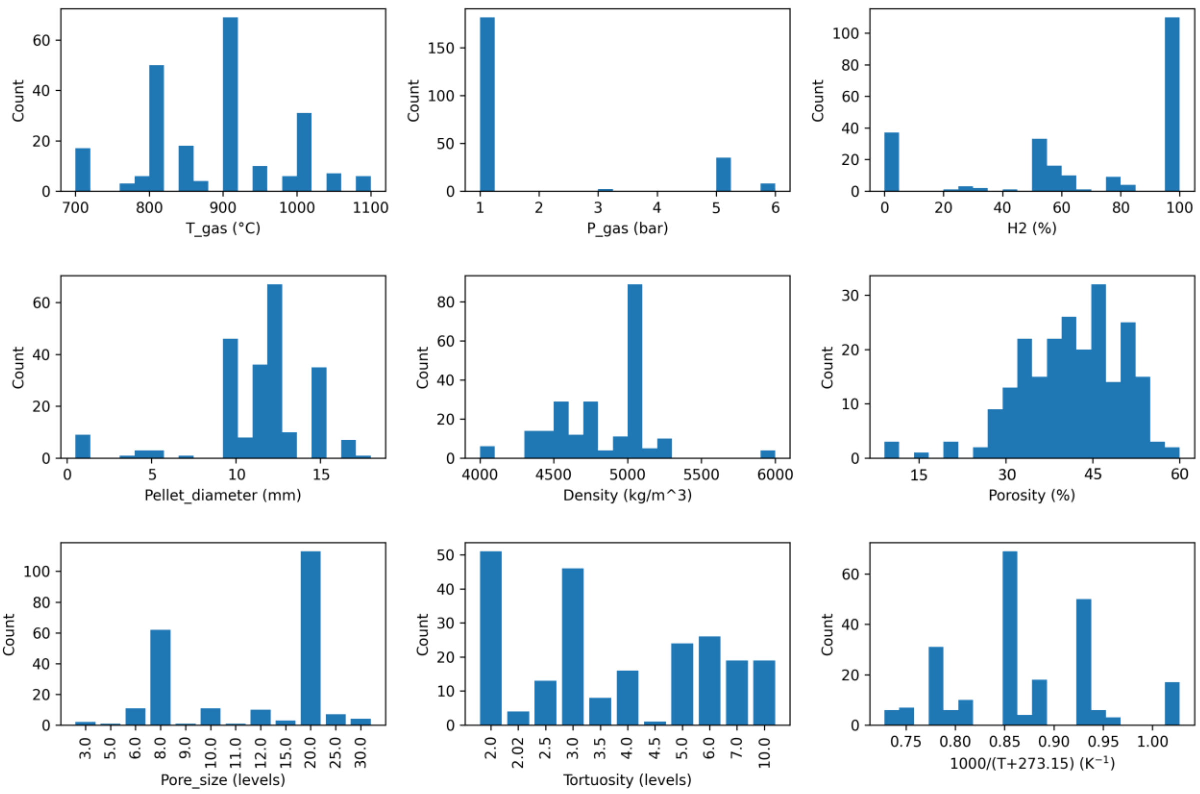

Figure S1 – Histograms summarizing the distribution of gas conditions and pellet-architecture descriptors across all experiments, including T (°C), total gas pressure P_{gas} (bar), hydrogen fraction H_2 (%), pellet diameter (mm), green density (kg·m^{-3}), and porosity (%). The pore-size and tortuosity descriptors are recorded as discrete levels (reflecting the available characterization/assignment scheme), and the temperature is additionally shown in Arrhenius form as 1000/(T+273.15) (K^{-1}) to align with the reaction timescale parameterization.

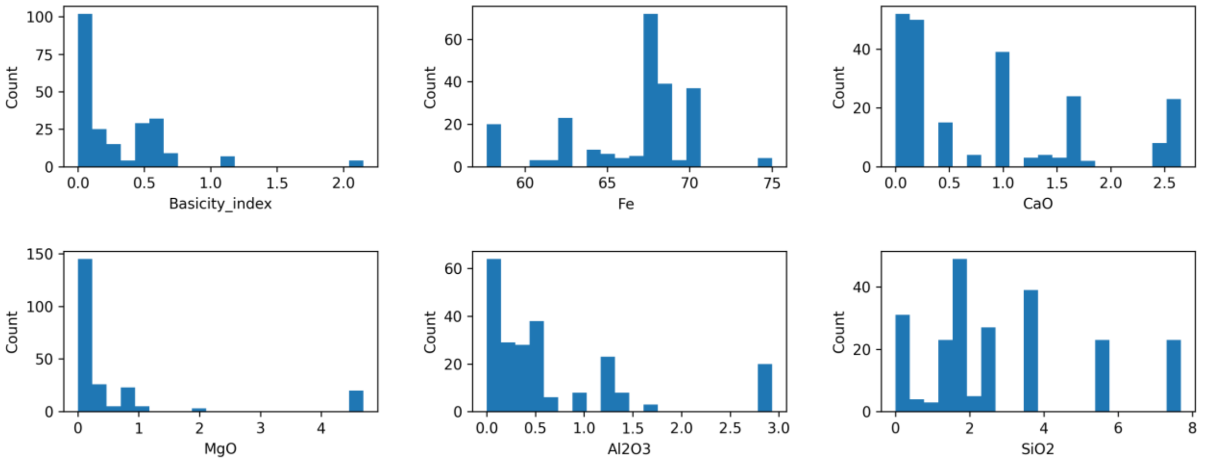

Figure S2 – Histograms show the spread of bulk composition variables across the dataset: basicity index (defined in the main text, e.g., B=CaO/SiO_2), total iron content (Fe), and major oxide fractions (CaO, MgO, Al_2O_3, SiO_2; consistent with the dataset's reported basis). The distinctly clustered compositions reflect the discrete feed/ore families, as well as the flux additions, sampled in this study.

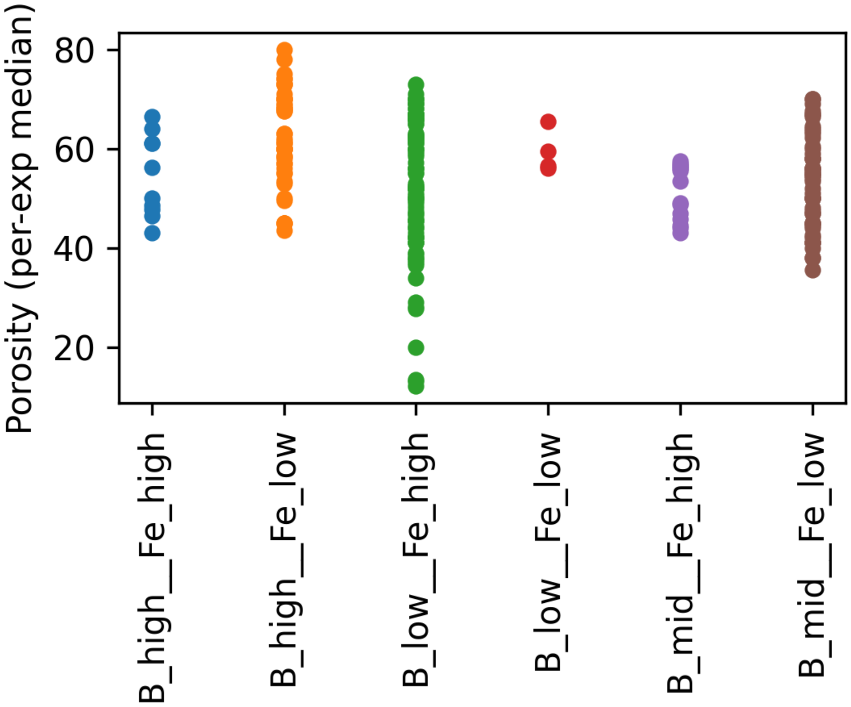

Figure S3 – Per-experiment median porosity is shown for the discrete composition group labels formed by binning basicity (B: high/low/intermediate) and total iron content (Fe: high/low), yielding strata such as B high-Fe high, B high-Fe low, etc. Each point corresponds to a single experiment, highlighting the experiment-level architectural state employed in the framework. The separation and spread of porosity across these strata demonstrate that the composition groupings are not cosmetic: they align with systematic, measurable differences in pellet architecture, supporting the physical plausibility of composition influencing transport resistance indirectly through architecture descriptors (e.g., porosity/tortuosity and related ε/τ, $D_{\mathrm{pellet}}/d_{\mathrm{pore}}$) in the staged mediation analysis.

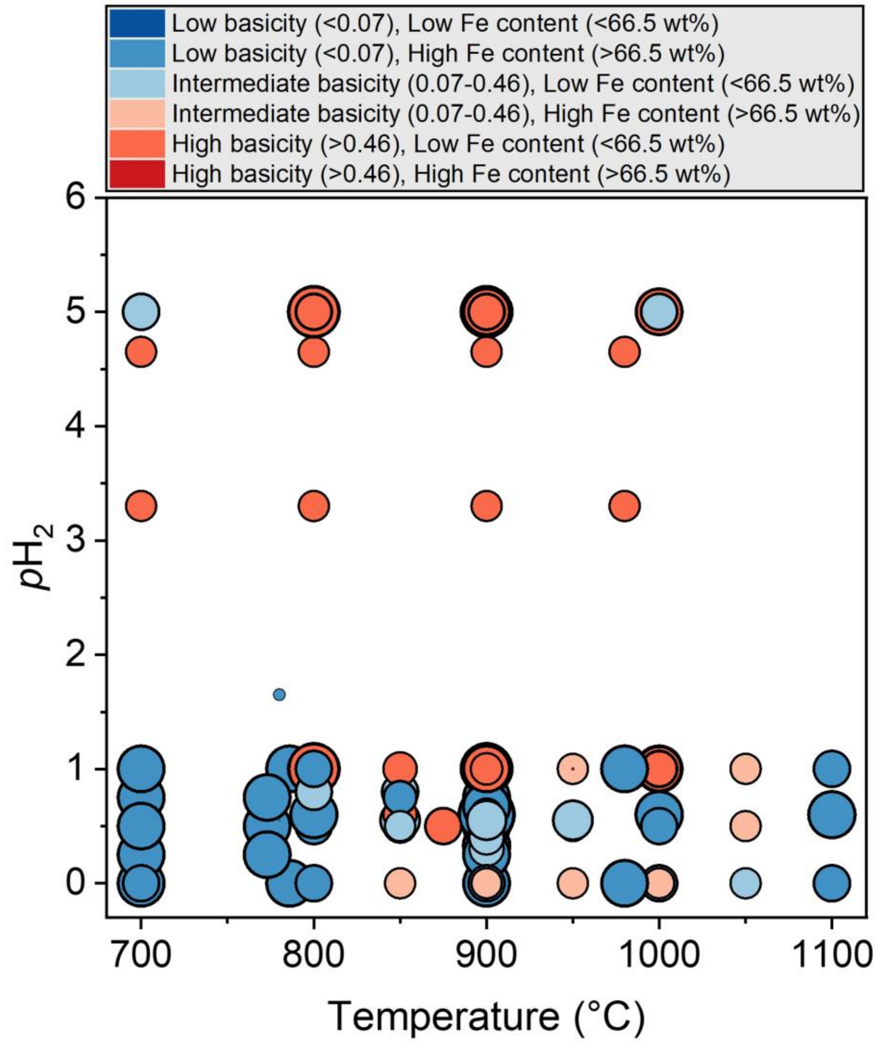

Figure S4 – Scatter plot of the whole experimental data matrix in temperature and hydrogen partial pressure (pH_2). Point color denotes the combined composition strata defined by basicity and total Fe content (thresholds as indicated in the legend), illustrating how composition domains populate the operating space. Point size scales with pellet porosity (%), highlighting the co-variation between operating reducing conditions and initial pellet architecture, which motivates conditioning and negative-control checks in the mechanistically constrained analysis.

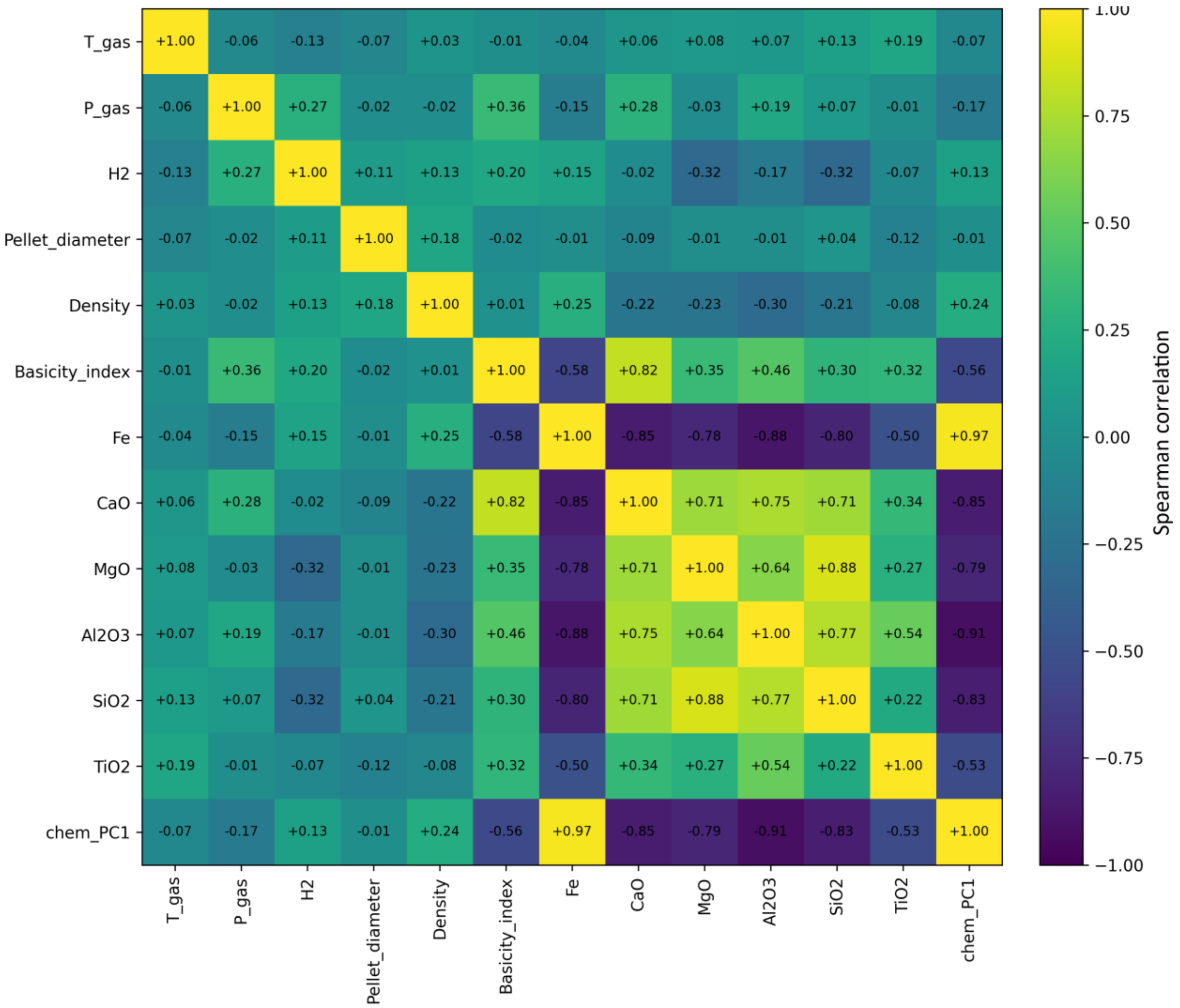

Figure S5 – Spearman rank correlation matrix for the key experimental variables (gas temperature T_{gas}, total pressure P_{gas}, H_2 fraction), pellet descriptors (diameter, density), and composition terms (basicity index and major oxide constituents), together with the first composition principal component (composition PC_1). Cell values report the Spearman coefficient ρ, providing a nonparametric view of monotone co-variation and potential collinearity within the experimental envelope that the mechanistically constrained modelling must accommodate.

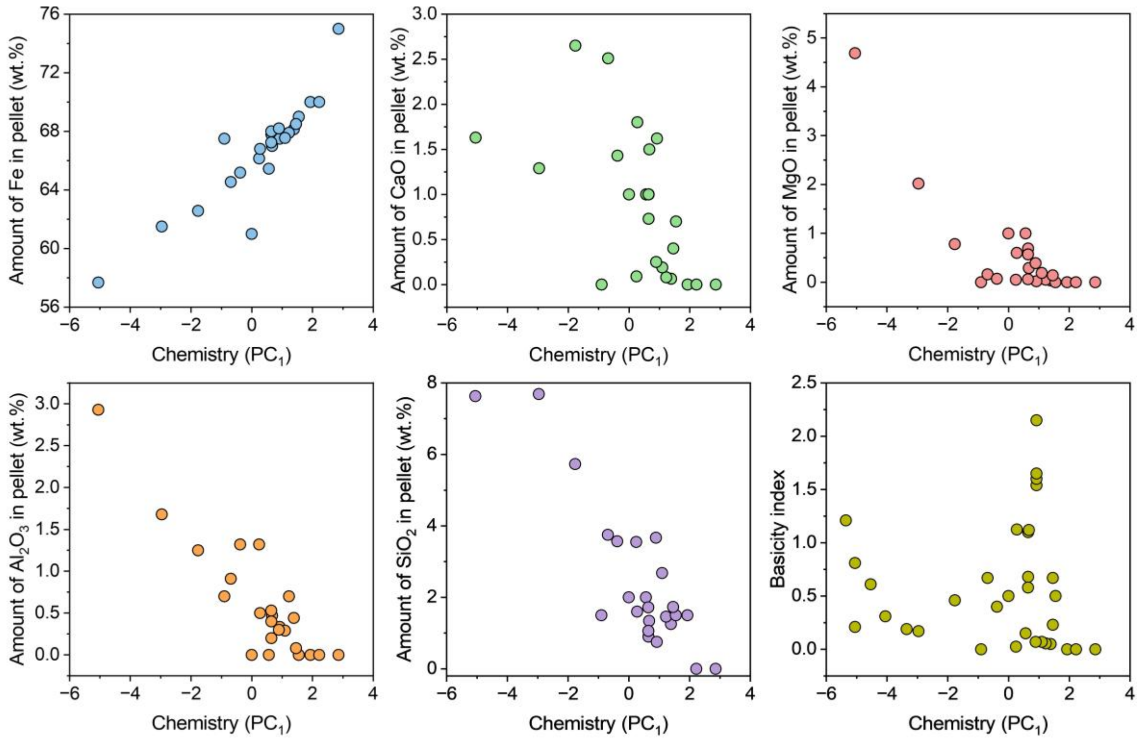

Figure S6 – Pairwise scatter plots relating the first composition principal component (PC_1) to the measured pellet composition descriptors (Fe and major gangue oxides: CaO, MgO, Al_2O_3, SiO_2) and to the basicity index. Across the dataset, PC_1 increases with iron content (Fe) and decreases with the gangue-oxide burden (CaO, MgO, Al_2O_3, SiO_2), indicating that PC_1 serves as a compact axis that orders pellets from Fe-rich/low-gangue to lower-Fe/higher-gangue compositions. This descriptor is used in the main text as a composition coordinate for assessing how compositional variability modulates reduction kinetics and microstructure-linked transport rate-control, without implying a causal direction beyond the observed co-variation.

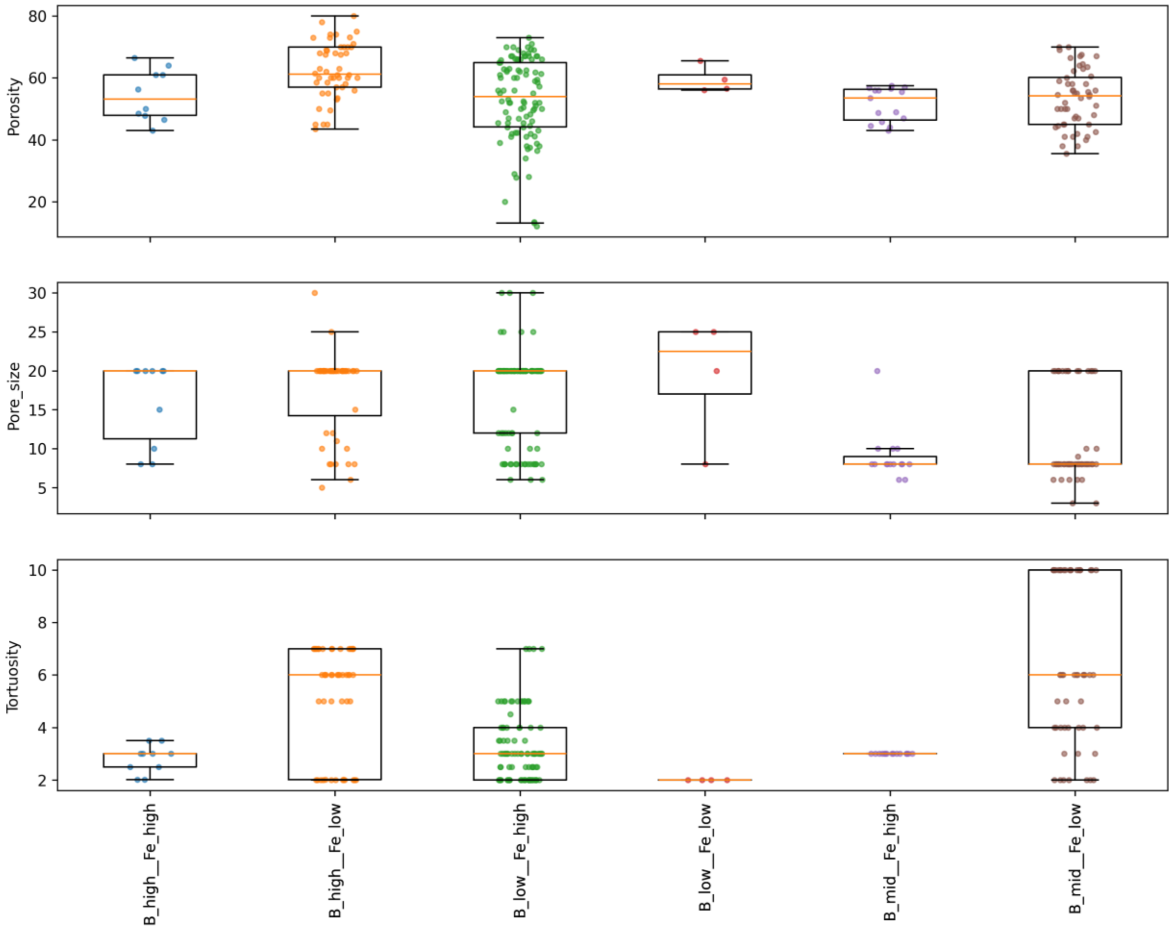

Figure S7 – Box-and-strip plots summarizing the per-experiment distributions of (top) porosity, (middle) characteristic pore size, and (bottom) tortuosity across the same categorical strata defined by basicity class (B high/B mid/B low) and Fe-content class (Fe high/Fe low). Points represent individual experiments; boxes indicate the median and interquartile range with whiskers denoting the data spread within each stratum. These comparisons document that the strata correspond to systematic differences in pellet architecture (the mediators used in the main-text pathway analysis). Tortuosity is reported as discrete "level" assignments in parts of the dataset; therefore, some strata exhibit near-constant values (e.g., $\tau \approx 2$ or $\tau \approx 3$), reflecting discretization rather than an absence of architectural variation.

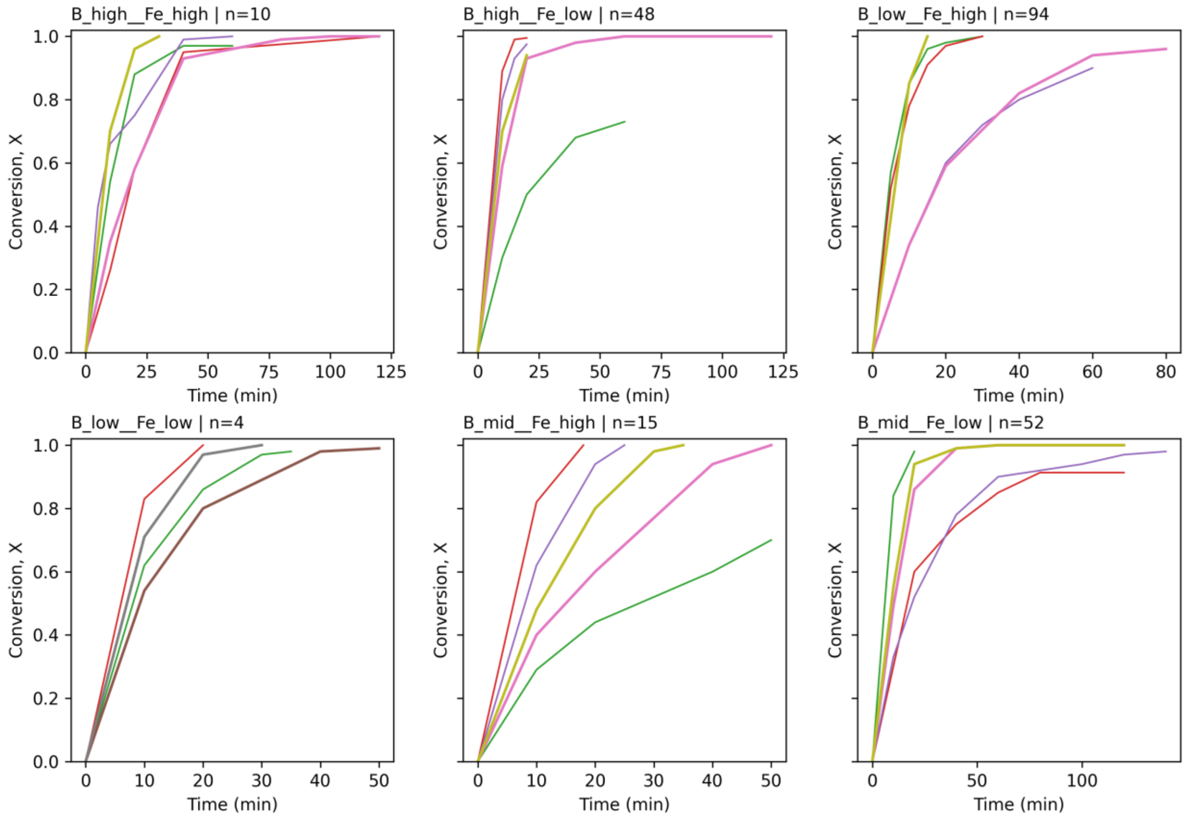

Figure S8 – Representative conversion-time trajectories X(t) stratified by basicity-Fe-content classes. Each panel show examples of a subset of experimentally measured reduction trajectories X(t) for each stratum defined by basicity class (B high, B mid, B low) and Fe-content class (Fe high, Fe low); the number of available experiments in each stratum (n) is indicated in the panel titles. Within each panel, the colored curves correspond to individual experiments selected as a representative subset to illustrate the range of reduction histories within that stratum (no color coding by process variables is implied). Time-axis limits are allowed to vary by stratum to accommodate the observed kinetic spread and to retain visibility of the full conversion progression to high X. This figure supports the staged interpretation adopted in the main analysis by demonstrating that reduction trajectories and the time required to reach a given conversion differ systematically across strata.

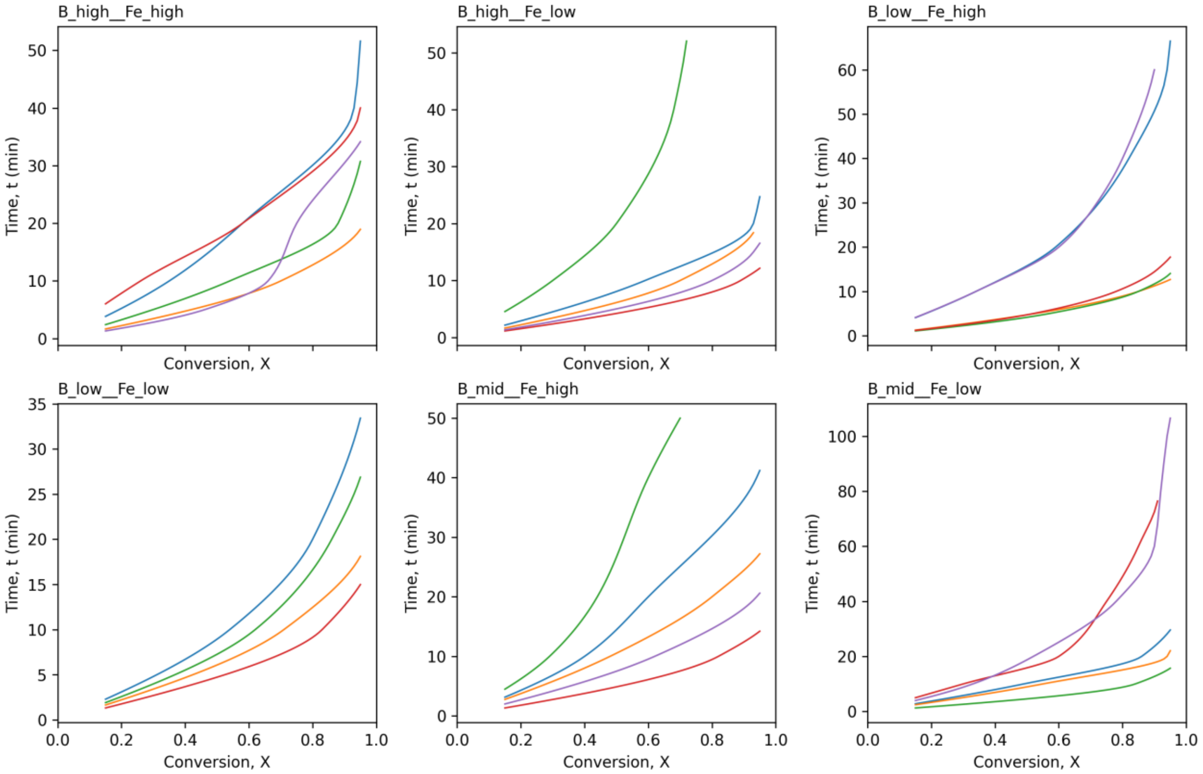

Figure S9 – Representative additive-time trajectories $t(\mathrm{X})$ stratified by basicity-Fe-content classes. Each Panels show conversion-resolved time histories $t(\mathrm{X})$, obtained by monotone inversion of the experimental conversion trajectories $\mathrm{X}(t)$, for each stratum defined by basicity class (B high, B mid, B low) and Fe-content class (Fe high, Fe low). Within each panel, the colored curves correspond to individual experiments selected as a representative subset spanning the within-stratum envelope; the subset intentionally includes condition extremes where present, which appear as steeper late-conversion upturns in $t(\mathrm{X})$. The $t(\mathrm{X})$ representation is the analysis coordinate used for mechanistic deconvolution because it aligns trajectories at a fixed conversion percentage, making late-stage slow-down (product-layer growth and evolving pore access) visually explicit.

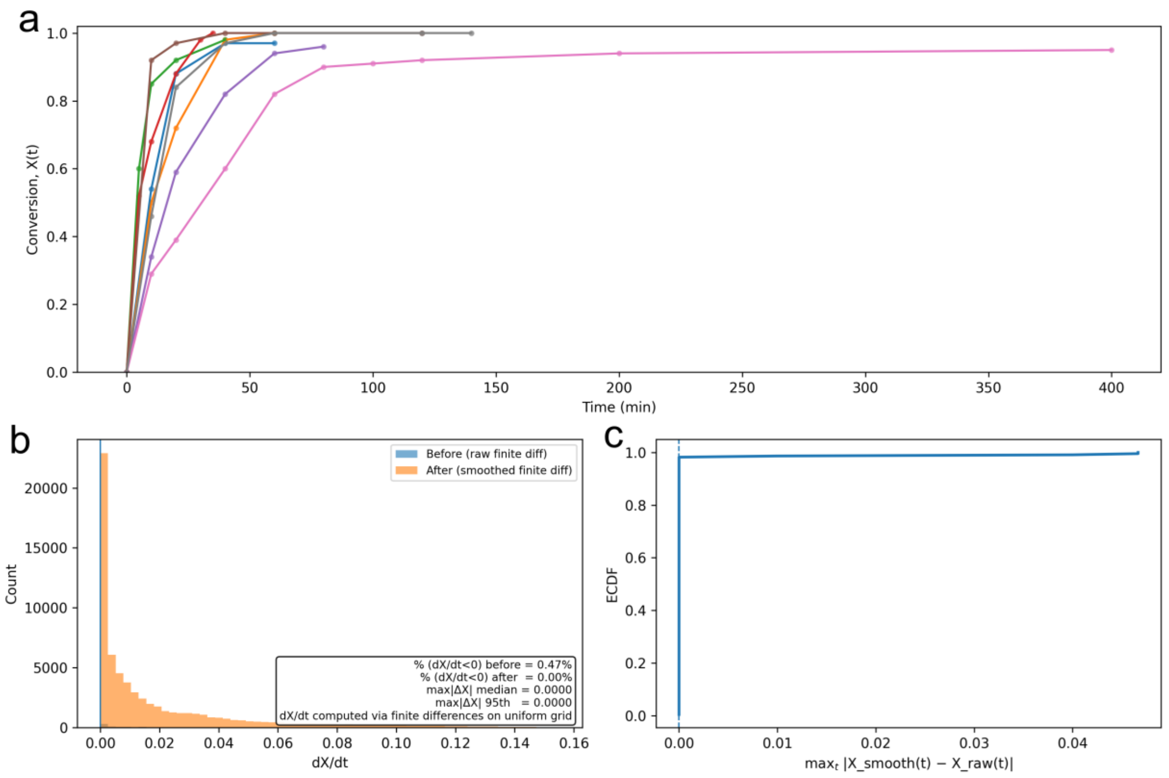

Figure S10 – Trajectory monotonicity enforcement and smoothing diagnostics for conversion-time data. (a) Representative experimental conversion trajectories $X(t)$ after enforcing physically required monotonic increase of conversion with time. (b) Distribution of finite-difference dX/dt values computed on a uniform time grid before processing (raw derivative) and after smoothing with monotonicity enforcement (smoothed derivative); the inset reports the fraction of negative dX/dt values before and after processing, confirming removal of nonphysical back-steps in conversion. (c) Empirical cumulative distribution of the maximum pointwise deviation $\max_t |X_{smooth}(t) - X_{raw}(t)|$, which quantifies the extent of trajectory modification introduced by the smoothing/projection procedure and bounds the perturbation propagated into subsequent $t(X)$ inversion and mechanistic decomposition.

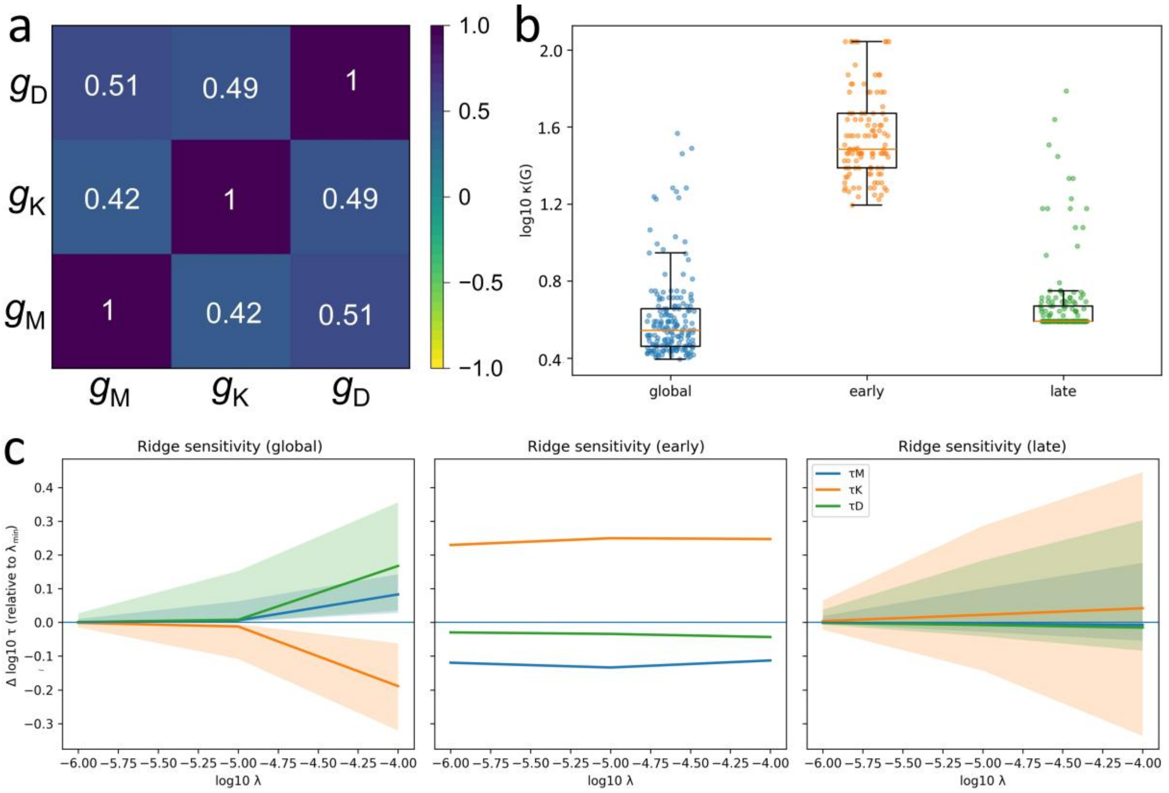

Figure S11 – (a) Pairwise correlation matrix between the SCM basis functions $g_M(X)$, $g_K(X)$, and $g_D(X)$ evaluated over $0 \le X \le 1$, showing that the three contributions are related but not collinear, enabling stable decomposition of $t(X)$ into external/film interaction, interfacial reaction, and internal transport components. (b) Distribution of $\log_{10} \boldsymbol{k}(G)$ for the global, early-conversion, and late-conversion fits, where $\boldsymbol{k}(G)$ is the effective model complexity (effective degrees of freedom) of the ridge-regularized spline representation used to estimate the τ-fields; larger $\boldsymbol{k}(G)$ indicates a more flexible fitted shape after penalization, while smaller $\boldsymbol{k}(G)$ indicates stronger smoothing under the ridge penalty λ. (c) Ridge sensitivity curves showing the change in inferred $\log_{10} \tau$ relative to the minimum-penalty reference, $\Delta\log_{10}\boldsymbol{\tau} = \log_{10}\, \tau(\lambda) - \log_{10}\, \tau(\lambda_{\min})$, as a function of $\log_{10} \lambda$ for the global, early, and late analyses (lines: central estimate; shaded envelopes: variability across resampling/validation repeats used in the analysis).

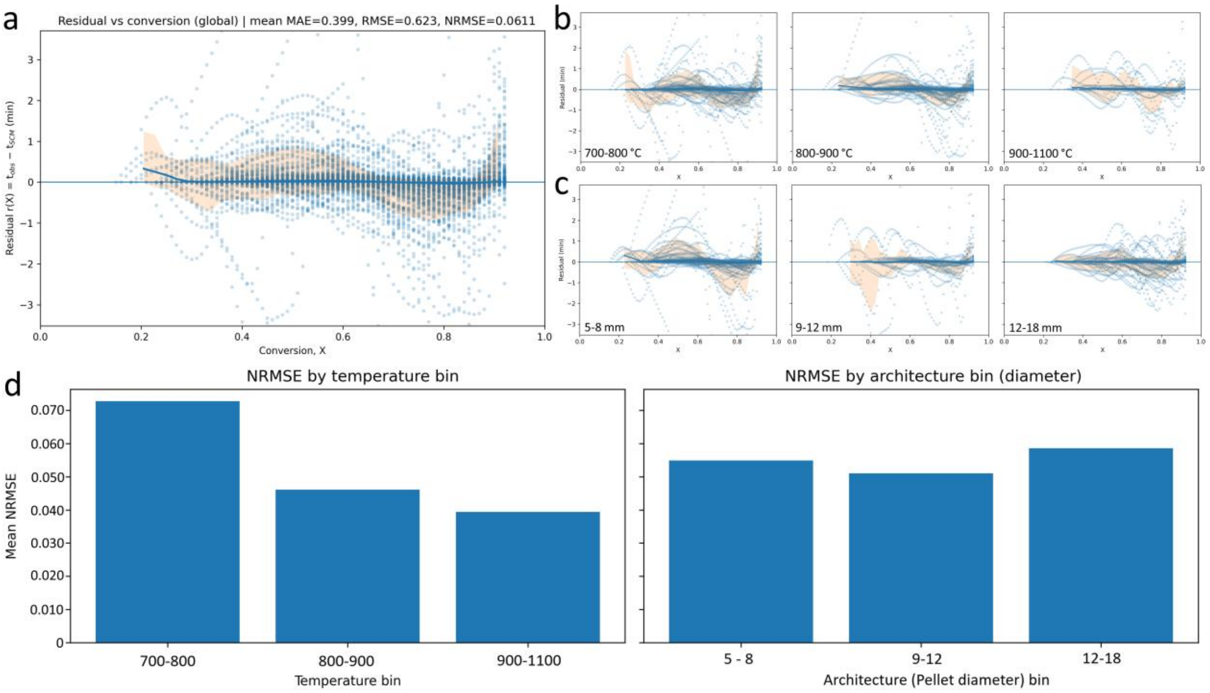

Figure S12 – (a) Global residuals of the conversion-time representation, $r(\mathrm{X}) = t_{\mathrm{obs}}(\mathrm{X}) - t_{\mathrm{SCM}}(\mathrm{X})$, plotted against conversion percentage X for all experiments. The solid line shows the mean residual trend with a smoothed envelope indicating the central spread of residuals as a function of X. (b,c) Residuals plotted as a function of conversion after stratifying the dataset by (b) gas temperature (700-800, 800-900, 900-1100 °C) and (c) pellet diameter (5-8, 9-12, 12-18 mm), highlighting how misfit evolves with operating reducing conditions and pellet size. Residual spread can broaden at high conversion where $t(\mathrm{X})$ steepens, reflecting the increased sensitivity of late-stage kinetics/transport to small changes in effective reduction rate limitations. (d) Mean normalized error by bin for the temperature and diameter strata. Here, MAE and RMSE are computed over all evaluated (X,t) points in each bin, and NRMSE=RMSE/$t_{\text{per-stage range}}$.

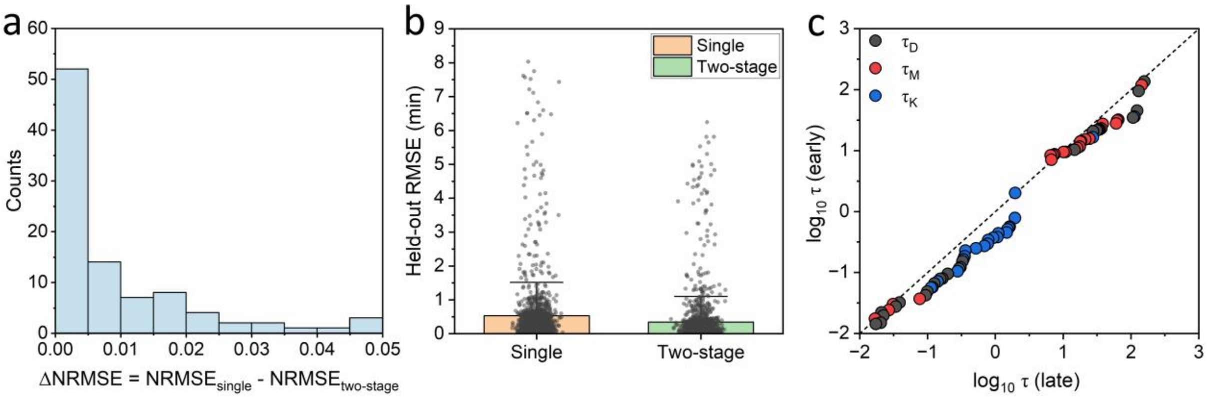

Figure S13 – (a) Distribution of the improvement in normalized error obtained by replacing a single-stage shrinkage-core mixed-control (SCM) fit with a two-stage SCM fit, reported as ΔNRMSE=NRMSE$_{\text{single}}$ − NRMSE$_{\text{two-stage}}$ over all trajectories (positive values indicate improved predictive reconstruction of t(X) under the two-stage representation). (b) Held-out prediction error for the single-stage and two-stage SCM models, reported as RMSE in minutes for each held-out unit (points). Held-out protocol: splits are performed at the trajectory/experiment level (all (t,X) points from a given experiment are kept together), with folds grouped by experiment to prevent within-trajectory leakage; the plotted RMSE corresponds to the out-of-fold reconstruction error of t(X). (c) Relationship between early- and late-stage fitted timescales for the SCM rate-controlling mechanisms, shown as $\log_{10}\tau_{\text{early}}$ *vs.*$\log_{10}\tau_{\text{late}}$ for τ_D (internal transport), τ_M (external/access/film), and τ_K (interfacial reaction). The dashed line indicates equality, highlighting the extent to which the inferred rate-control hierarchy is preserved or reorganized between early and late conversion stages.

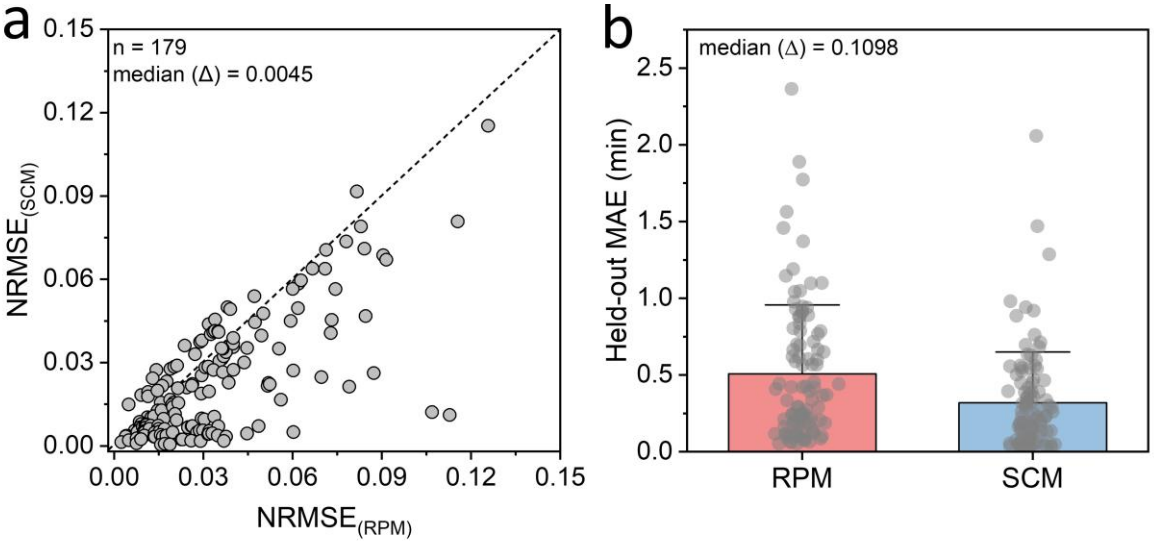

Figure S14 – Baseline model comparison: Random pore model (RPM) versus shrinking-core mixed-control model (SCM) for reconstructing reduction trajectories. (a) Per-trajectory normalized root-mean-square error (NRMSE) obtained from the baseline RPM plotted against the corresponding NRMSE from SCM (each point is one experiment/trajectory; n = 179). The dashed line denotes parity. The annotated median(Δ) reports the median improvement in NRMSE across trajectories, where $\Delta = \text{NRMSE}_{\text{RPM}} - \text{NRMSE}_{\text{SCM}}$ (positive Δ indicates lower error for SCM). (b) Held-out mean absolute error (MAE, minutes) for RPM and SCM under the same cross-validation split strategy (trajectory/experiment-level splits, i.e., all (t,X) points from a given experiment are kept together within a fold to prevent within-trajectory leakage). Points show per-trajectory out-of-fold values; bars summarize the central tendency. The annotated median(Δ) reports the median MAE difference $\Delta = \text{MAE}_{\text{RPM}} - \text{MAE}_{\text{SCM}}$ (positive Δ favors SCM). This benchmark establishes the quantitative advantage of the adopted SCM trajectory representation over RPM in reduction-trajectory reconstruction, prior to the constitutive SCAM analysis of the inferred timescales.

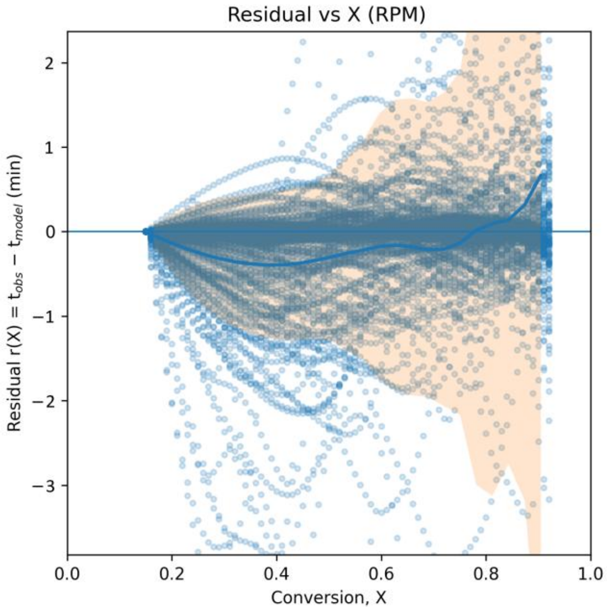

Figure S15 – Residual vs conversion for baseline mechanistic fits (RPM vs SCM). Conversion-resolved residuals, defined as $r(X) = t_{obs}(X) - t_{model}(X)$ (min), are shown for the random pore model (RPM) (left) and shrinking core model (SCM) (right). Each marker corresponds to an individual $t(X)$ data point from the trajectory-inverted kinetics; the horizontal line indicates zero residual (perfect agreement). The solid blue curve summarizes the central tendency of $r(X)$ as a function of conversion (running/binned median), and the shaded envelope shows the corresponding dispersion across the dataset (inter-quantile spread within conversion bins).

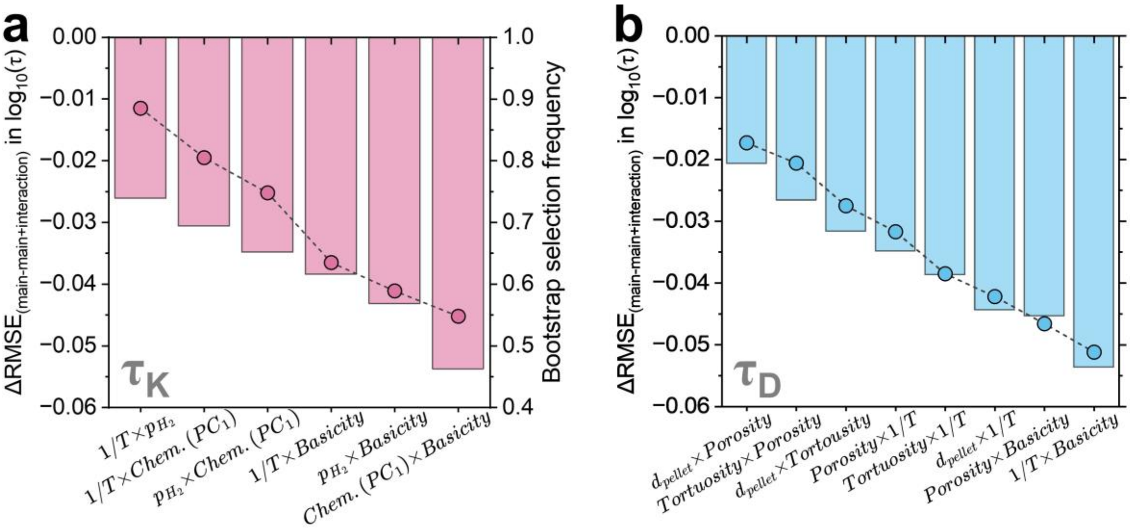

Figure S16 – Interaction-selection stability and predictive value for the timescale models. Interaction terms were screened using a bootstrap resampling procedure and ranked by their contribution to held-out performance. Bars show the change in predictive error upon adding each interaction to the corresponding main-effects model, reported as ΔRMSE in $\log_{10}(\tau)$ units (negative ΔRMSE indicates improved fit). Circles (right axis) report the bootstrap selection frequency, i.e., the fraction of bootstrap replicates in which the interaction is retained among the top-performing candidates. (a) Candidate interactions for τ_K, highlighting coupled thermodynamic driving force and operating-condition effects (e.g., 1/T × pH_2, 1/T × Composition PC_1, and pH_2 × Composition PC_1). (b) Candidate interactions for τ_D, emphasizing architecture–architecture coupling and architecture-composition coupling (e.g., D_{pellet} × Porosity, Porosity × Tortuosity, and Porosity × 1/T).

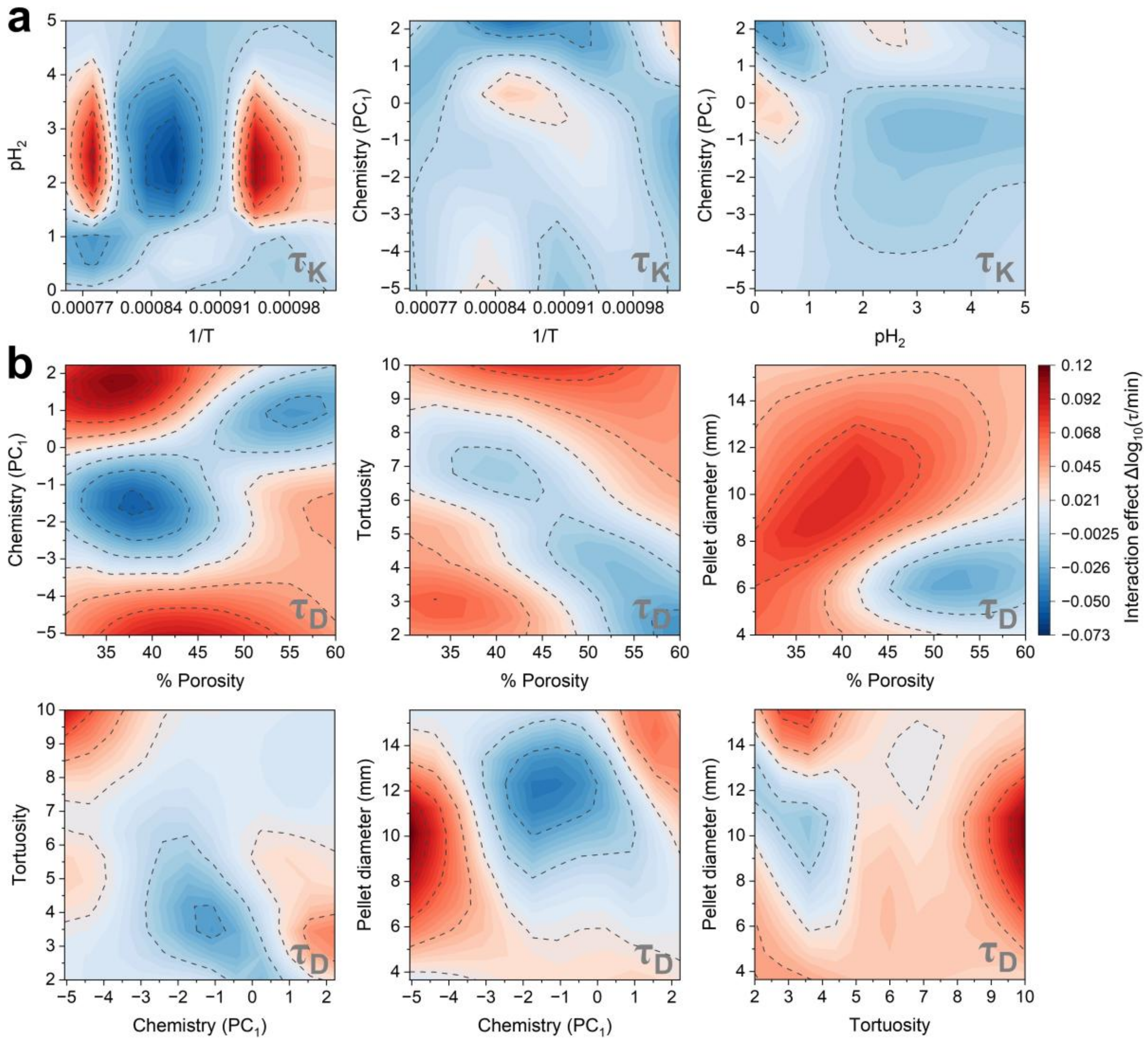

Figure S17 – (a) Fitted pairwise interaction components for the reaction-controlled timescale τ_K: h(1/T, pH_2), h(1/T, Chem(PC_1)), and h(pH_2, Chem(PC_1)). (b) Fitted pairwise interaction components for the diffusion-controlled timescale τ_D: h(ε, Chem(PC_1)), h(ε, τ_{tort}), h(ε, D_{pellet}), h(τ_{tort}, Chem(PC_1)), h(D_{pellet}, Chem(PC_1)), and h(τ_{tort}, D_{pellet}). Colors report the interaction effect in $\Delta\log_{10}(\tau$, min), defined as the fitted bivariate interaction term (deviation from additivity) after centring the interaction surface to zero mean over the training distribution; red indicates a positive interaction (combined conditions increase τ relative to the sum of the corresponding main effects), blue indicates a negative interaction. For each surface, all non-plotted predictors are fixed at their reference values (dataset medians). Axes use 1/T in K^{-1}; pH_2 denotes hydrogen partial pressure in bar; porosity is the per-experiment median (%); tortuosity τ_{tort} is dimensionless; D_{pellet} is in mm; and Chem(PC_1) is the first principal component of pellet composition (increasing with higher Fe and decreasing with higher gangue-oxide content). Dashed contours denote iso-levels of the interaction effect.

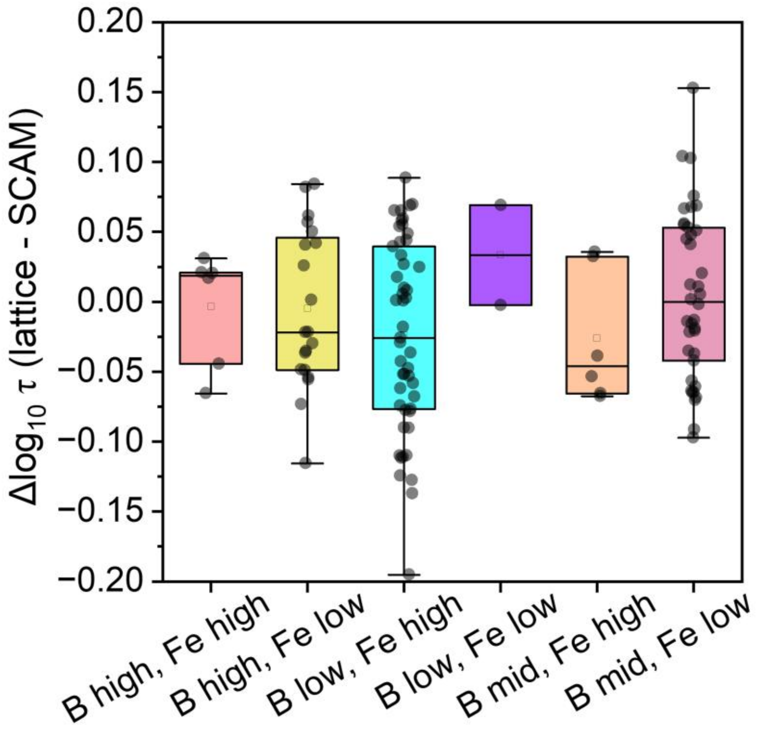

Figure S18 – Box plots of $\Delta\log_{10}\tau = \log_{10}(\tau_{\text{lattice}}) - \log_{10}(\tau_{\text{SCAM}})$ evaluated for each experiment and grouped by the six composition-basicity strata ($B_{\text{high/mid/low}} \times Fe_{\text{high/low}}$). Boxes show the interquartile range with median (central line) and whiskers extending to 1.5×IQR; points are individual experiments. Positive values indicate that the lattice model yields longer characteristic times than the SCAM estimate, and negative values indicate the converse.

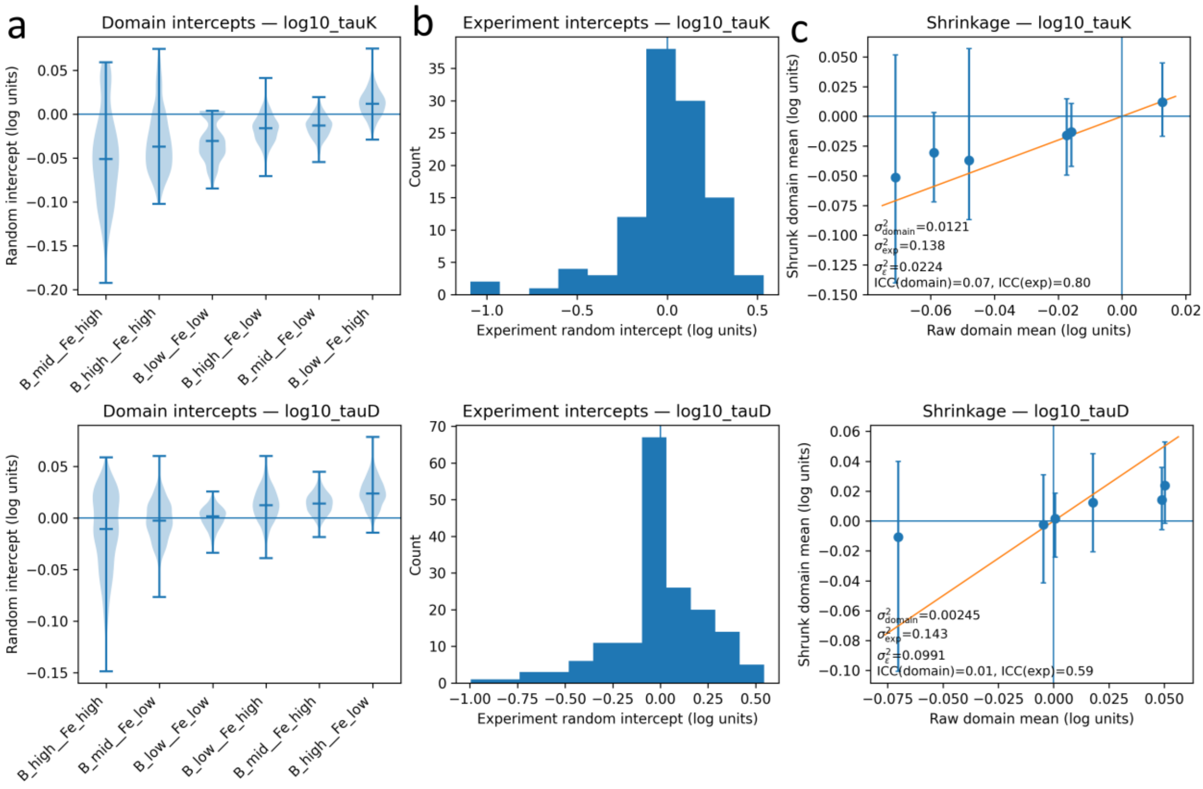

Figure S19 – Hierarchical random intercepts and partial pooling for τ_K and τ_D timescale regimes. Random-intercept variance decomposition for the log-timescale responses $\log_{10} \tau_K$ (top row) and $\log_{10} \tau_D$ (bottom row) under a two-level hierarchical specification with (*i*) domain-level random intercepts (six strata: $B_{high/mid/low} \times Fe_{high/low}$) and (*ii*) experiment-level random intercepts nested within domain. (a) Estimated domain intercept distributions (violin plots) with group means overlaid; values are centered such that zero corresponds to the grand mean. (b) Histogram of experiment-level random intercepts, quantifying within-domain experiment-to-experiment heterogeneity. (c) Shrinkage (partial pooling): raw domain means versus shrunk (partially pooled) domain means; points show posterior/uncertainty intervals for each domain estimate (interval type as implemented in the hierarchical fit), illustrating stronger shrinkage for less precisely estimated domains. Variance components are reported as $\sigma^2_{\mathrm{domain}}$ (between-domain variance), σ^2_{exp} (between-experiment variance within domains), and σ^2_{e} (residual variance). Intraclass correlation coefficients are computed as $ICC_{domain} = \frac{\sigma^2_{domain}}{\sigma^2_{domain}+\sigma^2_{exp}+\sigma^2_e}$, $ICC_{exp} = \frac{\sigma^2_{exp}}{\sigma^2_{domain}+\sigma^2_{exp}+\sigma^2_e}$. The large $\mathrm{ICC}_{\mathrm{exp}}$ for τ_K indicates that most variance in the random-intercept component, whereas τ_D shows a comparatively smaller but non-negligible experiment-level component.

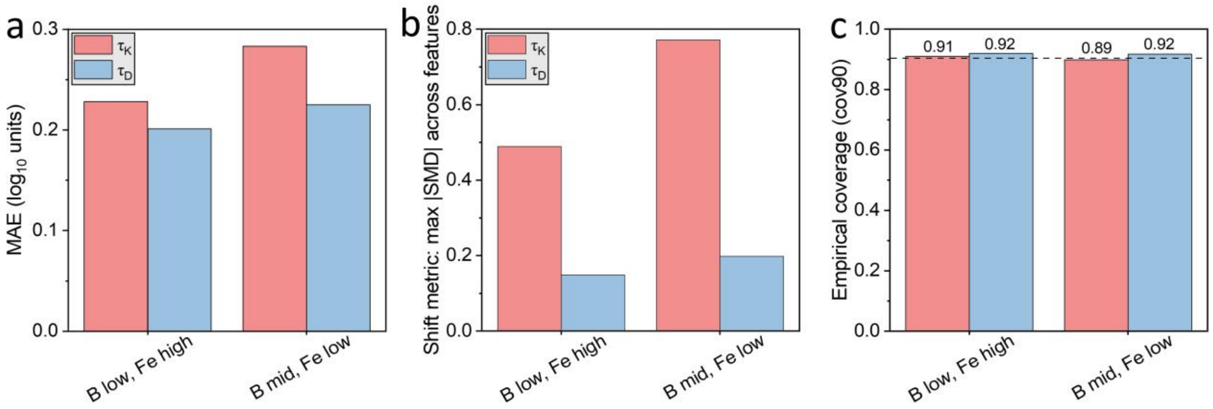

Figure S20 – Performance and shift diagnostics for the domain hold-out evaluation of the stage-specific timescale predictors for $\log_{10}\tau_K$ (red) and $\log_{10}\tau_D$ (blue), shown for two families as representation. (a) Mean absolute error (MAE, in $\log_{10}$ units) on the held-out domain(s) indicated on the x-axis. (b) Covariate-shift summary quantified as $\max_j |SMD_j|$, the maximum absolute standardized mean difference across the input features used by the model (experimental conditions + pellet architecture + composition descriptors). Here $SMD_j = (\mu_j^{train} - \mu_j^{test})/s_j^{pooled}$, with $s_j^{pooled} = \sqrt{(s_{train,j}^2 + s_{test,j}^2)/2}$; continuous features were z-standardized using training-set statistics prior to SMD evaluation, and SMD was computed on the resulting standardized variables. (c) Empirical conformal coverage at nominal 90% (cov_{90}; dashed line), reported separately for τ_K and τ_D on the same held-out domains. Together, these panels relate out-of-domain error to measurable covariate shift and to the validity of uncertainty under domain shift. Despite substantial covariate shift for τ_K (max |SMD| up to ~0.77), predictive errors remain moderate (MAE ≈ 0.23-0.29 log units) and the nominal 90% intervals preserve near-nominal empirical coverage (0.89-0.92), indicating robust uncertainty calibration under composition domain shift within the sampled pellet scale descriptor space. These diagnostics should not be interpreted as calibration for extrapolation beyond the observed support.

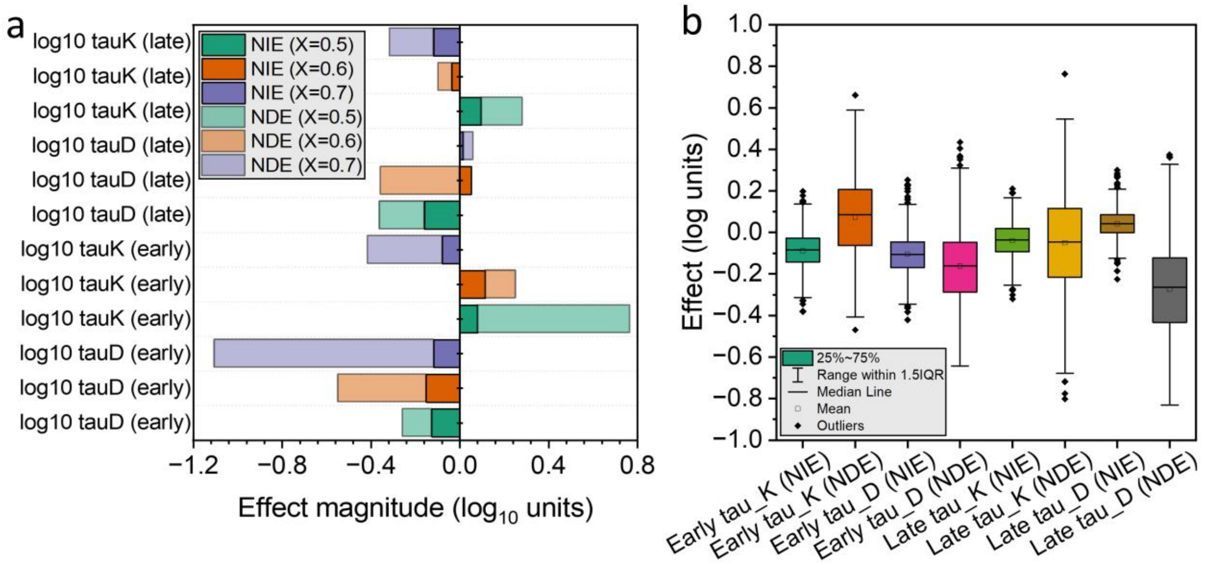

Figure S21 – Estimated mediation effects (NIE/NDE) for early- and late-stage timescales across conversion. Causal-mediation decomposition of the association between the stratified domain label ($B_{high/mid/low}$ × $Fe_{high/low}$) and the fitted stage-specific timescales $\log_{10}\tau_K$ and $\log_{10}\tau_D$, expressed as natural direct effects (NDE) and natural indirect effects (NIE) transmitted through the selected architecture mediators (as defined in the mediation model). All effects are estimated under the specified mediation model and covariate adjustment set and are reported in $\log_{10}$ units relative to the reference domain. (a) Point estimates with uncertainty intervals for NDE and NIE evaluated at representative conversion levels X = 0.5, 0.6, and 0.7 (colors), shown separately for early- and late-stage $\log_{10}\tau_K$ and $\log_{10}\tau_D$. The vertical line at zero denotes no effect; negative values indicate reduced $\log_{10}\tau$ (shorter characteristic time) relative to the reference, and positive values indicate increased $\log_{10}\tau$. (b) Sampling distributions of the corresponding NIE and NDE estimates (box/whisker plots across resamples/replicates as implemented), summarizing median, interquartile range, whiskers (1.5×IQR), mean, and outliers for early- and late-stage $\log_{10}\tau_K$ and $\log_{10}\tau_D$.

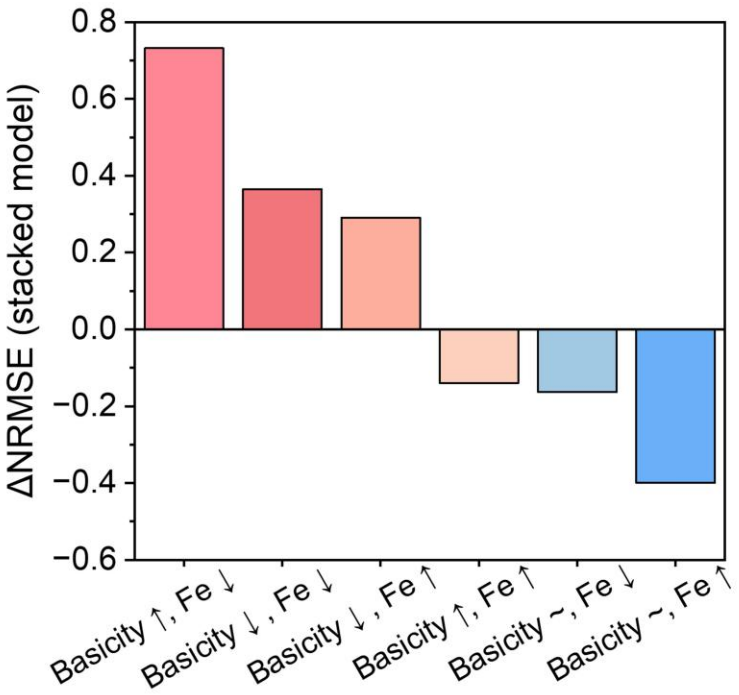

Figure S22 – Stratified error change for the stacked model across basicity-Fe regimes. Bar plot of ΔNRMSE for the stacked predictor evaluated within strata defined by basicity and total Fe content (Fe), with arrows indicating higher (↑), lower (↓), or intermediate (~) levels relative to the dataset stratification thresholds used in the main text. The ordinate reports $\Delta NRMSE = NRMSE_{stratum} - NRMSE_{overall}$ for the stacked model (NRMSE computed on $\log_{10} \tau$ targets using the same normalization as elsewhere). Positive values indicate worse-than-overall predictive performance for that stratum, whereas negative values indicate better-than-overall performance. Performance varies systematically across composition regimes, consistent with regime-dependent microstructure evolution and covariate shift.

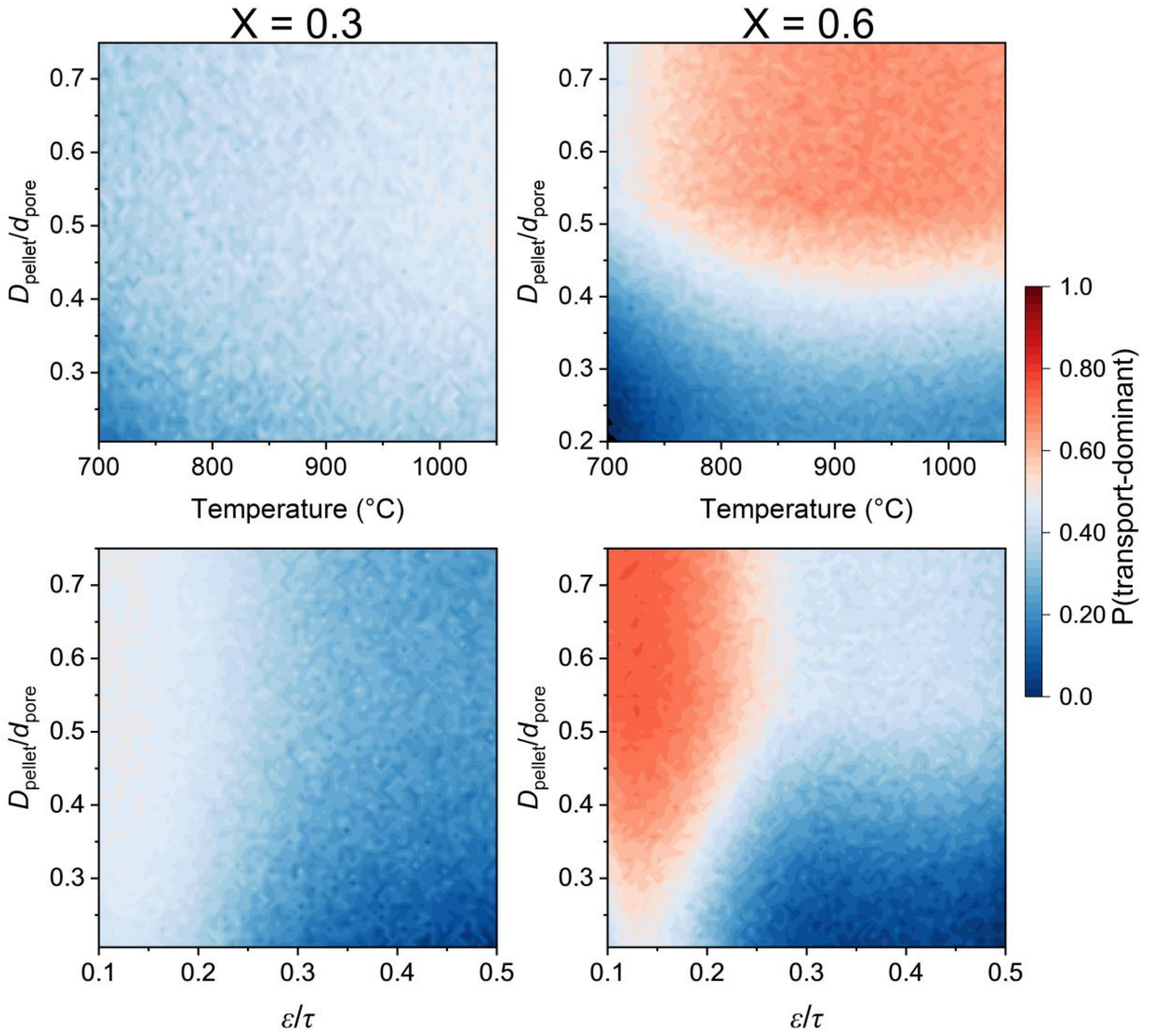

Figure S23 – Conversion-resolved regime probability maps for transport dominance. Two-dimensional probability maps of the transport-dominant regime, P($\tau_D > \tau_K$), evaluated at fixed conversion X using the fitted timescale model. Top row: P($\tau_D > \tau_K$) as a function of gas temperature T_{gas} and the geometry ratio D_{pellet}/d_{pore} at X=0.3 (left) and X=0.6 (right). Bottom row: the same probability as a function of the densification/tortuosity surrogate ε/τ and D_{pellet}/d_{pore} X=0.3 (left) and X=0.6 (right). Color indicates P($\tau_D > \tau_K$) (blue: reaction-dominant; red: transport-dominant).

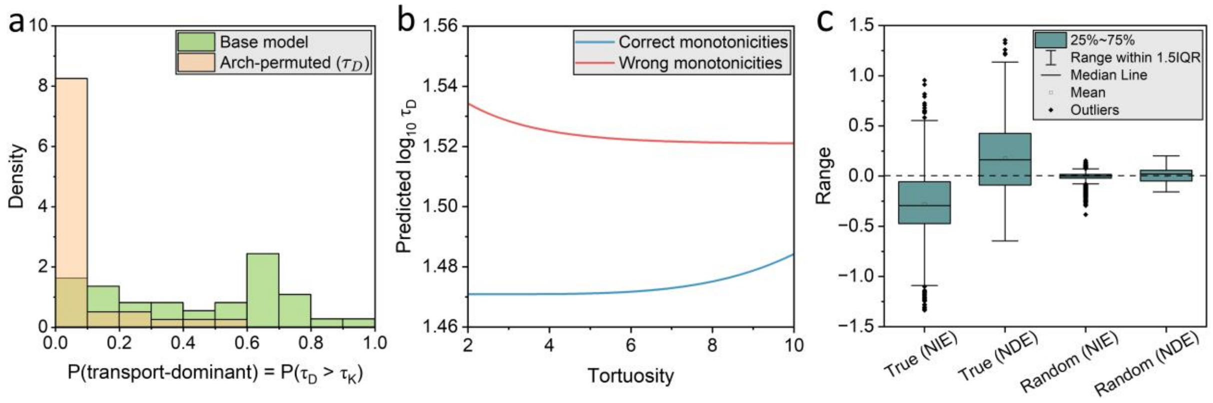

Figure S24 – (a) Permutation falsification at the experiment level: the distribution of inferred regime probability P($\tau_D > \tau_K$) for the base model (green) versus an architecture-permuted control in which the τ_D-relevant architecture descriptors are shuffled across experiments while preserving within-trajectory structure (orange). The permutation collapses the structured regime inference toward near-zero probabilities, indicating that the regime map relies on physically aligned architecture information rather than incidental fitting flexibility. (b) Wrong-physics constraint test: predicted $\log_{10} \tau_D$ *vs.* tortuosity under the intended monotonicity constraints (blue) compared with a refit imposing intentionally incorrect monotonicity directions (red), producing a qualitatively inconsistent partial-dependence and demonstrating that constraint direction is consequential. (c) Negative-control mediation check: distributions of effect range for the true mediation specification (NIE/NDE) *vs.* randomized controls (mediator), where the randomized effects concentrate near zero while the true specification retains structured, non-zero ranges.

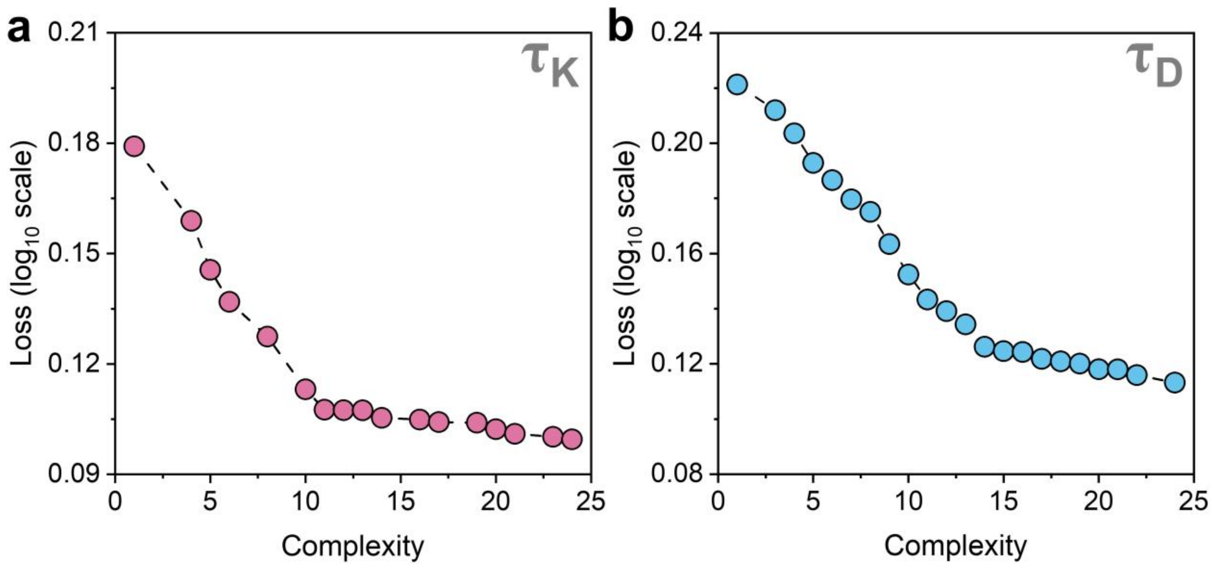

Figure S25 – Multi-objective trade-off between predictive loss and model complexity used to select the final symbolic-regression expressions for (a) τ_K and (b) τ_D. The "chosen balance" marks the selected Pareto-compromise solution at the elbow/knee: additional complexity yields only marginal loss reduction, while simpler models incur a noticeable loss penalty.

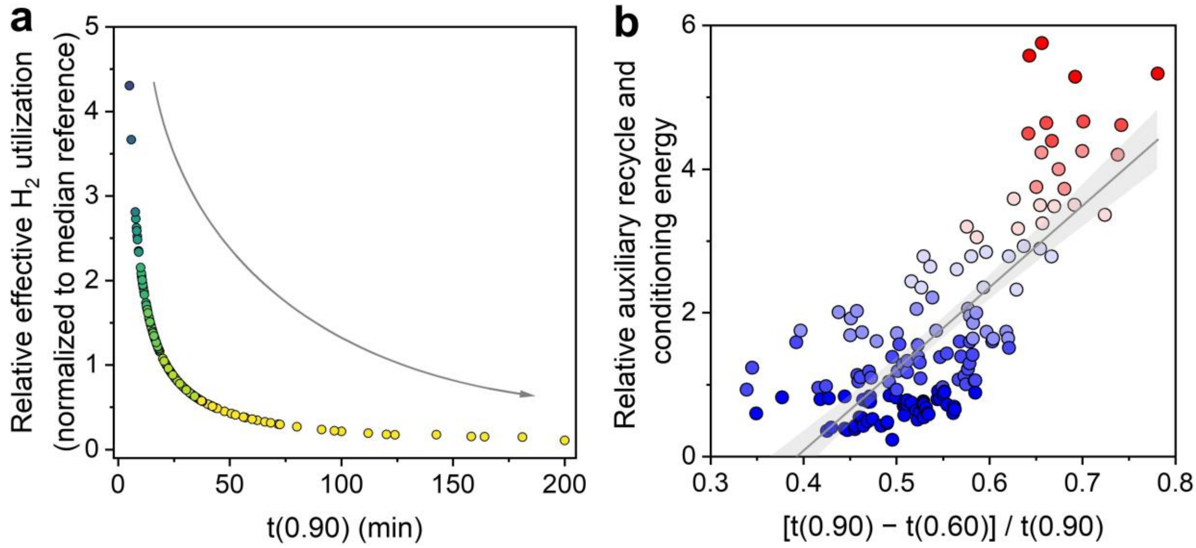

Figure S26 – Illustrative translation of pellet scale kinetic observables into process-relevant hydrogen efficiency metrics under fixed operating assumptions. (a) Relative effective H_2 utilization, normalized to the median reference case, plotted against the pellet scale time required to reach X=0.90. Under the fixed hydrogen feed assumption used here, shorter t(0.90) corresponds to higher effective utilization, whereas prolonged reduction to the same target conversion lowers effective utilization. The gray guide curve indicates the expected inverse scaling under this scenario. (b) Relative auxiliary recycle and conditioning energy plotted against the late stage time fraction, [t(0.90)−t(0.60)]/t(0.90). A larger late-stage fraction is associated with a larger auxiliary energy penalty under the fixed circulation assumption used for this illustrative translation. The solid line indicates the central trend and the shaded band is shown only to guide the eye. In both panels, the color scale reflects the magnitude of the plotted y variable. These quantities are presented as scenario-based pellet-to-process translation metrics derived from the compiled pellet-scale dataset.

Table S1 – Validated pellet scale descriptor envelope used for trajectory resolved timescale inference and constitutive mapping. ***Note:*** All constitutive maps, symbolic expressions, and regime assignments reported in the main text are restricted to this experimentally sampled envelope. Quantitative extrapolation beyond these ranges was not validated in the present study.

**Quantity**	**Value**
Number of experiments	227
Total time-conversion points	17690
Gas temperature, T_{gas}	700 - 1100 °C
Total pressure, P_{gas}	1 - 6.8 bar
Pure-H_2 subset	194/227 experiments
Pellet diameter, D_{pellet}	5 - 18 mm
Green density	2.7 - 4.4 g/cm^3
Composition domains	6 (High Basicity-High Fe content, High Basicity-Low Fe content, Low Basicity-High Fe content, Low Basicity-Low Fe content, Intermediate Basicity-High Fe content, Intermediate Basicity-Low Fe content)
Total Fe (wt.%)	62.50 - 68.50 (median 65.50)
CaO (wt.%)	0.50 - 8.50 (median 3.00)
MgO (wt.%)	0.50 - 4.50 (median 1.50)
Al_2O_3 (wt.%)	0.00 - 2.80 (median 1.35)
SiO_2 (wt.%)	0.10 - 7.70 (median 3.20)
Basicity index (–)	0.01 - 17.00 (median 0.86)
Composition PCA scores	Composition PC_1: -1.84 – 2.50<br>Composition PC_2: -2.12 – 1.70

Table S2 – Composition PCA definition (chem PC_1, chem PC_2) and loadings used throughout the analysis. PCA was performed on the standardized composition vector [Fe, CaO, MgO, Al_2O_3, SiO_2, TiO_2], using z-scoring based on the dataset mean (μ) and standard deviation (σ) reported here (i.e., each variable centered by μ and scaled by σ prior to PCA). The table lists the resulting PC_1 and PC_2 loadings with a fixed sign convention. PC loadings are the eigenvector coefficients on standardized variables (unit-length PCs) and signs were chosen such that PC_1 has positive Fe loading and PC_2 has positive TiO_2 loading.

Variable	Mean (μ)	Standard deviation (σ)	PC_1 loading	PC_2 loading
Fe	66.106	3.726	0.473	0.101
CaO	0.992	0.933	-0.341	-0.521
MgO	0.709	1.320	-0.430	0.246
Al_2O_3	0.738	0.828	-0.472	0.063
SiO_2	3.130	2.318	-0.448	-0.190
TiO_2	0.0455	0.097	-0.223	0.785

Physical interpretation: composition coordinate chem PC_1 increases with Fe and decreases with oxide additions (CaO/MgO/Al_2O_3/SiO_2), consistent with its positive Fe loading and negative oxide loadings; chem PC_2 is dominated by TiO_2 (largest positive loading) with additional contributions from CaO/SiO_2.

Table S3 – Explained variance and metallurgical interpretation of composition principal components (chem PC1, chem PC2). Explained variance ratios (and corresponding percentages) for PC_1 and PC_2 from the standardized composition PCA defined in Table S2, along with a concise, loading-consistent physical interpretation of each component (sign convention fixed to match Table S2). Explained variance ratios correspond to eigenvalues of the covariance matrix of standardized variables. PC_1+PC_2 explain 98.9% of total variance; remaining PCs together explain 1.1%.

**Component**	**Explained variance ratio**	**Explained variance (%)**	**One-line physical interpretation**
PC_1 (Composition_PC_1)	0.8074	80.7	Higher Fe content (Fe↑) versus higher non-Fe oxide content (gangue/basicity modifiers) (CaO, MgO, Al_2O_3, SiO_2, TiO_2 ↓).
PC_2 (Composition_PC_2)	0.1814	18.1	Dominated by TiO_2 (↑) and MgO (↑) contrasted against CaO (↓) and SiO2 (↓).

Table S4 – Full feature definitions used in this study.

**Feature**	**Physical definition**	**Units**
Reduction time	Elapsed reduction time corresponding to each conversion measurement in the TGA trajectory	min
Pellet diameter	Pellet diameter	Mm
Density	Pellet bulk density	$Kg{\cdot}m^{-3}$
Fe	Bulk composition: Fe content in the pellet	wt.%
CaO	Bulk composition: CaO content in the pellet	wt.%
MgO	Bulk composition: MgO content in the pellet	wt.%
Al_2O_3	Bulk composition: Al_2O_3 content in the pellet	wt.%
SiO_2	Bulk composition: SiO_2 content in the pellet	wt.%
Basicity index	Basicity index (dimensionless) used to characterize gangue basic/acidic balance	dimensionless
TiO_2	Bulk composition: TiO_2 content in the pellet	wt.%
Other elements	Minor elements/oxides not explicitly enumerated	wt.%
T_{gas}	Reducing gas temperature	°C
P_{gas}	Total system pressure of the reducing gas	bar
H_2 amount	Hydrogen content in the inlet gas mixture	%
G_a acidic	Acidic gangue index	wt.% (oxide-sum)
G_b basic	Basic gangue index	wt.% (oxide-sum)
H_2	Hydrogen content in the inlet gas mixture	%
CO	Carbon monoxide content in the inlet gas mixture (complementary to H_2 when H_2+CO=100%)	%
Conversion	Reduction degree / fractional oxygen removal expressed as percent conversion	%
Porosity	Pellet porosity	%
Pore size	Characteristic pore-size	µm
Tortuosity	Tortuosity level	dimensionless (discretized)
Composition PC_1	Composition PCA score (PC_1) computed from z-scored oxide composition variables.	dimensionless (score)
Composition PC_2	Composition PCA score (PC_2) computed from z-scored oxide composition variables.	dimensionless (score)
Chem domain	Rule-based composition-domain label derived from composition descriptors (Basicity index and Fe content thresholds).	categorical label

Table S5 – Per-experiment SCM timescales and fit metrics. For each experiment (unique Experiment ID), we report the best-fit SCM timescales τ_M, τ_K, and τ_D obtained from the selected SCM variant (SCM variant), together with the RMSE of the reconstructed $t(X)$ trajectory (RMSE) and the number of $t(X)$ data points used (n).

(**Note:** τ values are estimated under nonnegativity. τ=0 indicates the corresponding SCM contribution was inactive (selected out / at the lower bound) in the best-fit representation for that experiment, not a literal zero physical rate control. In practice, τ=0 means the optimizer places that contribution at the boundary within numerical tolerance (effectively negligible compared with the other terms over the observed conversion window)).

Experiment ID	Composition domain	SCM variant	τ_M (min)	τ_K (min)	τ_D (min)	RMSE	n
019d932d677f	B low, Fe high	single	0.0	25.1	196.1	1.035	41
02e34e18a20d	B low, Fe high	single	0.0	103.4	15.7	1.977	62
06b59258e4f5	B intermediate, Fe low	two stage	0.0	24.1	185.3	1.646	60
07b61a08c085	B intermediate, Fe low	two stage	0.0	17.5	175.7	2.041	66
0826a7b58f37	B intermediate, Fe low	single	0.0	9.2	18.2	0.083	34
0a2c2f3a0bf1	B intermediate, Fe low	single	0.0	6.5	30.5	0.345	47
0f918211bb06	B intermediate, Fe low	single	0.0	16.8	11.7	0.389	42
0ff3b2e61d86	B intermediate, Fe low	single	0.0	9.0	58.0	0.300	65
104bf2f58100	B intermediate, Fe low	single	0.0	30.6	66.6	0.336	56
121f83b32771	B low, Fe high	two stage	0.0	10.1	25.7	0.029	23
1320a1460e5b	B low, Fe high	single	0.0	2.6	13.6	0.062	30
15feea7852ac	B intermediate, Fe high	single	0.0	7.7	19.7	0.006	28
174ec3cd480e	B low, Fe high	single	0.0	38.7	173.7	1.803	60
175b7f0349a9	B low, Fe high	two stage	4.9	7.0	0.0	0.028	37
17c5d09567dd	B low, Fe high	two stage	0.0	54.4	18.9	0.025	21
188f1dee2363	B low, Fe high	two stage	0.0	0.0	14.6	0.221	42
18c8155d0b2a	B intermediate, Fe low	two stage	3.1	30.5	0.0	0.153	28
1c75c91cab91	B intermediate, Fe low	two stage	0.0	11.5	92.4	1.516	61
1dcad8093ced	B intermediate, Fe low	single	0.0	15.7	95.0	0.474	58
1dfb510c71fe	B low, Fe low	single	0.0	0.0	42.1	0.169	38
1e3bef5a3991	B intermediate, Fe low	single	0.0	0.0	35.4	0.869	47
1e91179fb647	B intermediate, Fe low	single	0.0	0.0	26.5	0.617	42
1e9d5e4e906c	B low, Fe high	single	0.0	13.7	6.6	0.051	36
1ffd8bda8262	B low, Fe high	two stage	0.0	0.0	81.0	1.576	45
226f4ca55eeb	B low, Fe high	single	0.9	9.6	40.9	0.156	42
24249c179e8c	B intermediate, Fe low	two stage	2.5	7.8	28.9	0.160	45
253451d1fa6d	B intermediate, Fe low	single	12.6	12.4	0.0	0.044	33
2583ddd7696d	B low, Fe high	single	0.0	27.1	89.7	1.156	53
25b936dde013	B intermediate, Fe low	single	0.0	6.1	20.1	0.658	54
26b8b4d2cc05	B intermediate, Fe low	single	0.0	14.6	35.8	0.170	54
298e7c0c2625	B low, Fe high	two stage	0.0	0.0	53.0	0.352	39
2a17d020b44d	B intermediate, Fe low	two stage	10.8	11.8	0.0	0.044	37
2aa1432d76af	B low, Fe high	two stage	0.0	7.0	55.5	1.273	54
2c2de852080d	B low, Fe high	single	0.0	35.0	190.1	1.682	38

2da4c438bd0c	B intermediate, Fe low	single	38.5	0.0	143.3	1.226	72
2fa4cba801b5	B intermediate, Fe low	single	0.0	0.0	42.2	1.847	53
2fb613ffcd43	B intermediate, Fe low	single	0.0	0.0	31.6	0.689	46
31711438c138	B low, Fe high	two stage	0.0	0.0	184.2	2.045	62
334fffb2ec92	B low, Fe high	two stage	0.0	17.5	127.5	0.664	37
349cb084fea4	B low, Fe high	two stage	7.7	33.5	4.3	0.035	33
355b228e2db1	B intermediate, Fe low	single	0.0	0.0	43.3	1.872	45
35ef03f9fa00	B low, Fe high	single	0.0	0.0	43.9	0.338	42
3704e3a9743f	B low, Fe high	single	0.0	0.0	49.6	0.513	57
370d7ccd651d	B low, Fe high	two stage	0.0	14.0	42.0	0.721	62
374514a4e552	B low, Fe high	single	0.0	0.0	43.1	0.965	35
38cfb9a82c8d	B low, Fe high	single	0.0	37.9	7.8	0.287	40
3ab8a8b4e704	B intermediate, Fe low	single	0.0	21.2	86.4	0.055	58
3c770d1a8ecb	B low, Fe low	single	0.3	48.3	0.0	0.047	30
3de18f56e391	B low, Fe high	single	0.0	93.1	51.8	1.502	56
3e66090ef275	B low, Fe high	single	0.0	0.0	15.7	0.140	27
409063012afb	B intermediate, Fe low	two stage	0.0	0.0	147.8	1.583	42
41bec6a60048	B low, Fe high	single	5.8	0.0	74.5	0.267	58
42f9151a3977	B low, Fe high	single	0.0	0.0	57.0	1.215	38
45e43c5df275	B low, Fe high	single	0.0	72.7	6.6	0.169	64
4658505b37a3	B low, Fe high	two stage	22.7	0.0	192.2	0.559	42
47d41a71ccc8	B intermediate, Fe low	two stage	0.0	0.0	73.9	1.814	35
4827ac87c5c3	B low, Fe high	two stage	0.0	0.0	137.6	0.980	46
48a09b6106af	B intermediate, Fe low	two stage	0.0	87.0	3.9	0.150	50
499350f45dc5	B intermediate, Fe high	single	0.0	10.5	32.4	0.027	34
49ac498b7138	B low, Fe high	single	5.3	23.3	0.0	0.066	33
49fefcf15a4a	B low, Fe high	single	0.0	3.6	17.1	0.050	35
4a80a0f814b1	B low, Fe high	single	3.7	11.4	0.0	0.020	26
4bdc0ddd18b9	B low, Fe high	two stage	0.0	0.0	20.4	0.537	35
4bfe14bdded2	B low, Fe high	two stage	0.0	68.2	2.6	1.096	43
4e1af515d3c6	B intermediate, Fe low	two stage	0.5	6.8	21.4	0.030	26
4f7680dbc609	B low, Fe high	single	0.0	14.8	3.7	0.060	28
4f8b8cd3aa03	B intermediate, Fe low	single	0.0	21.6	139.9	1.476	58
5118946bbdad	B intermediate, Fe low	single	0.0	13.7	33.6	0.126	54
51de9fe3ce17	B high, Fe high	single	0.0	0.0	19.5	0.268	22
5700ab8bd378	B low, Fe high	single	0.0	0.0	223.9	2.024	35
5957158b0d93	B low, Fe high	single	0.0	3.1	12.3	0.064	33
5bdfc921d1ac	B intermediate, Fe high	two stage	0.0	0.0	142.5	1.664	40
6241b202083b	B low, Fe high	two stage	0.0	0.0	91.9	1.147	56
6284bdd3aa3a	B intermediate, Fe low	single	0.0	0.0	62.4	0.586	58
64582dc87eb8	B intermediate, Fe low	single	0.0	7.5	19.5	0.030	26
646a8e78fd8b	B low, Fe high	single	5.9	8.6	0.0	0.008	24
6b213afac5a9	B low, Fe high	two stage	0.0	0.0	57.4	2.335	50
6b7f66781527	B low, Fe high	two stage	0.0	0.0	43.9	0.959	48
6b97b571e5fa	B intermediate, Fe low	single	0.0	25.7	95.0	0.474	58
6c1bbb96c309	B intermediate, Fe low	single	0.0	7.0	101.2	0.124	52

6cd6996d667b	B intermediate, Fe low	single	18.8	11.3	0.0	0.031	23
6dc02e29dbd6	B low, Fe high	single	9.6	19.6	0.0	0.004	23
6ec333f05998	B intermediate, Fe low	single	0.0	3.1	158.1	2.027	69
6f8d2b7389ef	B high, Fe high	single	0.0	15.9	37.9	0.957	62
70ea232c87be	B low, Fe high	single	16.4	0.0	241.4	1.769	28
70fbc2392deb	B intermediate, Fe low	single	0.0	0.0	56.5	1.077	40
732db0a550eb	B intermediate, Fe low	two stage	7.2	14.1	0.0	0.082	46
73880b600cef	B low, Fe high	two stage	0.0	7.1	8.8	0.090	29
74638ac99306	B intermediate, Fe low	two stage	6.1	65.7	0.0	0.252	67
77c83d756709	B low, Fe high	two stage	0.0	0.0	19.8	0.239	30
7a51f86cfc28	B intermediate, Fe low	single	0.0	72.1	152.8	1.794	69
7d414ff9d89c	B intermediate, Fe low	single	0.0	0.0	32.6	0.718	30
7da301ac5980	B intermediate, Fe high	single	0.0	0.0	243.5	0.907	37
7fd13c359aaf	B low, Fe high	single	2.9	28.1	95.7	0.536	59
80bc82c6cdd7	B intermediate, Fe low	single	0.0	0.0	32.5	0.905	38
81114b783f1d	B low, Fe high	single	0.0	10.4	40.3	0.938	59
8111f79f2c11	B high, Fe high	two stage	19.8	0.0	41.0	1.879	50
82eed7ae12ff	B intermediate, Fe low	single	0.0	0.1	29.0	0.456	44
8465d957087c	B intermediate, Fe low	single	0.0	0.0	39.6	0.515	50
8bfc36595cc2	B low, Fe high	single	5.6	35.2	0.0	0.100	40
917160e6e9f4	B low, Fe high	single	0.0	63.9	30.0	2.203	64
91b3938dac73	B low, Fe high	single	9.9	0.0	15.1	0.072	45
91dbaa39dbdf	B intermediate, Fe low	single	0.0	0.0	36.4	1.166	58
930d4f8be12a	B intermediate, Fe high	two stage	5.2	0.0	40.3	0.250	28
93723ae429d1	B intermediate, Fe high	two stage	16.7	0.0	131.2	1.125	39
94f854572ab1	B low, Fe high	two stage	0.0	12.0	70.6	0.351	54
954b77122102	B intermediate, Fe high	two stage	37.8	0.0	0.0	0.862	49
95b6ef3d55c3	B high, Fe high	single	3.0	16.4	34.8	0.199	58
961406ef4dab	B low, Fe high	single	0.0	1.2	15.2	0.105	33
98129a814821	B intermediate, Fe low	single	0.0	18.2	105.9	1.763	46
9984998a0bfa	B low, Fe high	single	22.1	4.6	0.0	0.094	51
9a304252b177	B intermediate, Fe high	single	7.6	26.0	7.5	0.040	36
9b4419be220e	B low, Fe low	single	1.5	15.1	30.6	0.012	22
9e4a24bc94a0	B low, Fe high	single	3.9	11.3	0.0	0.078	33
9ef4699ee2d1	B low, Fe high	two stage	0.0	0.0	35.0	2.258	57
9fd5a7acdf16	B low, Fe high	single	0.0	0.0	26.5	0.228	36
a289c2e48332	B intermediate, Fe low	two stage	0.0	16.7	81.5	1.893	51
a3555f518465	B intermediate, Fe low	single	6.4	27.9	0.0	0.161	27
a502b077e9dc	B intermediate, Fe low	two stage	0.0	11.3	14.8	0.059	33
a820c8f0ea30	B intermediate, Fe low	two stage	0.0	12.5	77.2	1.696	62
a8585eb46d33	B low, Fe high	two stage	1.3	2.2	48.6	0.103	54
ab610141272d	B intermediate, Fe high	single	0.0	7.7	22.5	0.004	30
aeabe1bcf594	B low, Fe high	single	0.0	17.8	189.3	1.651	65
aec874b235cd	B intermediate, Fe high	single	2.8	9.2	19.5	0.057	45
af250bf29797	B intermediate, Fe low	single	7.1	60.2	0.0	2.018	64
b365e8e658c2	B intermediate, Fe low	single	0.0	7.5	21.6	0.553	33

b5c4fd9e4a4d	B high, Fe high	single	0.0	0.0	27.7	0.569	38
b7d9fe249bb2	B intermediate, Fe low	single	0.0	7.5	21.7	0.020	21
bbacb3ea2ae9	B intermediate, Fe low	single	0.0	0.0	54.1	1.464	33
bd2515644c10	B low, Fe high	single	0.0	0.0	554.4	1.189	34
bf484beb216c	B high, Fe high	single	0.0	0.0	26.6	0.111	27
bf6aa3c0f45f	B low, Fe high	two stage	0.0	2.8	51.2	0.907	58
c00a75a1ff7c	B low, Fe high	single	4.7	12.9	0.0	0.016	23
c418aac4d2df	B low, Fe high	two stage	26.4	0.0	0.0	0.721	53
c4be7cd430c2	B low, Fe high	single	0.0	0.0	23.4	1.374	33
c73e235d6971	B low, Fe high	two stage	32.0	0.0	60.6	1.278	64
cb57732d6357	B intermediate, Fe low	two stage	0.0	16.3	62.3	1.587	56
cb6b4cab16ba	B low, Fe high	two stage	10.8	0.0	13.8	0.081	47
cba65a9c23c6	B intermediate, Fe low	single	0.0	0.0	48.4	1.119	51
cc0d00bc9c0f	B low, Fe high	single	0.0	0.0	502.4	1.683	30
cdc4057fa267	B low, Fe high	single	7.2	7.3	0.0	0.018	26
d04841755858	B low, Fe high	two stage	0.0	10.5	13.9	0.055	40
d0bca864ea3f	B low, Fe high	two stage	0.0	4.7	17.5	0.078	40
d139341e9a95	B low, Fe high	two stage	0.0	2.1	22.0	0.211	36
d154cfe8a93f	B intermediate, Fe low	two stage	13.9	0.0	32.4	0.125	60
d1daa7279a74	B low, Fe high	two stage	12.1	0.0	24.2	0.084	42
d2c75d016fc3	B intermediate, Fe low	two stage	0.0	0.0	28.4	1.044	50
d439496a250a	B low, Fe high	two stage	7.1	14.0	21.5	0.037	40
d468908ff2e8	B low, Fe high	single	0.0	0.0	19.6	0.223	34
d724788055d5	B intermediate, Fe low	single	0.0	36.2	70.7	1.294	59
d8ab1324ea7d	B low, Fe high	single	0.0	129.6	92.6	0.564	53
d940821d5ec9	B intermediate, Fe low	single	4.1	27.5	0.0	0.067	27
db8b32da3c02	B high, Fe high	single	12.9	17.8	0.0	0.016	23
dc30a26428d0	B low, Fe high	single	0.0	0.0	36.0	0.167	21
dc9a4b2fe2ce	B high, Fe high	single	0.0	0.0	58.8	1.700	46
dcc61728d014	B intermediate, Fe low	single	0.0	5.8	17.9	0.056	37
ded0834854ab	B low, Fe high	two stage	0.0	0.0	97.5	0.767	56
e062d0f51406	B intermediate, Fe low	two stage	7.7	0.0	33.3	0.155	51
e162d895e9a9	B low, Fe high	two stage	0.0	2.7	30.1	0.364	47
e41bae89cab1	B intermediate, Fe high	two stage	0.0	0.0	169.3	1.571	42
e738e1b75626	B high, Fe high	single	24.6	0.0	16.4	0.157	67
e9ec256e3213	B low, Fe high	single	0.0	0.0	62.3	1.505	52
eb6a22dedbd1	B intermediate, Fe low	single	3.3	14.1	37.7	0.169	62
ee53fa8951b1	B intermediate, Fe low	single	0.8	23.3	72.9	0.284	54
eeae28102078	B low, Fe high	single	0.0	18.1	40.8	0.630	59
eee939d74e07	B intermediate, Fe low	two stage	0.0	4.6	21.3	0.025	26
ef4166ca3812	B low, Fe high	two stage	0.0	0.0	22.6	1.153	54
ef8b3df1c0ff	B intermediate, Fe high	two stage	0.0	13.5	42.3	0.123	52
efeae7f7fdb9	B low, Fe high	two stage	0.0	32.3	73.9	0.618	59
f0252793f87a	B low, Fe high	two stage	0.0	17.9	42.0	0.695	50
f16e65fa2083	B low, Fe high	single	0.0	3.2	27.7	1.018	57
f3203c597468	B intermediate, Fe low	single	7.3	6.2	0.0	0.005	23

f4c0d18bbc24	B intermediate, Fe low	single	0.0	19.5	152.9	0.934	64
fae2e5db36f9	B intermediate, Fe high	single	0.0	32.8	130.3	1.132	42
fb3937fbd0ab	B intermediate, Fe low	two stage	0.0	23.9	67.9	0.383	51
fbe7aaa27bf2	B intermediate, Fe low	single	0.0	5.1	32.9	0.051	25
fe4b81baa354	B intermediate, Fe low	single	0.0	20.8	54.7	1.604	57
ff55423fdca4	B intermediate, Fe low	single	0.0	0.0	43.8	0.642	42

Table S6 – SCAM specification: hyperparameters, shape constraints, and training/validation protocol. Shape-constrained additive spline models (SCAM; additive main effects) were fitted to predict $\log_{10}(\tau_K + \alpha)$ and $\log_{10}(\tau_D + \alpha)$ ($\alpha = 1\times10^{-12}$) for early and late conversion stages (split at X = 0.60), with an additional pooled check using a linear stage indicator. This table reports the spline basis/regularization, monotonicity constraints (direction/sign) per term, and the grouped cross-validation protocol.

**Item**	**Specification**
Model family	Shape-constrained additive spline model
Model structure	Additive main effects only; interactions were implemented using Generalized Additive Model plus Interactions (GA2M) model
Targets	$\log_{10}(\tau_K + \alpha)$ and $\log_{10}(\tau_D + \alpha)$, with $\alpha = 1\times10^{-5}$
SCM timescale extraction	NNLS incremental fit on $dt = t - t(X_{min})$ with τ_M, τ_K, $\tau_D \geq 0$; anchored at smallest X within each (experiment, stage) subset; $dt(X) = \tau_M\, g_M(X) + \tau_K\, g_K(X) + \tau_D\, g_D(X)$ with $\tau \geq 0$ (NNLS).
Stage definition	Early: X ≤ 0.60; Late: X > 0.60; Pooled check: both stages with a linear stage indicator
Minimum points	Per experiment (full trajectory window): ≥ 20; per stage: ≥ 12
Spline basis (per term)	Cubic B-splines (degree=3); monotone terms use integrated B-spline basis; free terms use centered B-spline basis
Predictor scaling	Each predictor is min-max scaled to [0,1] using the training set of the current fit (stored as x_{min}/x_{max} in the exported model); Values outside training range are clipped to [0,1]
Knot placement	Internal knots placed at empirical quantiles of the scaled predictor; n_{basis} may be reduced for small sample size/low unique values (final n_{basis} reported in term table)
Smoothness regularization	Second-difference penalty (order=2) on term coefficients; implemented by augmenting the least-squares system with $\sqrt{\lambda} \cdot D$
Regularization grid	λ ∈ logspace(-4, 4, 17) = [1.0e-04 … 1.0e+04], λ selected by minimizing OOF RMSE across folds
Training/validation split	Grouped 5-fold CV by experiment_id (entire experiments kept intact); not stratified; one-family-out validation by chem domain
Bootstrap uncertainty	Cluster bootstrap by experiment_id, B=200; uncertainty bands reported as quantiles (0.05, 0.95) of bootstrap predictions
Family-out generalization	See Table S7 for per-domain holdout metrics
Constraint mechanism	Monotonicity enforced via nonnegativity constraints on integrated-basis coefficients (per constrained term)

Table S7 – Term-level basis and monotonicity constraints used in the constrained SCAM fits. For each target (τ_K, τ_D) and stage (early: $X \leq 0.60$; late: $X > 0.60$; pooled check with a linear stage indicator), the table lists the included predictors, the spline basis type (integrated B-spline for monotone terms; centered B-spline for unconstrained terms; linear for stage code), and the imposed shape constraint with sign (monotone increasing "+" or monotone decreasing "−"). Reported n_{basis} values correspond to the effective basis size used after the script's small-sample/unique-value guard; all models use cubic splines (degree = 3) with second-difference smoothness regularization (penalty order = 2).

Target	Stage	Predictor (code)	Predictor	Basis	Constraint	Sign	n_{basis}	Degree	Penalty order
τ_D (transport timescale)	Early ($X \leq 0.60$)	Density	Pellet density, ρ_{pellet}	B-spline (centered)	Unconstrained		6	3	2
		Pellet diameter	Pellet diameter, D_{pellet}	Integrated B-spline	Monotone increasing	+	6	3	2
		Pore size	Pore size, d_{pore}	Integrated B-spline	Monotone decreasing	−	6	3	2
		Porosity stage	Porosity (stage median), ε_{stage}	Integrated B-spline	Monotone decreasing	−	6	3	2
		Tortuosity	Tortuosity, τ_{tort}	Integrated B-spline	Monotone increasing	+	6	3	2
	Late ($X > 0.60$)	Density	Pellet density, ρ_{pellet}	B-spline (centered)	Unconstrained		4	3	2
		Pellet diameter	Pellet diameter, D_{pellet}	Integrated B-spline	Monotone increasing	+	4	3	2
		Pore size	Pore size, d_{pore}	Integrated B-spline	Monotone decreasing	−	4	3	2
		Porosity stage	Porosity (stage median), ε_{stage}	Integrated B-spline	Monotone decreasing	−	4	3	2
		Tortuosity	Tortuosity, τ_{tort}	Integrated B-spline	Monotone increasing	+	4	3	2
	Pooled	Density	Pellet density, ρ_{pellet}	B-spline (centered)	Unconstrained		6	3	2
		Pellet diameter	Pellet diameter, D_{pellet}	Integrated B-spline	Monotone increasing	+	6	3	2
		Pore size	Pore size, d_{pore}	Integrated B-spline	Monotone decreasing	−	6	3	2
		Porosity stage	Porosity (stage median), ε_{stage}	Integrated B-spline	Monotone decreasing	−	6	3	2
		Tortuosity	Tortuosity, τ_{tort}	Integrated B-spline	Monotone increasing	+	6	3	2
		Stage code	Stage indicator (0=early,1=late)	Linear	Linear (unconstrained)		1	3	2
τ_K (reaction timescale)	Early ($X \leq 0.60$)	P_{gas}	Total pressure, P_{gas}	B-spline (centered)	Unconstrained		6	3	2
		Composition PC_1	Composition latent axis, Composition PC_1	B-spline (centered)	Unconstrained		6	3	2
		1/T	Inverse temperature, 1/T (K^{-1})	Integrated B-spline	Monotone increasing	+	6	3	2
		H_2	H_2 mole fraction, y_H_2 (–)	Integrated B-spline	Monotone decreasing	−	6	3	2
	Late ($X > 0.60$)	P_{gas}	Total pressure, P_{gas}	B-spline (centered)	Unconstrained		4	3	2
		Composition PC_1	Composition latent axis, Composition PC_1	B-spline (centered)	Unconstrained		4	3	2

		1/T	Inverse temperature, 1/T (K^{-1})	Integrated B-spline	Monotone increasing	+	4	3	2
		H_2	H_2 mole fraction, y__H$_2$ (–)	Integrated B-spline	Monotone decreasing	–	4	3	2
		P_{gas}	Total pressure, P_{gas}	B-spline (centered)	Unconstrained		6	3	2
		Composition PC_1	Composition latent axis, Composition PC_1	B-spline (centered)	Unconstrained		6	3	2
	Pooled	1/T	Inverse temperature, 1/T (K^{-1})	Integrated B-spline	Monotone increasing	+	6	3	2
		Stage code	Stage indicator (0=early,1=late)	Linear	Linear (unconstrained)		1	3	2
		P_{gas}	H_2 mole fraction, y__H$_2$ (–)	Integrated B-spline	Monotone decreasing	–	6	3	2

Table S8 – Family-out generalization performance by domain. Family-out evaluation results for predicting $\log_{10}(\tau_K)$ and $\log_{10}(\tau_D)$ (τ in minutes) across held-out domains under the final model specification (with Chem-PC). For each held-out domain, we report test-set sample sizes (number of experiments/rows) and error metrics (RMSE, MAE) computed on the $\log_{10}$ timescale targets. Cov80, Cov90, and Cov95 denote the empirical coverage of the nominal 80/90/95% predictive intervals on the held-out test set. Sample sizes are given as the number of held-out experiments and corresponding evaluation rows in the test set.

**Domain definition**	**Held-out domain (test set)**	**N_{test} expts (τ_K)**	**N_{test} rows (τ_K)**	**RMSE $\log_{10}(\tau_K)$**	**MAE $\log_{10}(\tau_K)$**	**Cov80 (τ_K)**	**Cov90 (τ_K)**	**Cov95 (τK)**	**N_{test} expts (τ_D)**	**N_{test} rows (τD)**	**RMSE $\log_{10}(\tau_D)$**	**MAE $\log_{10}(\tau_D)$**	**Cov80 (τD)**	**Cov90 (τD)**	**Cov95 (τD)**
Basicity-Fe bins	Basicity low; Fe bin 2	14	15	0.5039	0.3778	0.797	0.896	0.951	14	15	0.4875	0.4558	0.793	0.896	0.968
Basicity-Fe bins	Basicity low; Fe bin 3	10	10	0.5536	0.4641	0.814	0.9	0.949	10	10	0.4517	0.4049	0.80	0.91	0.98
Basicity-Fe bins	Basicity intermediate; Fe bin 1	14	17	0.5127	0.4348	0.841	0.91	0.98	14	17	0.5399	0.5757	0.820	0.915	0.957
G_a-G_b quadrants	G_a high; G_b high	15	18	0.6991	0.6361	0.811	0.907	0.944	15	18	0.5782	0.5517	0.813	0.904	0.955
G_a-G_b quadrants	G_a high; G_b low	11	12	0.5471	0.4104	0.833	0.916	0.95	11	12	0.4172	0.4776	0.833	0.916	0.949
G_a-G_b quadrants	G_a low; G_b low	14	14	0.4313	0.4132	0.894	0.90	0.951	14	14	0.4131	0.364	0.812	0.906	0.951
Compositio n domain	Basicity low; Fe high	24	25	0.5101	0.4022	0.808	0.901	0.947	24	25	0.5608	0.4562	0.82	0.92	0.978
Compositio n domain	Basicity intermediate; Fe low	14	17	0.5266	0.4501	0.824	0.905	0.97	14	17	0.6283	0.5767	0.809	0.905	0.948

Table S9 – Wrong-monotonicity falsification: the physically motivated monotonic constraints were intentionally sign-flipped (e.g., for τ_K: 1/T enforced decreasing and $p H_2$ enforced increasing; for τ_D: pellet diameter enforced decreasing), and the resulting degradation (or lack thereof) in grouped-CV performance is reported in $\log_{10}$ space.

Target	Stage	Correct monotonicity	Wrong monotonicity (flipped)	RMSE $\log_{10}$ (correct)	RMSE $\log_{10}$ (wrong)	ΔRMSE $\log_{10}$ ($RMSE_{wrong}$ – $RMSE_{correct}$)
τ_D	early	Pellet diameter ↑; (other transport terms as specified)	Pellet diameter ↓; (other transport terms as specified)	0.3661	2.0562	1.6901
	late			0.4742	1.5245	1.0503
	pooled			0.4263	1.8588	1.4325
τ_K	early	1/T ↑, $p H_2$ ↓; chem PC_1 unconstrained	1/T ↓, $p H_2$ ↑; chem PC_1 unconstrained	0.3185	1.3402	1.0217
	late			0.4997	2.0612	2.5615
	pooled			0.4362	1.8311	1.3949

Table S10 – Permutation negative controls: specified feature(s) (or predefined feature bundles) were randomly shuffled across experiments within each stage, while keeping the extracted targets ($\log_{10}\ \tau_K$, $\log_{10}\ \tau_D$), stage assignments, and all non-permuted covariates fixed. This breaks the physically meaningful feature–timescale coupling without altering trajectory-derived τ values (trajectory integrity), and performance is summarized over repeated runs (n_{runs}).

Target	Stage	Negative control	n_{runs}	RMSE ($\log_{10}$) mean	RMSE ($\log_{10}$) std
τ_D	early	Permutation control: shuffle pellet diameter only (within stage)	50	1.2516	0.716
		Permutation control: shuffle porosity (stage) only (within stage)	50	1.2250	0.813
		Permutation control: shuffle pore size only (within stage)	50	1.1652	0.756
		Permutation control: shuffle tortuosity only (within stage)	50	1.2183	0.864
		Permutation control: shuffle transport-feature bundle (Pellet diameter, Porosity, Tortuosity, Pore size, Density)	50	1.7289	0.606
	late	Permutation control: shuffle porosity/tortuosity only (within stage)	50	1.9437	0.778
		Permutation control: shuffle pellet diameter only (within stage)	50	1.9468	0.644
		Permutation control: shuffle porosity (stage) only (within stage)	50	1.9068	0.469
		Permutation control: shuffle pore size only (within stage)	50	1.9109	1.001
		Permutation control: shuffle tortuosity only (within stage)	50	1.9068	1.024
		Permutation control: shuffle transport-feature bundle (Pellet diameter, Porosity, Tortuosity, Pore size, Density)	50	2.0862	0.728
	pooled	Permutation control: shuffle porosity/tortuosity only (within stage)	50	1.9432	0.904
		Permutation control: shuffle pellet diameter only (within stage)	50	1.9442	0.864
		Permutation control: shuffle porosity (stage) only (within stage)	50	1.9192	0.924

		Permutation control: shuffle pore size only (within stage)	50	1.8974	0.81
		Permutation control: shuffle tortuosity only (within stage)	50	1.9088	0.871
		Permutation control: shuffle transport-feature bundle (Pellet diameter, Porosity, Tortuosity, Pore size, Density)	50	2.3498	0.911
T_K	early	Permutation control: shuffle 1/T only (within stage)	50	1.3176	0.869
		Permutation control: shuffle pellet diameter only (within stage)	50	1.3015	0.861
		Permutation control: shuffle reaction environment (1/T, H_2, P_{gas})	50	1.5755	0.971
		Permutation control: shuffle reaction-feature bundle (1/T, H_2, P_{gas}, chem_PC_1)	50	1.8121	0.997
	late	Permutation control: shuffle 1/T only (within stage)	50	2.1422	0.711
		Permutation control: shuffle pellet diameter only (within stage)	50	2.0805	0.633
		Permutation control: shuffle reaction environment (1/T, H_2, P_{gas})	50	2.1946	0.818
		Permutation control: shuffle reaction-feature bundle (1/T, H_2, P_{gas}, chem_PC_1)	50	2.1870	0.636
	pooled	Permutation control: shuffle 1/T only (within stage)	50	2.2370	0.848
		Permutation control: shuffle pellet diameter only (within stage)	50	2.2384	0.86
		Permutation control: shuffle reaction environment (1/T, H_2, P_{gas})	50	1.4684	0.872
		Permutation control: shuffle reaction-feature bundle (1/T, H_2, P_{gas}, chem_PC_1)	50	2.2646	0.883

Table S11 – Pareto candidate set summary (physics-only closure). Knee was selected as the Pareto compromise with minimum normalized Euclidean distance to the ideal point (min loss, min complexity) within the gated Pareto set.

Target	Physics variables used	Pareto set size (after gating)	Loss range	Complexity range	Selected knee (loss, complexity)
$\log_{10}\tau_K$	$\frac{T_{ref}}{T_{gas}+273.15}, \frac{p_{H_2}}{p_{ref}}$	31	0.089-0.183	6-22	0.1076, 11
$\log_{10}\tau_D$	$\frac{T_{ref}}{T_{gas}+273.15}, \frac{D_{pellet}}{d_{pore}}, \frac{\varepsilon}{\tau_{tort}}$	25	0.109-0.213	8-29	0.1259, 17

Table S12 – Family-out validation of the selected symbolic equations (composition-domain holdout). Performance is reported per held-out composition domain for (*i*) the physics-only symbolic term ("phys") and (*ii*) the full closure ("total", physics term plus the residual composition correction used in the final closure pipeline). Metrics include RMSE and MAE on the held-out data ($\log_{10}$-timescale units), along with held-out sample sizes (unique experiments n_{exp}; total stage rows n_{row}). Model selection followed a multi-objective Pareto "knee" rule (loss-complexity compromise); no additional nested CV was used beyond the family-out split.

Composition domain	$n_{\mathrm{exp,K}}$	$n_{\mathrm{row,K}}$	Total $\mathrm{RMSE_K}$	Total $\mathrm{MAE_K}$	Physics $\mathrm{RMSE_K}$	Physics $\mathrm{MAE_K}$	$n_{\mathrm{exp,D}}$	$n_{\mathrm{row,D}}$	Total $\mathrm{RMSE_D}$	Total $\mathrm{MAE_D}$	Physics $\mathrm{RMSE_D}$	Physics $\mathrm{MAE_D}$
Basicity low, Fe high	24	25	0.211	0.193	0.253	0.225	24	25	0.311	0.26	0.413	0.31
Basicity intermediate, Fe low	18	20	0.227	0.250	0.288	0.267	18	20	0.398	0.31	0.506	0.35
Overall	38	42	0.217	0.206	0.267	0.242	38	42	0.291	0.28	0.344	0.302

Table S13 – Quantile summary of illustrative process translation metrics derived from pellet scale observables under fixed operating assumptions. **Note:** Effective H_2 utilization and auxiliary recycle and conditioning energy were normalized to the median t(0.90) reference case under the fixed feed and fixed circulation assumptions defined in Section S3.

Quantile	t(0.90) (min)	t(0.90)-t(0.60)) (min)	Late stage fraction [t(0.90)-t(0.60)]/t(0.90)	Relative effective H_2 utilization	Auxiliary recycle and conditioning energy (MWh t^{-1} Fe)	Relative auxiliary energy
10th	10.551	5.295	0.406	1.967	0.282	0.490
25th	14.239	7.176	0.459	1.513	0.380	0.661
50th	21.541	11.724	0.531	1.000	0.575	1.000
75th	34.528	19.616	0.586	0.624	0.922	1.603
90th	60.013	32.866	0.655	0.359	1.602	2.786

Table S14 – Representative lower, median, and higher time cases selected from the compiled pellet scale dataset to illustrate the magnitude of the process translation. **Note:** The chemically required hydrogen at X = 0.90 was fixed at 48.6 kg H_2 t^{-1} Fe. Effective H_2 utilization and auxiliary recycle and conditioning energy were computed under the fixed feed and fixed circulation assumptions described in Section S3.

**Case**	**Experiment ID**	**t(0.90) (min)**	**t(0.90)-t(0.60) (min)**	**Late stage fraction**	**Relative effective H_2 utilization**	**Auxiliary recycle and conditioning energy (MWh t^{-1} Fe)**	**Relative auxiliary energy**
Lower time representative case	1846a6156349	5.001	1.966	0.393	2.156	0.133	0.232
Median reference case	37ce7bad2c0d	21.541	11.724	0.544	1.000	0.575	1.000
Higher time representative case	1f2fd7e87449	200.004	130.780	0.654	0.109	1.539	2.676

Table S15 - Relation between the effective characteristic times inferred in the present study and the mechanistic characteristic-time terms used in the multiscale additive-time framework of Hamadeh et al. The table summarizes the closest physical correspondence between the effective pellet scale characteristic times used in the present study and the forward mechanistic characteristic-time expressions reported by Hamadeh et al. The comparison is intended at the level of physical interpretation, scale of description, and expected direction of dependence, not as a term by term numerical equivalence. In Hamadeh et al., the characteristic times are expressed as forward mechanistic equations for separately resolved reaction and transport sub-contributions within a multiscale pellet-and-shaft furnace model. In the present study, by contrast, τ_M, τ_K, and τ_D are inferred directly from measured reduction trajectories t(X), and the symbolic laws in Table 2 (main text) represent stage-resolved effective pellet-scale constitutive closures within the sampled experimental envelope.

Quantity	Closest counterpart in Hamadeh et al.	Shared physical meaning	Expected direction of dependence	Level of description	What is not directly comparable
τ_M	External transfer characteristic time	External gas film mass transfer resistance	Decreases with stronger external transfer conditions	**Present study:** an effective inferred pellet scale quantity. term-by-term<br><br>**Hamadeh:** forward mechanistic transport term	The present study does not isolate a mechanistic gas film coefficient or reconstruct the reactor side transfer field
τ_K	Chemical reaction characteristic times for the reduction steps	Interfacial chemical reduction limitation	Decreases with increasing temperature and a stronger driving force for reducing gases	**Present study:** effective stage-resolved pellet scale reaction timescale inferred from t(X).<br><br>**Hamadeh:** step-specific forward mechanistic reaction times	The present study does not resolve reaction characteristic times separately for each oxide reduction step
τ_D	Intergranular, intragranular, intercrystallite, and intracrystallite diffusion characteristic times	Internal transport limitation through pellet and product structure	Increases with larger transport length scales and reduced pore accessibility	**Present study:** net pellet scale internal transport descriptor inferred from t(X).<br><br>**Hamadeh:** separately resolved internal diffusion sub contributions across structural scales	The present study does not partition internal transport into structural sub-mechanisms or assign separate forward diffusion terms
